# Integrated Life Cycle Assessment Guides Sustainability in Synthesis: Antiviral Letermovir as a Case Study

**DOI:** 10.1021/jacs.5c14470

**Published:** 2025-10-27

**Authors:** Sander Folkerts, Maximilian G. Hoepfner, Gonzalo Guillén-Gosálbez, Javier Pérez-Ramírez, Erick M. Carreira

**Affiliations:** † Department of Chemistry and Applied Biosciences, Laboratory of Organic Chemistry, ETH Zürich, 8093 Zürich, Switzerland; ‡ Department of Chemistry and Applied Biosciences, Institute for Chemical and Bioengineering, ETH Zürich, 8093 Zürich, Switzerland; § NCCR Catalysis, Zürich CH-8093, Switzerland

## Abstract

The identification of metrics to assess the sustainability of complex chemical synthesis routes has been a topic of interest in recent years. The diversity of life cycle assessment (LCA) approaches for fine chemicals and pharmaceuticals that have been developed face a common challenge: limited availability of production data. This critically affects completeness, accuracy, and reliability. Herein, we describe an iterative closed-loop approach, bridging life cycle assessment and multistep synthesis development. Our comprehensive analysis leverages documented sustainability data augmented by information extrapolated from basic chemicals through retrosynthesis. The LCA results are discussed and evaluated against the more traditional process mass intensity (PMI). We have chosen the synthesis of the commercial antiviral drug Letermovir as a case study: implementation of LCA to the published route in parallel to a de novo synthesis enables us to benchmark, compare, and contrast routes. Identification of bottlenecks in both syntheses revealed negative impacts on the sustainability in asymmetric catalysis as well as metal-mediated couplings, highlighting the continued demand for sustainable catalytic approaches that minimize adverse effects on global warming potential, ecosystem quality, human health, and natural resources. This comprehensive strategy for multilevel sustainability assessment increases the accuracy, facilitates comparisons, and enables targeted optimization of sustainability in organic chemistry.

## Graphical abstract

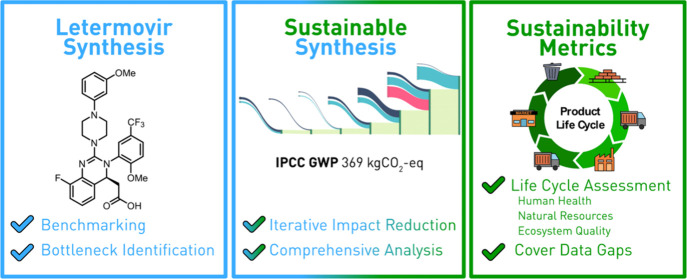

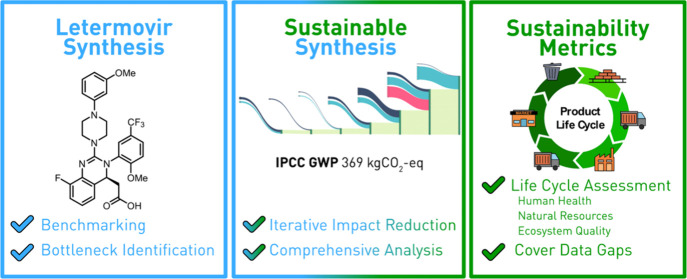

## Introduction

Active pharmaceutical ingredients (APIs) are high-value consumer goods that result from research-intensive processes in drug discovery and development.^1^ APIs are typically complex molecular structures whose manufacturing process involves multistep syntheses. Route design and selection have traditionally focused on strategic convergence of reactions alongside economic considerations.^2^ The concepts of sustainability have been gaining attention in route optimization and selection.^3–5^ Previously, in 1998, Anastas and Warner introduced the 12 principles of green chemistry that are widely accepted and have been instrumental to the discipline.^6^ A variety of metrics-based approaches have been subsequently introduced to assess sustainability of synthesis strategies.^7^ Standard indicators for API syntheses include mass-based metrics,^8–12^ such as process mass intensity (PMI),^13,14^ atom-economy (AE),^15^ E-factor (E),^16,17^ solvent intensity (SI),^18^ and carbon-economy (CE).^19^ The focus has recently shifted toward life cycle assessment (LCA) because it encompasses a broader scope that considers the entirety of the chemicals’ supply chain and production (Figure 1).

**Figure 1:**
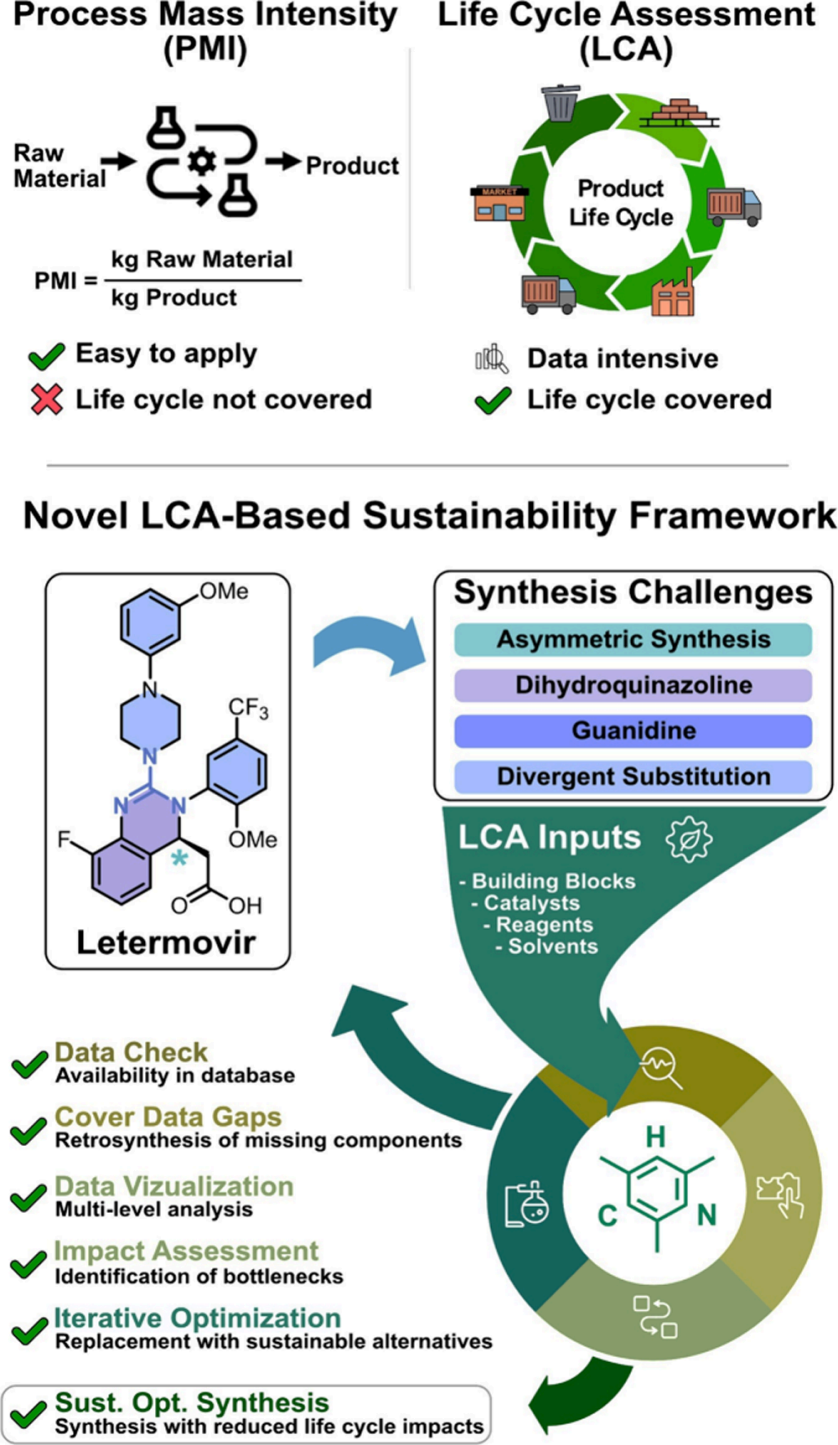
Structural analysis of Letermovir as a synthetic case study and the development of an LCA-guided synthesis approach.

LCA is a process that is significantly more data- and time-intensive compared to the standard green metrics. Nevertheless, recent studies underscore the need to include LCA to draw more holistic conclusions, leading to enhanced sustainability outcomes.^20,21^ LCA adds value because it provides more nuanced insights by augmenting green metrics with the inclusion of indicators that capture influence on human health (HH), natural resources (NR), ecosystem quality (EQ), and global warming potential (GWP).^20^ The identification of sustainable synthesis routes based on LCA hinges on the quality of the underlying data, involving the various components in the chemical supply chains.^23^ For bulk chemical production, LCAs are widely used in industry, and the data are readily available.^22^ However, the same is not true for the synthesis of fine chemicals, where published evaluations tend to rely largely on traditional green chemistry metrics.^23^ In more recent developments, the pharmaceutical industry has started to adopt LCA for evaluating synthesis process routes for active pharmaceutical ingredients (APIs); however, few LCAs have been reported to date.^2^ For maximum benefits, LCA should be implemented in the early design stages of synthesis planning. At later stages of process development, adopted production setups tend to constrain modification possibilities.^24^ LCA-guided synthesis planning should ideally be adopted as an iterative enhancement loop.

In this study, we introduce a new LCA workflow that facilitates the analysis of multistep synthesis routes to complex molecules. We examine its use in the context of synthesis routes to Letermovir (Figure 1), an antiviral drug targeting human cytomegalovirus (HCMV). Letermovir was developed by Merck & Co., Inc. and has been approved by the FDA and EMA for prophylactic use against HCMV infections in stem cell transplant patients and more recently also in kidney transplant patients.^25^ Prevymis (brand name of Letermovir) reached retail sales of $605 million in 2023.^26^

We chose to investigate the synthesis of Letermovir as a case study due to several embedded structural features that reflect modern challenges in the synthesis of optically active pharmaceuticals. The target molecule is nitrogen-rich, featuring a fully substituted guanidine at its core that is part of a fluorinated dihydroquinazoline and incorporates a stereogenic center (Figure 1). Letermovir also features an *N*-arylated piperazine along with a trifluoromethyl substituted aniline. Importantly, the manufacturing process of Letermovir was bestowed with the 2017 Presidential green chemistry challenge award from the US Environmental Protection Agency (EPA).^27^ As such, by selecting the Letermovir synthesis as a case study for the LCA workflow, we commenced with a highly advanced, optimized benchmark. Accordingly, the analysis of the known route in parallel with the development of a novel synthesis allows the evaluation of our LCA workflow in comparing and contrasting sustainability considerations.

The LCA of the published synthetic approach reveals a critical hotspot displaying high environmental impact: the Pd-catalyzed Heck cross coupling of an aryl bromide with an acrylate.^28,29^ Additionally, an enantioselective 1,4-addition required the generation of a life cycle impact inventory for the biomass-derived phase-transfer catalyst (cinchonidine derived). In performing LCA-guided multistep synthesis of Letermovir, we integrate in silico ex ante LCA calculations with experimental work, providing full transparency on broad sustainability implications of decisions taken during synthesis planning. For the route developed in the context of this study, the hotspot is a novel, enantioselective Mukaiyama–Mannich addition, employing chiral Brønsted-acid catalysis. The use of a boron-based reduction of an anthranilic acid addressed the negative environmental influence of a LiAlH_4_ reduction as the first step in an early exploratory route. LCA revealed that a Pummerer rearrangement provides a beneficial alternative to access an aldehyde oxidation state of a key intermediate. Both the de novo and the published Merck route^28^ suffered from the need for large solvent volumes for purification.^30^ The described LCA approach highlights that substantial environmental savings can be obtained through targeted actions along the synthesis route. Accordingly, the value-added proposition of LCA is its application for benchmarking emerging routes with existing ones and identifying hotspots that ultimately pave the way to an optimal sustainable process.

## Method and Background

Several tools based on standard green chemistry metrics have been reported. Recently, Wuitschik and co-workers at Roche introduced ChemPager: this tool incorporates the SMART-PMI predictor of the ACS Green Chemistry Institute Pharmaceutical Roundtable (ACS GCIPR), which evaluates and compares chemical syntheses with a focus on process-chemistry relevant information.^31,32^ Gallou and co-workers at Novartis developed a green chemistry process scorecard to evaluate the environmental impacts of API production processes, featuring a total CO_2_ release calculated from the PMI.^33^ In collaboration with the ACS GCIPR, Rose, Kosjek, and co-workers at Merck developed a PMI-LCA tool that expands green chemistry analysis with life cycle assessment. The Merck approach only accurately accounts for chemicals found in databases (e.g., ecoinvent); however, the individual life cycle inventories (LCIs) of chemicals that are not found in the database are not considered in the analysis.^34–36^ The Fast Life Cycle Assessment of Synthetic Chemistry (FLASC) tool, developed by Jiménez-González and co-workers at GSK, is another example of LCA based approaches for the incorporation of sustainability analyses in the synthesis of APIs. The FLASC approach, however, suffers fundamentally from insufficient data availability. In that respect, data gaps are bridged by employing compound class-averages as proxy in lieu of empirical data, detrimentally affecting accuracy of this approach.^37,38^ Alternative scoring systems have been proposed in the literature to account for the effects on toxicity of the materials (reagents, reactants, additives, and solvents) used.^8,19,36,39–50^

Traditional LCA is hampered by incomplete databases of the chemical inventory. The use of LCA rapidly reaches its limits when dealing with compounds absent from the database. For example, while the full details of diisopropyl amine or dimethylamine are included in the ecoinvent database, downstream products such as LDA or EDC are not. Under such circumstances the current LCA approaches would exclude LDA and EDC from the analysis or at best rely on proxy data or estimates, thereby leading to less accurate conclusions.^7,34,37,38^ This is particularly relevant for multistep syntheses of complex molecules (fine chemicals, pharmaceuticals, etc.), where a substantial proportion of the data for intermediates, catalysts, reagents, and solvents may be missing in existing LCA databases. As an example, ecoinvent, a leading LCA database, covers merely 1000 chemicals,^51^ underscoring the high likelihood of facing data gaps in the LCA of APIs. Consequently, we address this limitation by the use of an iterative retrosynthetic approach that considers literature reported experimental data for the calculation of the individual LCIs of missing chemicals from the database.

Our study aims at new route designs for Letermovir, whereby (retro)­synthesis is guided and enhanced by iterative closed-loop LCA. In the initial data availability check (Phase 1, ①) of the workflow (Figure 2), we identified that only 20% of the chemicals used in the first iteration of the synthesis were found in ecoinvent v3.9.1–3.11. We provide an example that illustrates the adversities encountered during the workflow. Synthetic considerations led to the identification of starting materials **IV** (Figure 2, X = NH_2_, Br), which were absent from the ecoinvent database. To build the necessary data for **IV** (X = NH_2_) further retrosynthetic analyses were performed leading to **I** as starting material, which is found in ecoinvent. Details of published industrial routes from *p*-xylene (**I**) to **IV** were used to extract reaction conditions to integrate the data into LCA (see SI). In order to scale the system to the requisite functional unit (FU) of 1 kg, back-calculation of required masses for all compounds in all steps of the synthesis were carried out. The life cycle inventory (LCI) data for all chemicals for the synthesis of **IV** are tallied to build its corresponding entry (see SI). This procedure is iterated for all undocumented chemicals involved in the synthesis of the API (i.e., Letermovir). This approach ensures a comprehensive and meaningful analysis without neglecting the individual influence of any chemicals and their implications for the API synthesis.

**Figure 2:**
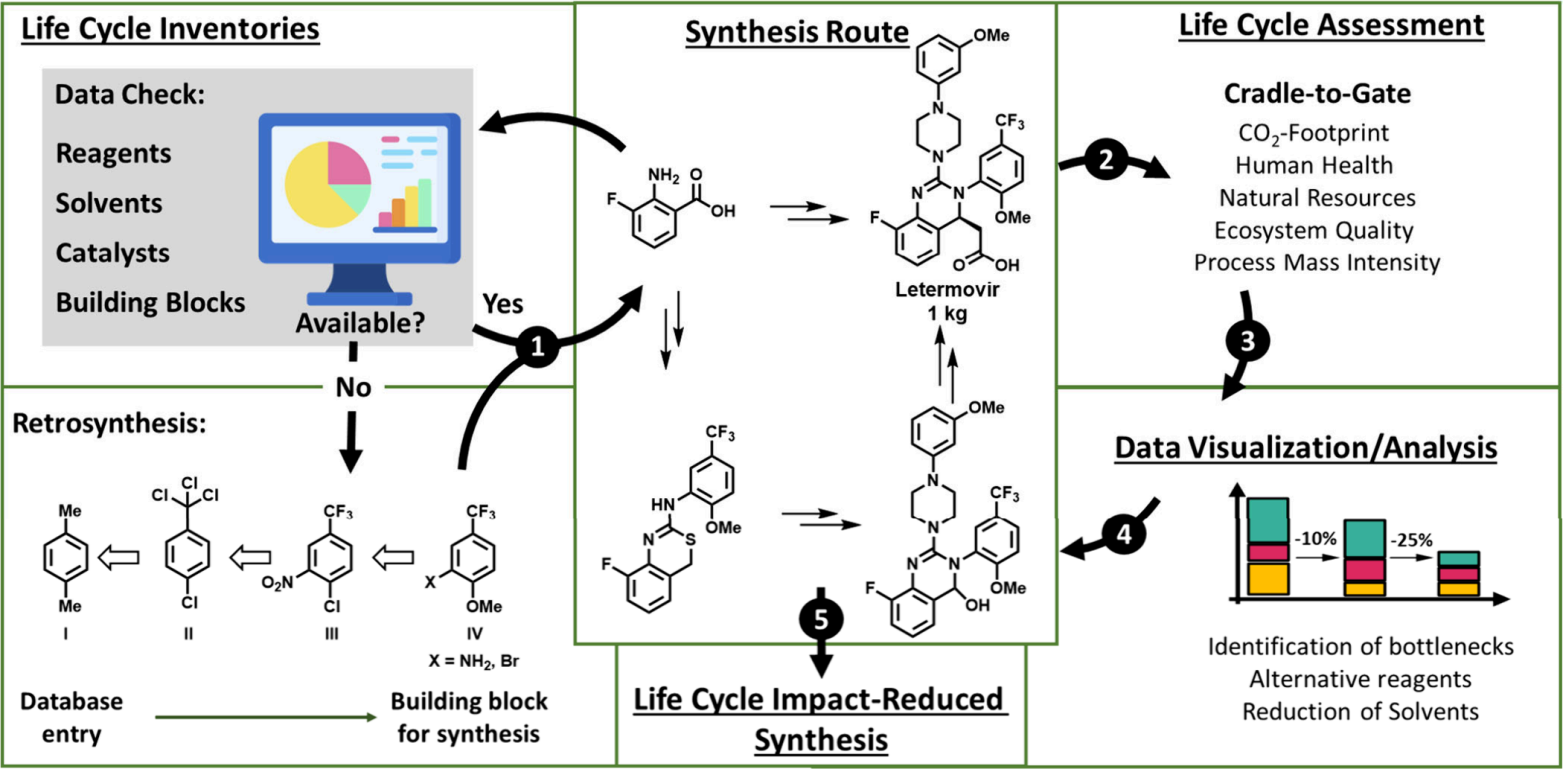
Flowsheet of LCA guided analysis for optimization of API synthesis. Phase 1 (①) Data Check: check availability of data in ecoinvent. Cover Data Gaps: retrosynthesis to reconstruct data for missing chemicals. Phase 2 (②) Life Cycle Assessment: LCA of the synthesis based on IPCC 2021 GWP 100a and ReCiPe 2016. Phase 3 (③) Data Visualization: multilevel analysis with various diagrams and plots. Phase 4 (④) Iterative Optimization: identification of steps and chemicals with high life cycle impacts and replacement by more sustainable alternatives. Phase 5 (⑤) Life Cycle Impact Reduced Synthesis: provide a sustainability optimized synthesis.

LCA calculations (Phase 2, ②) were implemented in Brightway2 using Python. We considered a cradle-to-gate scope for the production of 1 kg of Letermovir, focusing on climate change (IPCC 2021 GWP100a) and the ReCiPe 2016 end points (human health HH, ecosystems quality EQ, and depletion of natural resources NR).^52–54^ Total greenhouse gas emission of a chemical over its entire life cycle is expressed in terms of CO_2_ equivalents (CO_2_-eq). Global warming potential (GWP, measured in kgCO_2_-eq) accounts for all greenhouse gases by converting their warming effects into an equivalent amount of CO_2_ for standardized comparison. The results of the calculations are visualized in different diagrams (Phase 3, ③) that allow for a multilevel analysis. This visualization enables the evaluation of each step as well as each chemical used in the synthesis of the API. The GWP contributions of each step are further categorized into reagents, solvents, and catalysts. This differentiation facilitates stepwise assessment and provides the means for sensitivity analyses of catalyst recovery rates (RRs). Based on the calculated data, hotspots are identified (Phase 4, ④), and targeted actions to mitigate them can be defined (e.g., selecting alternative reagents or solvents). The optimization is repeated iteratively, focused on the feasibility of the underlying chemistry in each step, to obtain a synthesis with an optimized sustainability profile (Phase 5, ⑤). For comparison and benchmarking, the approach was implemented for the de novo synthesis of Letermovir as well as the published route by Merck.

## Results and Discussions

To showcase the value of the LCA formulated in this study, in a real-life academic or industry research scenario, we selected Letermovir as a target for synthesis. The LCA tool was implemented throughout the workflow. As such it facilitates benchmarking, comparing, and contrasting with the published route. The workflow readily identifies bottlenecks, prompts innovation, and informs route selection as well as optimization.

### Exploratory Synthesis of Intermediates

In the retrosynthetic analysis, we prioritized the asymmetric synthesis step, noting that it would be advantageous to introduce the stereocenter at a late stage in the synthesis route. Steps in asymmetric synthesis are typically anticipated to be laborious and costly. Moreover, the attendant increased value of the optically active products dictates that they be taken through a minimal number of subsequent steps to the target.^55^ The introduction of the C(4)-acetic acid substituent on the dihydroquinazoline core was envisioned to be conducted via Mukaiyama–Mannich addition (Scheme 1A, ***i***). A key benefit to this strategy is that Letermovir methyl ester is the penultimate intermediate in Merck’s published synthesis, and it had been demonstrated that recrystallization as the (−)-di-*p*-toluyl-l-tartaric acid ((−)-DTTA) salt enriches optical purity. We envisioned hemithioacetal ***ii*** as a precursor for ***i*** (Scheme 1A).

**Scheme 1:**
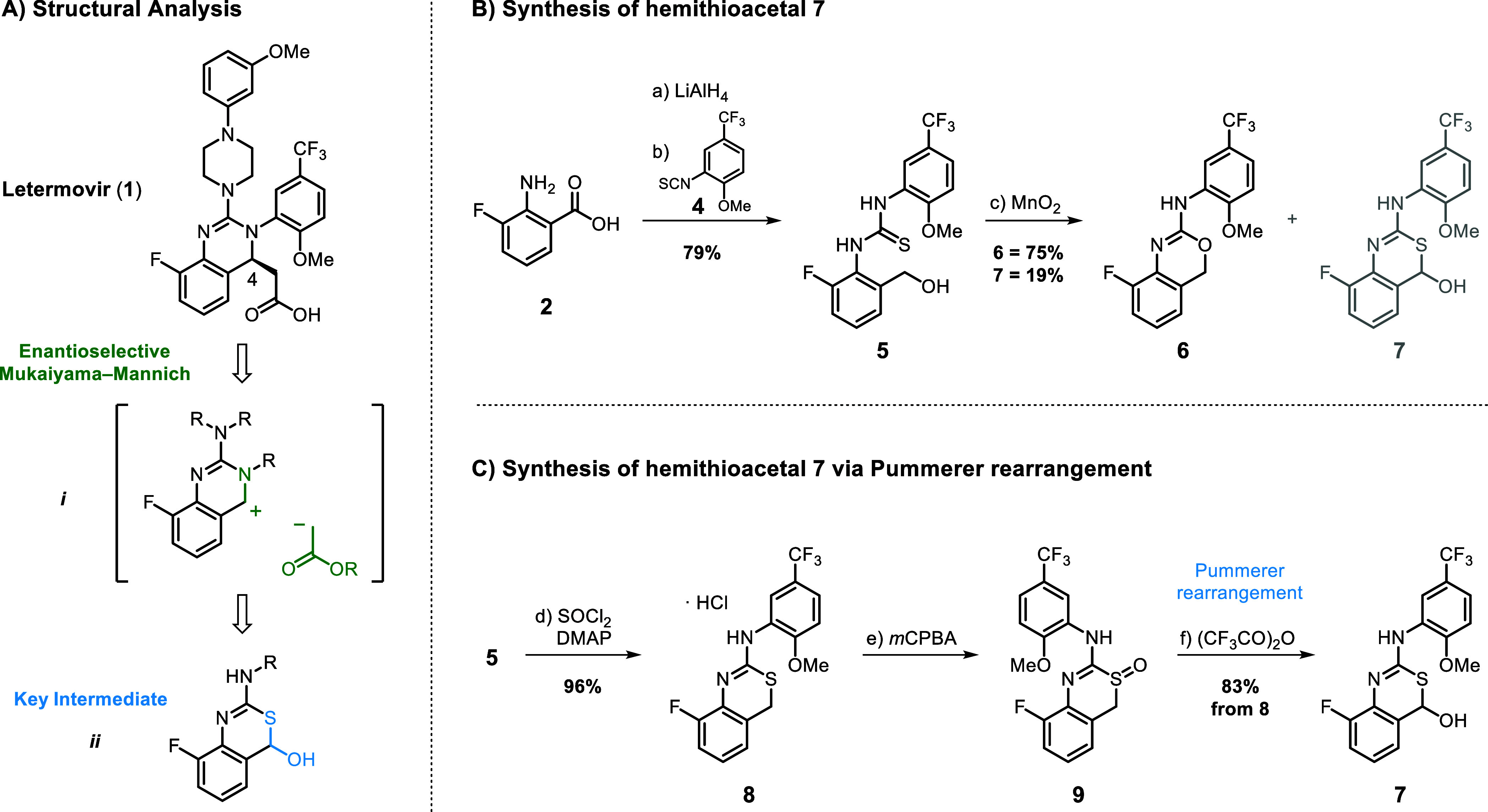
Identification of Synthetic Strategy for **1** ^a^

a Reagents and conditions: (a) LiAlH_4_, THF (0.1 M), 99%; (b) **4**, MeCN, 80%; (c) MnO_2_, CH_2_Cl_2_, 75% **6** and 19% **7**; (d) SOCl_2_, DMAP (1 mol %), MTBE, 0 °C, 96%; (e) *m*CPBA, CH_2_Cl_2_, 0 °C; (f) TFAA, CH_2_Cl_2_, 0 °C to rt, 83% over two steps.

The synthetic efforts commenced with the synthesis of hemithioacetal **7** (Scheme 1B) from 3-fluoro-anthranilic acid **2**. LiAlH_4_ reduction of carboxylic acid in **2** delivered the corresponding benzylic alcohol which, upon treatment with isothiocyanate **4**, formed thiourea **5** in 79% yield over two steps. MnO_2_-mediated oxidation delivered mainly isourea **6** in 75% yield along with only 19% of hemithioacetal **7**. We hypothesize that *S*-oxidation of the thiourea leads to activation toward intramolecular substitution by the benzylic alcohol to deliver **6**. A broad range of oxidants were tested, including DMP, DDQ, PCC, TEMPO/NaOCl, TPAP/NMO, or SO_3_·Py/DMSO, but no improved conditions were identified. We then set out to investigate whether we could leverage the proclivity of sulfur for oxidation to form the hemithioacetal via Pummerer rearrangement (Scheme 1C). To this end, treatment of benzylic alcohol **5** with SOCl_2_ triggered cyclization to isothiourea **8**. *S*-Oxidation with *m*CPBA in CH_2_Cl_2_ delivered thiazine-oxide **9**, and direct treatment with TFAA furnished product **7** in 83% yield over two steps. Analysis of the ^1^H NMR spectrum of **7** revealed only the presence of hemithioacetal **7** (Scheme 1C), and no characteristic aldehyde peak was detected.

### LCA and Synthesis Optimization

We discuss comprehensively how the reaction conditions of each step in the synthesis affect the global warming potential (GWP). For each step, a base case is established in which the optimized synthesis (*vide infra*) serves as a default starting point. Individual reaction conditions of the steps were then varied to assess their isolated effects to mitigate GWP contributions. As anticipated, a significant effect of type and quantity of solvents was observed on the GWP impact. The closed-loop approach, linking synthesis optimization with LCA, uncovered insights beyond the obvious and identified non-intuitive impact hotspots.

***Step 1***. Reduction of 3-fluoro anthranilic acid **2** showed a vast range of GWP impacts depending on the reaction conditions. LiAlH_4_ reduction when conducted in 2-Me-THF (2.9 kgCO_2_-eq/kg_solvent_), instead of THF (8.0 kgCO_2_-eq/kg_solvent_),^51,56^ reduced the GWP impact by 103 kgCO_2_-eq/kg. An increase in concentration from 0.16 to 0.64 M reduced it further by 44 kgCO_2_-eq/kg. Closer evaluation revealed that the increased CO_2_-eq emission and depletion of natural resources is primarily linked to the environmental impact caused by mining and extraction of lithium in LiAlH_4_, despite its use as a low-cost reductant in reaction processes.^57–59^ Consequently, we looked for an alternative reductant with a better sustainability profile. In initial experiments, commercially available 1 M BH_3_·THF reduced anthranilic acid **2** to primary alcohol **3** (99% yield).^57,60^ The BH_3_·SMe_2_ complex, by comparison, is an alternative, solvent-free BH_3_ source. The reduction of 3-fluoro-anthranilic acid **2** was effected with 1.2 equiv of BH_3_·SMe_2_ in 2-Me-THF (1.1 M) in 99% yield. By replacing LiAlH_4_ with BH_3_·SMe_2_, the GWP impact of the first step was further reduced by 54%; this is equivalent to an absolute decrease of 27 kgCO_2_-eq/kg in the synthesis (Scheme 2, Step 1). Notably, even after several iterative improvements to reduce the GWP impact, the LCA continued to guide the optimization process toward non-obvious impact hotspots, highlighting the use of BH_3_·SMe_2_ as a lower impact alternative for the reduction in Step 1.

**Scheme 2:**
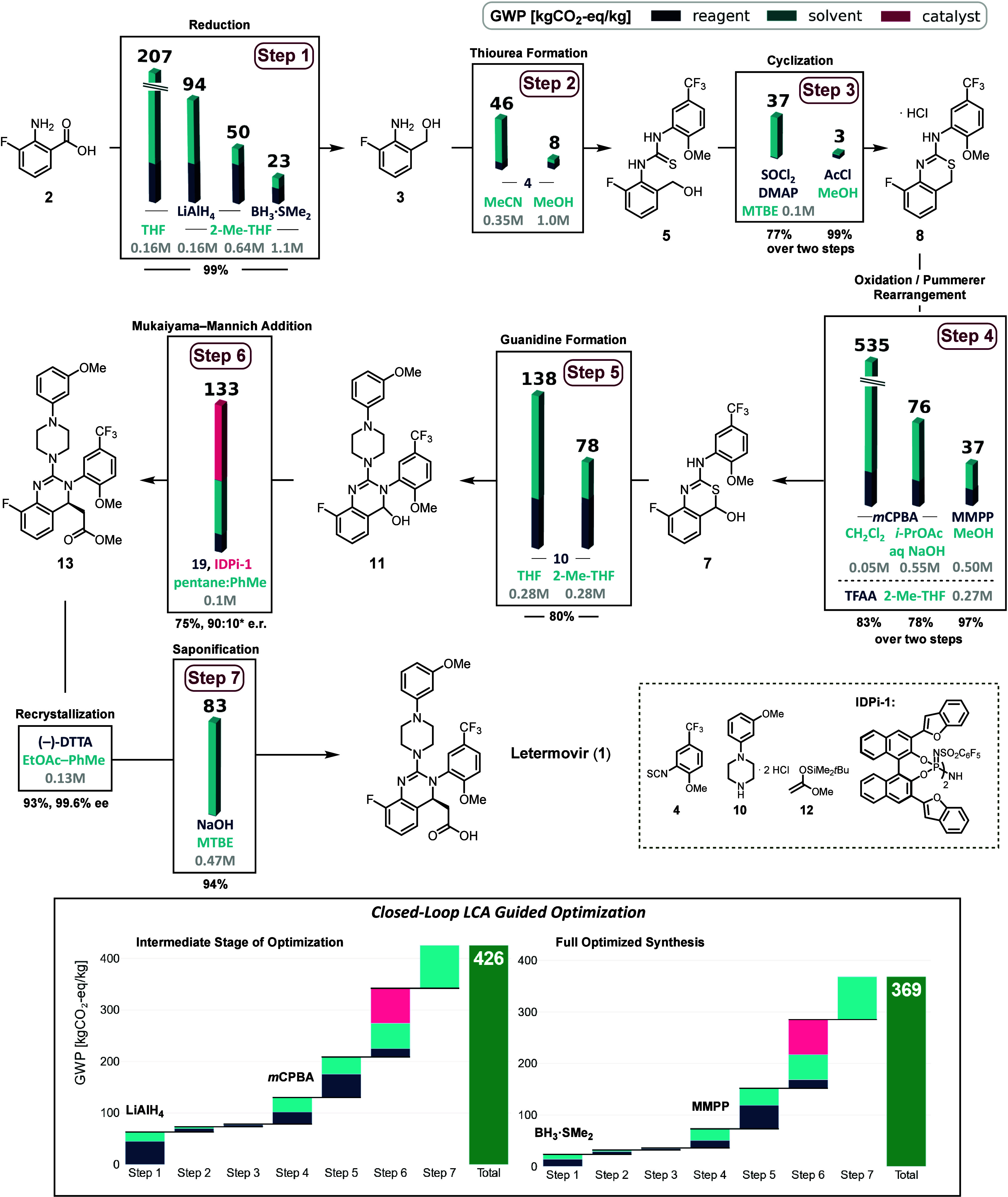
Our Synthesis of Letermovir with LCA-Guided Optimizations Based on GWP^a^

a Reagents and conditions for final route: **Step 1**: BH_3_·SMe_2_ (1.20 equiv), 2-Me-THF (1.10 M), 80 °C, 99%; **Step 2**: **4** (1.05 equiv), MeOH (1.00 M), r.t.; **Step 3**: AcCl (1.5 equiv) in MeOH, r.t., 98% over two steps; **Step 4**: MMPP (0.70 equiv), MeOH (0.50M), r.t., then 2-Me-THF (0.50 M), Et_3_N (1.00 equiv), TFAA (1.15 equiv), 97% over two steps; **Step 5**: **10** (1.05 equiv), Et_3_N (4.40 equiv), EDC·HCl (1.10 equiv), 2-Me-THF (0.28 M), 80%; **Step 6**: **IDPi-1** (1.0 mol %), *sec*-BuOH (1.0 equiv), **12** (3.0 equiv), r.t., *n*-pentane–PhMe (1:1, 0.10 M), 75%, 90:10 er then (−)-DTTA, PhMe–EtOAc, 45 °C, 93%, 99.6% ee; **Step 7**: MTBE, Na_2_HPO_4_, 1 M aq NaOH, 60 °C, 94%. *The er for step 6 could be increased up to 97:3 by running the reaction under dry and inert conditions. It requires additional PhMe to remove residual water from the starting material before use in the reaction.

***Steps 2 and 3***. The conditions described previously to form thiourea **5** in MeCN and cyclization to isothiourea **8** triggered by SOCl_2_ and DMAP in MTBE were optimized to a one-pot protocol. Compounds **3** and **4** were dissolved in MeOH (1 M), and upon full conversion to thiourea **5**, a solution of AcCl in MeOH was added dropwise to trigger cyclization to isothiourea **8**. This adjustment reduced the overall amount of solvent and improved the yield to 98% over two steps, reducing the GWP impact by 72 kgCO_2_-eq/kg (Scheme 2, Steps 2–3).

***Step 4***. For the initial conditions of *S*-oxidation and rearrangement (*m*CPBA, CH_2_Cl_2_ (0.05 M), followed by TFAA) to obtain hemithioacetal **7**, an exorbitantly high GWP value of 535 kgCO_2_-eq/kg was calculated. This is primarily due to high dilution of the reaction and, to a lesser extent, the use of CH_2_Cl_2_ as solvent. As part of a first iteration and counterintuitively, the reaction sequence was optimized from a one- to a two-pot procedure. Biphasic reaction conditions (*i*-PrOAc–aq NaOH) for the *S*-oxidation with *m*CPBA, followed by solvent swap to 2-Me-THF and treatment with TFAA, delivered hemithioacetal **7** in overall 78% yield. The two-pot sequence resulted in a substantial GWP impact reduction of 459 kgCO_2_-eq/kg, largely due to increased concentrations (0.55 M) and, to a lesser extent, replacement of CH_2_Cl_2_ with *i*-PrOAc and 2-Me-THF (Scheme 1, Step 4). The closed-loop between LCA and synthesis of the *S*-oxidation step revealed that magnesium monoperoxyphthalate (MMPP) offers a lower GWP impact (3 kgCO_2_-eq/kg) than *m*CPBA (10 kgCO_2_-eq/kg) and serves as an effective alternative oxidant. A further challenge was the separation of the desired thiazine-*S*-oxide from *m*-chlorobenzoic acid, a byproduct of *m*CPBA mediated oxidations. Switching to MMPP and using MeOH as solvent yielded quantitatively the *S*-oxide **9** with a simple workup via a basic aqueous wash, eliminating the need for further purification. Substitution of *m*CPBA with MMPP and attendant increase of yield of **7** (78% to 97%) resulted in a 51% decrease of the GWP, totaling 39 kgCO_2_-eq/kg (Scheme 2, Step 4). In summary, for this step, LCA-guided optimization revealed two non-obvious improvements: the greater efficiency of a two-pot procedure and the benefits of MMPP over *m*CPBA.

***Step 5***. As anticipated, hemithioacetal **7** could be activated with EDC·HCl in the presence of piperazine·2HCl **10** under basic conditions to obtain hemiaminal **11**. For this step, reduction of the GWP value of 60 kgCO_2_-eq/kg (43%) was accomplished by substitution of THF with 2-Me-THF.

***Steps 6 and 7***. With intermediate **11** in hand, the introduction of the C(4)-acetate side chain was explored for the first time using ketene acetal **12** under conditions involving bistriflimide as a Brønsted acid catalyst. We observed formation of Letermovir methyl ester **13** in 45% yield as a racemate. After extensive testing of various catalysts (see Table S7 in SI) enantioinduction was observed with chiral imidodiphosphorimidates (IDPis).^61–65^ In the study of this reaction, we observed the adduct corresponding to Mukaiyama–aldol addition. Presumably under reaction conditions, an aldehyde is transiently generated and intercepted by silyl ketene acetal **12**. In related work by Peng, it was noted that the addition of *sec*-BuOH resulted in increased yield and optical purity of the β-amino ester products.^62^ Accordingly, when we employed 1 equiv of *sec*-BuOH under otherwise identical conditions, only Mannich product **13** was obtained. Catalyst **IDPi-1** in combination with *n*-pentane:PhMe (1:1) and *sec*-BuOH additive were identified as optimal (75% yield, 90:10 er, rt).^66,67^ With the generation of an optically active product, this step significantly increases complexity and displays a GWP impact of 133 kgCO_2_-eq/kg.

Drying the starting material by azeotropic removal of adventitious water with toluene resulted in improved enantiomeric ratios of 97:3. Subsequently, mechanistic investigations of the enantioselective Mannich reaction suggest in situ formation of an *O*-*sec*-Bu-hemiaminal derived from **11** (see **S1** and additional details in SI) as a competent intermediate en route to **13**.^62,68^ Notably, this transformation leads to high enantiomeric ratio at room temperature, whereas comparable additions are frequently reported at significantly lower temperature (−45 to −95 °C).^62,65,69–71^ To the best of our knowledge, this is the first enantioselective Mukaiyama–Mannich addition with a silyl ketene acetal catalyzed by an IDPi.^69^ The protocol by Merck researchers was implemented to increase optical purity of methyl ester **13** by recrystallization with (−)-DTTA from PhMe:EtOAc.

In Step 7, methyl ester **13** was saponified with aq NaOH in MTBE to complete the synthesis of Letermovir **1**. This reaction produced a calculated GWP of 83 kgCO_2_-eq/kg. These conditions were adopted from Merck’s synthesis to ensure comparable final quality (93% yield, >99% ee) of Letermovir.^72^ Following a comprehensive evaluation of the GWP impact from each individual step relative to their optimization status, we visualized the synthesis’s gradual increases of GWP at both an intermediate and fully optimized stage using waterfall diagrams (Scheme 2, bottom). The closed-loop approach, integrating synthesis optimization with LCA, revealed unexpected impact hotspots in Steps 1 and 4. The LCA-guided optimizations of reagent selection in these steps significantly improved the overall profile of the GWP across the first five steps.

### Insights from Overarching LCA and Comparison with Optimized Synthesis

With the LCA-guided optimized synthesis established (Scheme 2), we carried out a comprehensive LCA and contextualized the results by comparison with the published Letermovir route by Merck. In 2016 Humphrey, Dalby, and co-workers reported the asymmetric synthesis of Letermovir, with an enantioselective intramolecular 1,4-addition as the key step to form the dihydroquinazoline core including the C(4)-acetate stereogenic center (Scheme 3).^28^ A reported overall yield of >60% along with streamlined purification and solvent use represents a compelling benchmark for LCA-based comparison.

**Scheme 3:**
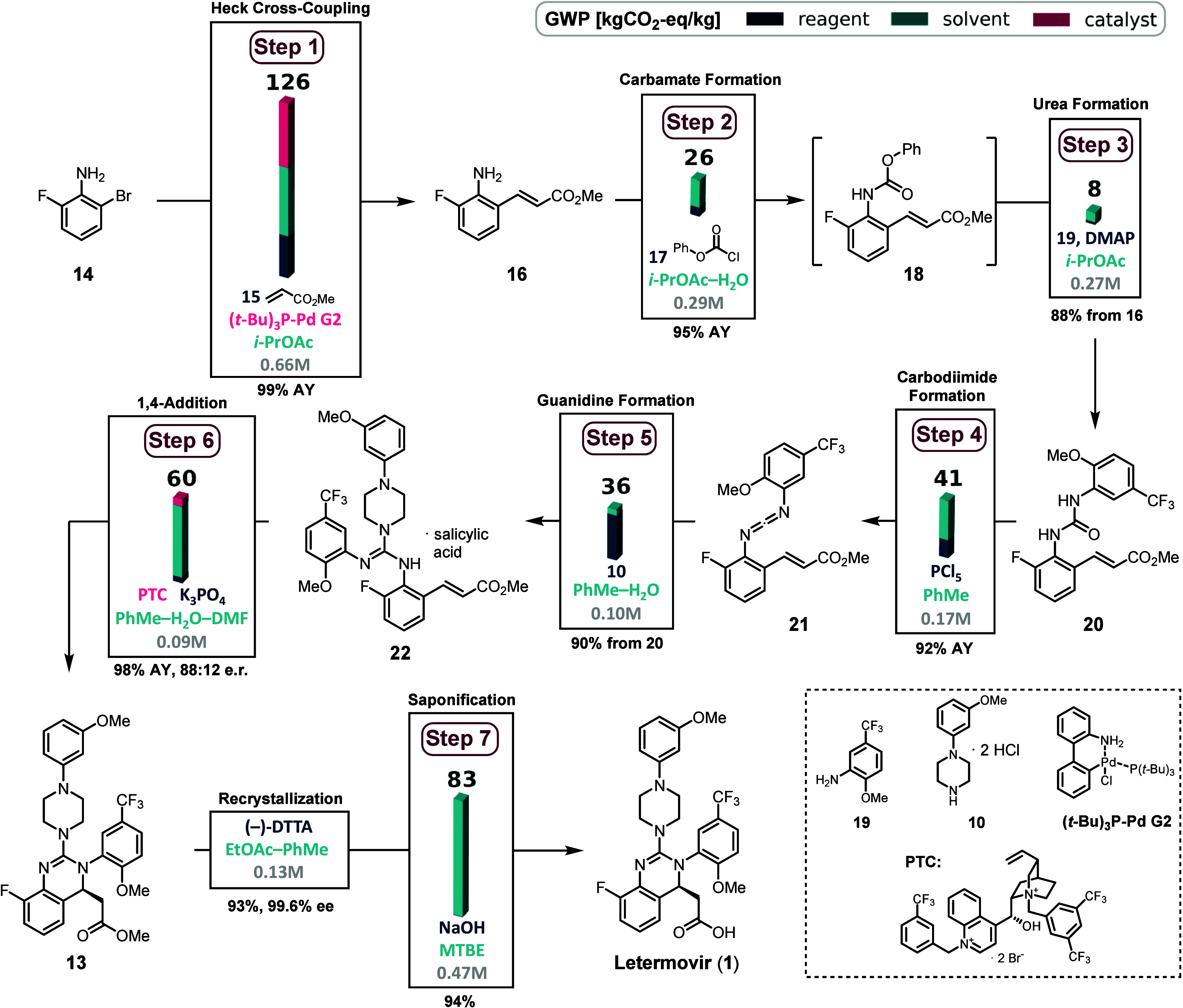
Reported Synthesis of Letermovir by Merck^a^ ^,^ ^28^

a Reagents and conditions: **Step 1**. **15** (2.0 equiv), c-Hex_2_NMe (1.2 equiv), (*t*-Bu)_3_P–Pd G2 (0.2 mol %), *i*-PrOAc, 80 °C, 99% AY; **Step 2**. **17** (1.25 equiv), Na_2_HPO_4_ (1.5 equiv), *i*-PrOAc–H_2_O, r.t. then 60 °C, ca. 95% AY; **Step 3**. **19** (1.1 equiv), DMAP (0.5 mol %), *i*-PrOAc, 80 °C, 93%; **Step 4**. PCl_5_ (1.2 equiv), 2-picoline (3.0 equiv), PhMe, 40 °C, 92% AY, **Step 5**. **10** (1.14 equiv), Et_3_N (2.4 equiv), salicylic acid (1.2 equiv), PhMe–H_2_O, 98%; **Step 6**. K_3_PO_4_ (1.5 equiv) then PTC (5.0 mol %), K_3_PO_4_ (1.5 equiv), glycolic acid (1.5 equiv), PhMe–H_2_O–DMF then (−)-DTTA (1.02 equiv), PhMe–EtOAc, 45 °C, 93%, 99.6% ee; **Step 7**. Na_2_HPO_4_ (2.5 equiv), MTBE–H_2_O then 1 M aq NaOH–MTBE, 60 °C, 94% AY.

### Merck Letermovir Synthesis

The reported synthesis relies on the quick access of key intermediate **22** from which the dihydroquinazoline core is accessed via an enantioselective intramolecular 1,4-addition catalyzed by cinchonidinium-derived phase-transfer catalyst **PTC**.^28^ For comparison purposes, the step count was aligned with the seven steps of our synthesis outlined in Scheme 2. Merck’s synthesis route commences from 2-bromo-6-fluoroaniline **14** with the introduction of methyl acrylate **15** via Pd-catalyzed Heck cross-coupling to form the corresponding cinnamate methyl ester **16**. A remarkably low catalyst loading of 0.2 mol % of (*t*-Bu)_3_P–Pd G2 affords product **16** in 99% assay yield (AY) and a GWP of 126 kgCO_2_-eq/kg. Subsequently, phenylchloro-formate **17** was used to form carbamate **18** which is then coupled with CF_3_–OMe-aniline **19** to obtain urea **20** in overall 88% yield with 26 and 8 kgCO_2_-eq/kg, respectively. Activation of **20** with PCl_5_ results in deoxygenation and formation of carbodiimide **21** (41 kgCO_2_-eq/kg). Nucleophilic addition of piperazine·2HCl **10** under basic conditions constructs the guanidine motif and after treatment with salicylic acid yields the key intermediate **22** with 36 kgCO_2_-eq/kg. After treatment of **22** with K_3_PO_4_, the free base was subjected to conjugate addition mediated by **PTC** under biphasic conditions to afford dihydroquinazoline **13** in 98% yield and 76% ee. Recrystallization of **13** with (−)-DTTA increased the enantiomeric excess up to 99.6% ee. Saponification concluded the synthesis of enantioenriched Letermovir **1**. Conjugate addition and saponification exhibited GWPs of 60 and 83 kgCO_2_-eq/kg, respectively. Approximately one year later, Merck reported improved reaction conditions for the enantioselective 1,4-addition leading to the key intermediate dihydroquinazoline **13**, noting its implementation in their commercial manufacturing process.^73^ The use of a newly developed hydrogen-bonding bistriflamide catalyst increased the performance of this step up to 95% yield and 96.7:3.3 er. Based on this adapted procedure Merck reported just recently a PMI of 193 kg/kg for the synthesis of Letermovir.^35^ However, it is important to note that, to the best of our knowledge, further details regarding the final manufacturing route and the data necessary for the LCA-based analysis are not publicly available. Therefore, the reported full synthesis route was used as the basis for comparison in this study.

### Comparative Analysis

The LCA results show that both syntheses have comparable CO_2_-eq emissions for producing 1 kg of Letermovir: Merck’s route yielded a GWP of 382 kgCO_2_-eq/kg_API_, and the optimized synthesis route of this work achieved a value of 369 kgCO_2_-eq/kg_API_ (Figures 3–5, Table 1). Step-by-step analysis of the route developed in this work shows that the increase of the GWP is mostly caused by Steps 6 and 7 ^74^ with an increase of 133 kgCO_2_-eq/kg_API_ and 83 kgCO_2_-eq/kg_API_, respectively (Figure 3B). In the preceding Steps 1 to 5, the increase of the GWP impact is fairly low with, for example, only 11 kgCO_2_-eq/kg_API_ in Steps 2 and 3. The breakdown in categories for all steps (Figures 3B and 4C) shows that solvents are primary contributors to the total impact, while reagents play a subordinate role. Analysis of Step 6 reveals that the catalyst used (**IDPi-1**) accounts for 51% of the GWP increase for this step alone and 24% of the cumulative GWP up to this point in the synthesis. This highlights the pronounced influence of complex catalysts on GWP (Figure 3E). The main cause is the low technology readiness level of the non-optimized **IDPi-1** synthesis.

**Figure 3:**
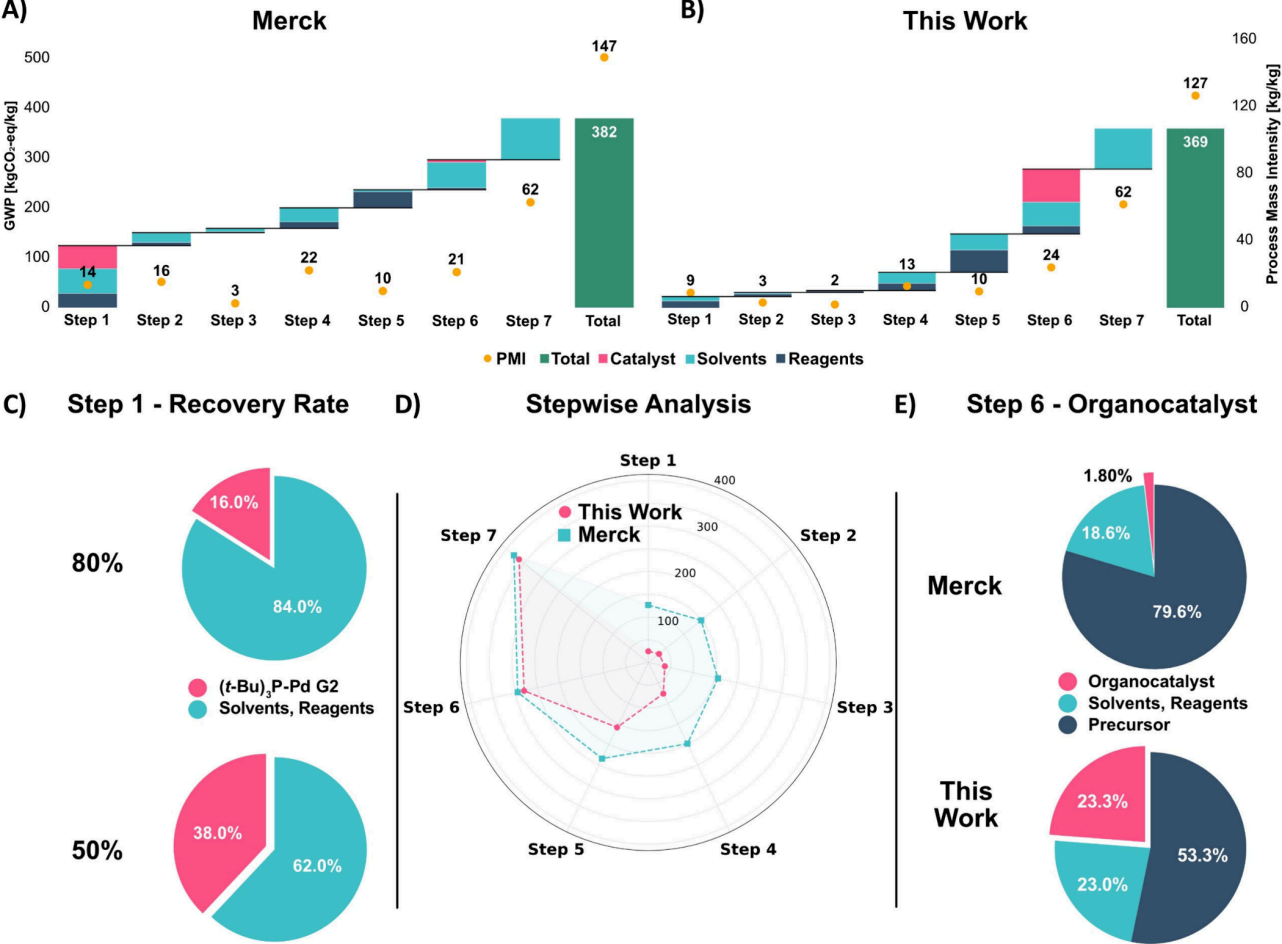
(A, B) Stepwise GWP impact analysis of both syntheses. (C) Sensitivity analysis of Pd catalyst impact regarding recovery rates (RRs) of 50% and 80% in Merck’s synthesis route. (D) Spider diagram for comparing visualization of GWP [kgCO_2_-eq/kg_API_] increases. (E) Identification of organocatalyst as a bottleneck in our synthesis route up until Step 6.

**Figure 4:**
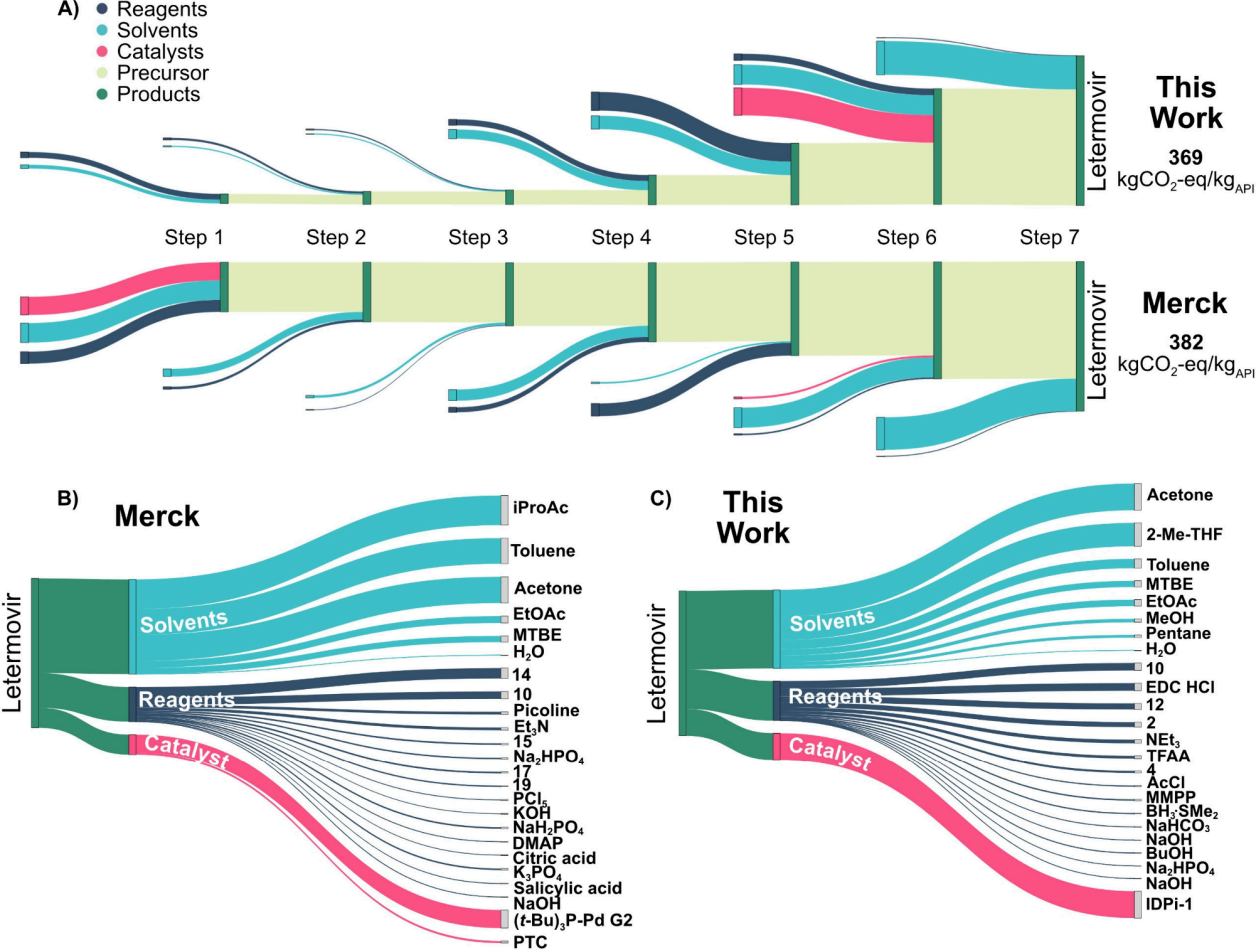
Flow analysis of the GWP for each individual step in our synthesis route and the synthesis route by Merck. Comparison of both routes in (A) and the detailed analysis (B, C) of contributions in both syntheses.

In particular, complex workup procedures involving large solvent volumes contribute significantly to the sustainability profile of the organocatalyst. Furthermore, the complex synthesis steps for this catalyst require noble metals and reagents such as palladium (11511 kgCO_2_-eq/kg), sodium hydride (8 kgCO_2_-eq/kg), *i*-PrOBpin (17 kgCO_2_-eq/kg), or *n*-BuLi (12 kgCO_2_-eq/kg).^51^ The manufacturing of all chemicals listed inherently involves emission-intensive processes. Although used in only 3 wt % (1 mol %), the **IDPi-1** catalyst plays a significant role in the overall GWP, with a value of 1461 kgCO_2_-eq/kg_catalyst_.

In Step 7, the solvents acetone and MTBE contribute 83 kgCO_2_-eq/kg_API_, 22% of the total GWP impact, due to their high usage at a 65:1 mass ratio relative to the precursor. Analysis of Merck’s synthesis reveals the largest increases of GWP in Steps 1 and 7,^65^ with contributions of 126 kgCO_2_-eq/kg_API_ and 83 kgCO_2_-eq/kg_API_, respectively. Similar to the LCA of the de novo synthesis, the largest impact in Merck’s route stems from solvent use, although the catalysts also contribute significantly. Specifically, Merck’s route employs a Pd-based catalyst ((*t*-Bu)_3_P–Pd G2) in the first synthesis step, which accounts for its significant influence on the GWP, with approximately 12%, or 46 kgCO_2_-eq/kg_API_ (Figure 4B). As an instructive example and because no data was reported, we conducted a sensitivity analysis, illustrated in Figure 3C. To that effect, we assessed the impact of the Pd catalyst by varying its theoretical recovery rate (RR). The pie charts in Figure 3C show the contributions of 38% and 16% of the Pd catalyst in Step 1, based on single use (50% RR) and reuse over three cycles (80% RR), respectively. Recovery rates of 80% or even 90% reduce the overall GWP of the synthesis by 35 kgCO_2_-eq/kg_API_ and 44 kgCO_2_-eq/kg_API_, respectively (see Table 1). The Pd catalyst from the Merck synthesis ((*t*-Bu)_3_P–Pd G2) exhibits a significantly higher GWP compared to the other reagents, with a value of 2398 kgCO_2_-eq/kg_Catalyst_, mostly attributed to palladium. In general, noble metals (i.e., palladium) require energy- and emission-intensive extraction and purification processes, accounting for the high environmental impact. Furthermore, in Step 1 the large volume of *i*-PrOAc contributes to the overall high GWP (126 kgCO_2_-eq/kg_API_) in this step. In contrast to Step 1, the subsequent Steps 2–6 in Merck’s route have a low effect on the synthesis’ GWP, largely attributed to reduced solvent use, selection of lower-impact solvents, and more sustainable, less complex reagents. Moreover, Step 6 employs biomass-derived cinchonidinium catalyst **PTC**, resulting in a markedly lower contribution to the GWP relative to the **IDPi-1** catalyst utilized in our synthesis shown in Scheme 2. This distinction is clearly depicted in Figure 3E (pink wedge). Although synthetic procedures for preparing cinchonidine (precursor for **PTC**) exist, extraction from tree bark remains the more practical source for this natural product. Therefore, instead of conducting a full synthetic LCA, we based the impact assessment on the extraction process. The corresponding life cycle data were derived from an inventory covering the complete natural production pathway, from tree cultivation to bark harvesting.^75^ This includes fertilizer usage, irrigation, harvesting, infrastructure, and transportation related to the tree, its bark, and cinchonidine. The extraction of cinchonidine from the bark and, separately, its conversion into the organocatalyst were calculated on the basis of experimental procedures from the literature.^28,76^ The overall calculated GWP impact (56 kgCO_2_-eq/kg_catalyst_) for this organocatalyst (**PTC**) is comparably low. Both routes incorporate the same saponification protocol in Step 7 that has a notable influence on the GWP. The use of acetone and MTBE as solvents for workup and purification contributes substantially to the GWP, with 83 kgCO_2_-eq/kg_API_. This step again underscores the critical influence of solvent choice and optimization on the sustainability of a synthesis.

The progression of the GWP impact across the synthesis steps is illustrated in the spider diagram (Figure 3D), showing pronounced increases in Steps 1 and 7 for Merck’s route. In contrast, our de novo synthesis (Scheme 2) maintains a low GWP impact through Steps 1–5, with increases observed in Steps 6 and 7. This underscores the benefits of a retrosynthetic strategy that places the high-impact steps of a synthesis as late as possible in the forward route.^55^

Comparative analysis of GWP impact and PMI in both syntheses is instructive, as consequences of discrepancies between the metrics emerge (Figure 3A and B). In Merck’s synthesis, PMI values remain low in Steps 1–6, with a sharp increase in Step 7 due to the large solvent volumes required for the saponification and purification. Interestingly, the PMI and GWP impact analyses for Merck’s synthesis diverge especially in Step 1. In contrast to the GWP impact (Figure 3A, Step 1), the PMI does not sufficiently capture the high impact of the (*t*-Bu)_3_P–Pd G2 catalyst. Additionally, PMI fails to cover the impact of varying RR of the catalysts, as the relative decrease in mass is negligible (Table 1). Conversely, LCA results, with its included GWP impacts, provide a more nuanced perspective (Table 1). In the de novo synthesis outlined in Scheme 2, particularly in Steps 1–5 and Step 7, with the latter adopted from the Merck route, PMIs reflect the influence on the gradual increase in GWP (Figure 3B). This behavior can be attributed to the solvent contribution, as the relative GWP impacts of the solvents exhibit lower fluctuations between different solvents. As such, better correlations among GWP impacts and PMIs is noted for steps that are solvent-dominated, i.e., the largest relative contribution to the metric. Although in Step 6 the PMI remains similar to that in preceding steps, the GWP markedly increases for this transformation. The reaction yield directly influences the PMI because of mass dependence. Analysis of the GWP impact remains more comprehensive, as it reflects individual contributions of reagents (i.e., **IDPi-1**) and solvents in an environmental context: In Step 6 PMI fails to capture the considerable carbon footprint linked to the catalyst (**IDPi-1**) relative to its low amount employed in this step. Consequently, yield losses in steps that employ high-impact reagents, such as catalysts, have a larger effect on the overall GWP of a synthetic route compared to yield losses in steps using lower-impact materials, such as solvents. This further underpins that synthetic routes should be designed to incorporate impact-heavy steps as late in the synthesis as possible. A key highlight of this study is the imperative to complement PMI metrics with LCA data to accurately assess the sustainability performance, including catalytic systems, of fine chemical and pharmaceutical synthesis.

The Sankey diagrams shown in Figure 4A enable direct comparing, contrasting, and benchmarking of CO_2_-eq flows for each step in both syntheses, categorized in reagents, solvents, catalysts, precursors, and products. Figure 4B and C show the contributions of each chemical and its category to the overall carbon footprints of the syntheses. In both syntheses the biggest contributors to the GWP are the solvents, followed by reagents, and catalysts. Acetone, in particular, has a significant impact as a single solvent in both syntheses but remains indispensable for the final purification of Letermovir in Step 7. Merck’s route mainly employs *i*-PrOAc and toluene, while the de novo synthesis route relies primarily on 2-Me-THF as solvent. Since LCA data were calculated for each building block, this approach delivers the means to compare the synthetic strategies based on their starting materials. Even though the absolute contributions of **14** and **15** in Merck’s synthesis and **2** in the de novo synthesis (Scheme 2) are subtle, these materials define the points of departure and foundations for the synthesis routes. Detailed analyses of this approach enable evaluation and consideration of building blocks and starting materials based on their origin, such as biomass-derived, renewable, or recyclable materials, to guide environmentally conscious synthetic design. Collectively, our results underscore the importance to develop renewable and sustainable reagents and building blocks to reduce the environmental impact of chemical synthesis.

**Table 1:** LCA Results in kgCO_2_-eq/kg_API_ for Recovery Rates (RR) of (*t*-Bu)_3_P–Pd G2 and Organocatalyst^a^

	Letermovir	RR 50%	RR 80%	RR 90%
GWP	Merck	382	350	342
	This work	369	323	311
PMI	Merck^77^	147	147	147
	This work	127	127	127

a Base case RR 50%.

The results collected in Table 1 demonstrate that the de novo synthesis meets the benchmark set by Merck’s published route in terms of both PMI and GWP. The latter is particularly significant, as it more accurately captures the catalyst’s influence on the overall environmental profile of the syntheses. The analysis on a mass intensity base (PMI) fails to grasp the influence of varying RR of the catalysts, while the GWP reflects the impact reduction of these bottlenecks that correlate with recovery rates (Table 1). In addition to the GWP, the LCA categories like ecosystem quality (EQ), natural resources (NR), and human health (HH) were assessed using the ReCiPe 2016 End points (E) method (Figure 5), broadening the scope of environmental impact analysis for both syntheses.^53^ The high performance of the de novo Letermovir synthesis is evident across all end point categories (EQ, NR, and HH), demonstrating improvements beyond the established high standards of the published Merck synthesis. Greater variations are observed for EQ and HH categories in both syntheses compared to NR, GWP, and PMI. The HH parameter between the two routes deviates by 33% (0.04 DALYs de novo route; 0.06 DALYs published route).

**Figure 5:**
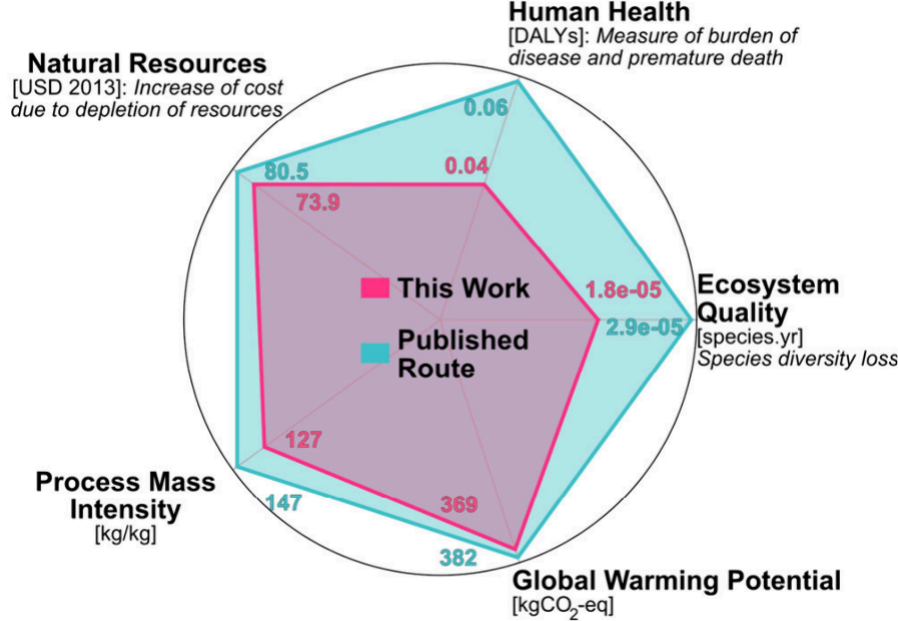
ReCiPe 2016 End points, IPCC 2021 GWP100a and PMI for both the synthesis from this work and the published route. DALYs = Disability Adjusted Life Years; species.yr = number of species lost over 1 year; USD = resource cost increase.

A similar trend is observed for the EQ, with a difference of 38% (1.8 × 10^–5^ species.yr de novo route; 2.9 × 10^–5^ species.yr published route). In the end point analysis of the depletion of natural resources (NR) the routes show similar effects with deviations of 8% (73.9 USD de novo route; 80.5 USD published route). The remaining PMI and GWP show differences in the outcomes of about 15% and 3%, respectively. Detailed analysis reveals that in all three end points (EQ, NR, and HH) the palladium used in the first step of Merck’s published route accounts for the majority of the increase in these categories, mainly due to its extraction and energy-intensive production. Additionally, large amounts of solvents like acetone in Step 7 significantly increase EQ, NR, and HH effects in both syntheses because of their production processes and associated water toxicity. In contrast, reagents, excluding catalysts, have substantially lower influence on these categories in both routes.

## Conclusion

We have developed an enhanced closed-loop LCA-guided approach for the de novo synthesis of Letermovir that enables comprehensive sustainability evaluation. As such, we directly compared and contrasted a de novo route to a published Merck synthesis across multiple LCA categories (GWP, EQ, NR, HH) and widely used PMI values. This comprehensive analysis necessitated generation of complete data sets by calculating missing chemical data using retrosynthetic reconstruction grounded in experimental literature. The de novo synthesis features a number of innovative steps. The use of an anthranilic acid as starting material enables the introduction of the C(4) acetic acid side chain via condensation chemistry. Oxidation-state management was effected by a Pummerer rearrangement to ultimately provide key intermediate guanidine-derived hemiaminal **11**. Moreover, we describe the first enantioselective Mukaiyama–Mannich addition of silyl ketene acetal catalyzed by a chiral P-based Brønsted acid (IDPi). Our LCA-guided optimized synthesis route demonstrates a highly competitive sustainability profile based on the impact categories analyzed, namely, PMI (127 kg/kg), GWP (369 kgCO_2_-eq/kg_API_), NR (73.9 USD), HH (0.04 DALYs), and EQ (1.8 × 10^–5^ species.yr). We quantitatively assessed the influence of various catalysts, including organocatalysts (**IDPi-1**, **PTC**) and a transition metal catalyst ((*t*-Bu)_3_P–Pd G2), in the two routes under scrutiny, and we evaluated the effects of recovery rates on the overall synthesis sustainability profile. Beyond expected hotspots such as solvents, catalysts, and yields the analysis revealed less obvious sustainability bottlenecks. The detailed accounting of all the chemicals and their environmental impacts facilitates the identification of improvement opportunities, as demonstrated by reagent substitution such as LiAlH_4_ and *m*CPBA by BH_3_·SMe_2_ and MMPP, respectively.

This work highlights how LCA insights inform decision-making beyond conventional assumptions and substantively augment the use of simple process-level metrics. The integration of LCA into synthetic design in this work enables more sustainable decision-making and inspires innovation for the development of environmentally conscious chemical synthesis. The implementation of the delineated framework demonstrates that well-designed life cycle assessments are germane to complex, multistep syntheses of fine chemicals or pharmaceuticals. The tool enables reliable and comparable evaluations without omission of critical components or reliance on commonly accepted assumptions to bridge current data gaps. Most importantly, this provides a novel critical view on how current syntheses are planned and enables a sustainable way forward for the field of chemical synthesis.

## Supplementary Material





## Notes

Experimental procedures and characterization data for all new compounds (PDF)
Evaluation of the data, based on LCA method (XLSX)
