# Supplementary material for: Integrated Life Cycle Assessment Guides Sustainability in Synthesis: Antiviral Letermovir as a Case Study
Source: J Am Chem Soc. 2025 Oct 27;147(44):40944–57. doi: 10.1021/jacs.5c14470 (PMC12593353; doi:10.1021/jacs.5c14470)
Supplement: Supplementary file 1 [file ja5c14470_si_001.pdf]

Supporting Information

Integrated Life Cycle Assessment Guides Sustainability in Synthesis: Antiviral Letermovir as a Case Study

Sander Folkerts[1,3‡], Maximilian G. Hoepfner[2,3‡], Gonzalo Guillén-Gosálbez[2,3*], Javier Pérez-Ramírez[2,3*], Erick M. Carreira[1,3*]

Contents

1. Life Cycle Assessment (LCA) 3
2. General Information for Calculation of Life Cycle Inventory (LCI) Data 12
3. Chemistry 19
4. Optimization Mukaiyama–Mannich Addition 21
5. Mechanistic Studies 22
6. Experimental Section of Letermovir Route 36
7. NMR Spectra 55

1. Life Cycle Assessment (LCA)

Introduction

This document outlines the assumptions made during the Life Cycle Assessment (LCA) study titled "Integrated Life Cycle Assessment Guides Sustainability in Synthesis: Antiviral Letermovir as a Case Study". These assumptions are critical to the interpretation and reproducibility of the results. The goal of this study is to identify life cycle impacts for the antiviral drug Letermovir (IUPAC: {(4S)-8-Fluoro-2-[4-(3-methoxyphenyl)-1-piperazinyl]-3-[2-methoxy-5-(trifluoromethyl)phenyl]-3,4-dihydro-4-quinazolinyl} acetic acid)

System Boundaries

Temporal Boundaries: The study considers a time frame for 100 years in terms of GWP. The general time boundary for the production is assumed to be momentary, since no further information on the production routes are given, e.g., like production time horizon of the here so-called product Letermovir.

Geographical Boundaries: Activities were selected from locations RER, ROW and GLO, depending on availability of data in ecoinvent. If possible, market activities were chosen, if not production activities were chosen.

Life Cycle Phases: The LCA includes starting materials, reagents, solvents and catalyst. The LCA is performed in accordance with ISO 14044 in terms of a cradle-to-gate analysis.

More detailed information for individual assumptions can be found in the calculation excel sheet.

Functional Unit

Definition: The functional unit for this study is defined as 1 kg of the final antiviral drug Letermovir.

Data Sources and Quality

Primary Data: Data for raw materials and solvents were obtained from ecoinvent (v3.9.1, v3.10. and v3.11.). Reaction paths were determined using literature reported experimental data.

Data Gaps: In cases where data were unavailable in the ecoinvent database, especially if starting materials were not available, literature data was employed in order to trace back to raw materials which are present in ecoinvent. Occasionally, the used publications were not able to provide all amounts of solvents and reagents. Stoichiometric matching values were assumed to determine usage of the regencies in case no information was given. Moreover, the usage of solvents was assumed to be 1 kg for each step or 1 mol/L if not specified, due to lack of data. Individual and local assumptions can be found in the excel file. If no sufficient pathways were available to model data gaps, economic allocation was utilized (e.g., 2-Me-THF).

Impact Assessment Methodology

Chosen LCA Method: The impact assessment was conducted using the IPCC 2021 GWP 100a method for global warming potential and ReCiPe 2016 v1.03 for Endpoints (E) analysis. Brightway and the Activity-Browser in Python 3.12.9 were used for the computation of LCA data. Microsoft Excel Version 2108 was used for impact calculation of reaction pathways.

Allocation Procedures

Co-Product Allocation: For processes generating multiple products, allocation was based on mass stoichiometry. Usually, the identification of the main product is easy to determine. By-products were neglected and not assumed in the calculation.

Recycling and Waste Management: Assumptions regarding recycling and waste management is limited to the recovery of the catalyst, assuming various recovery rates. Sensitivity analysis for the recovery of solvents can be found in Table S2.

Limitations

Scope Limitations: This study does not consider the cradle-to-grave impacts, which are the disposal of the examined drug or all by-products which are produced during the synthesis. Moreover, neither the infrastructure nor the machineries for the production were directly included in the analysis. Solely, the data from the raw materials, reagents, solvents and catalysts were employed in the LCA.

Table S1: Life cycle inventory for the selected organocatalyst based on the tree Cinchona as biomass.[1] Loc = location.

Activity Name	Reference Product	From loc.	Database Origin	To loc.	To database	Flow type	Amount	Unit
Organocat (precursor)	organocat	GLO	Organocat	GLO	Organocat	production	1	kg
nutrient supply from manure, solid, cattle	organic phosphorus fertiliser, as P2O5	GLO	ecoinvent-3.10-cutoff	GLO	Organocat	technosphere	4442	kg
market for urea	urea	RoW	ecoinvent-3.10-cutoff	GLO	Organocat	technosphere	9225	kg
market for potassium chloride	potassium chloride	RoW	ecoinvent-3.10-cutoff	GLO	Organocat	technosphere	2170	kg
market for magnesium sulfate	magnesium sulfate	GLO	ecoinvent-3.10-cutoff	GLO	Organocat	technosphere	1521	kg
tap water production, direct filtration treatment	tap water	RER	ecoinvent-3.10-cutoff	GLO	Organocat	technosphere	173580	kg
market for combine harvesting	combine harvesting	GLO	ecoinvent-3.10-cutoff	GLO	Organocat	technosphere	1	hectare
lime to generic market for soil pH raising agent	soil pH raising agent, as CaCO3	GLO	ecoinvent-3.10-cutoff	GLO	Organocat	technosphere	1000	kg
transport, freight, sea, container ship with reefer, cooling	transport, freight, sea, container ship with reefer, cooling	GLO	ecoinvent-3.10-cutoff	GLO	Organocat	technosphere	9800	ton km

The phase-transfer-catalyst (PTC) employed in Merck's Letermovir synthesis in the enantioselective intramolecular 1,4-addition is derived from cinchonidinium. To enable a cradle-to-gate LCA, the inventory for the precursor of the catalyst was derived from the according biomass from the tree Cinchona. Mass flows for the production (e.g., planting, fertilizer, harvesting) are included according to Wasis et al., *Discussion material for Tropical Forest Nutrition Course* **2020**. A function unit of 1 kg of the precursor of the catalyst is assumed. Impact assessment was performed in brightway25. Final synthesis step to the PTC can be found in the excel file.

Table S2: Sensitivity analysis of solvent usage in both this work and the synthesis route by Merck. 100 % denotes the base case with solvent amounts scaled from lab scale to match the synthesis in terms of the selected FU (1 kg) of Letermovir.

**Solvent Usage** [%]	**This Work** [kgCO_2-eq/kg]	**Merck** [kgCO_2-eq/kg]
Very high recovery (25 %)	268	288
High recovery (50 %)	302	319
**Base Case (100 %)**	**369**	**382**
Low recovery (150%)	435	445
Very low recovery (200%)	502	507

To enable a more sensitive overview of the impacts, generated by solvents, a sensitivity analysis was performed. For this matter, the employed mass of each individual solvent was increased and decreased. Impact assessment based on IPCC 2021 GWP 100 was performed for each case. High recovery denotes lower mass of solvent required, and low recovery denotes high usage of solvents respectively.

Recovery Rate of Catalyst

In order to adjust the life cycle impact based on the different recovery rates *r* of the catalysts the number of possible cycles *n*, in which the catalyst can be used, need to be calculated. The cycles were calculated based on the exponential decay model as in the equation below, including the threshold activity of the catalyst R_n:[2]

$$R_n = (1 - r)^n$$

After reformulation this yields:

$$n = \frac{\ln(R_n)}{\ln(1 - r)}$$

The life cycle impact was subsequently adjusted based on the selected recovery rate of the catalyst. This type of adjustment does not alter the employed mass of the catalyst in the synthesis.

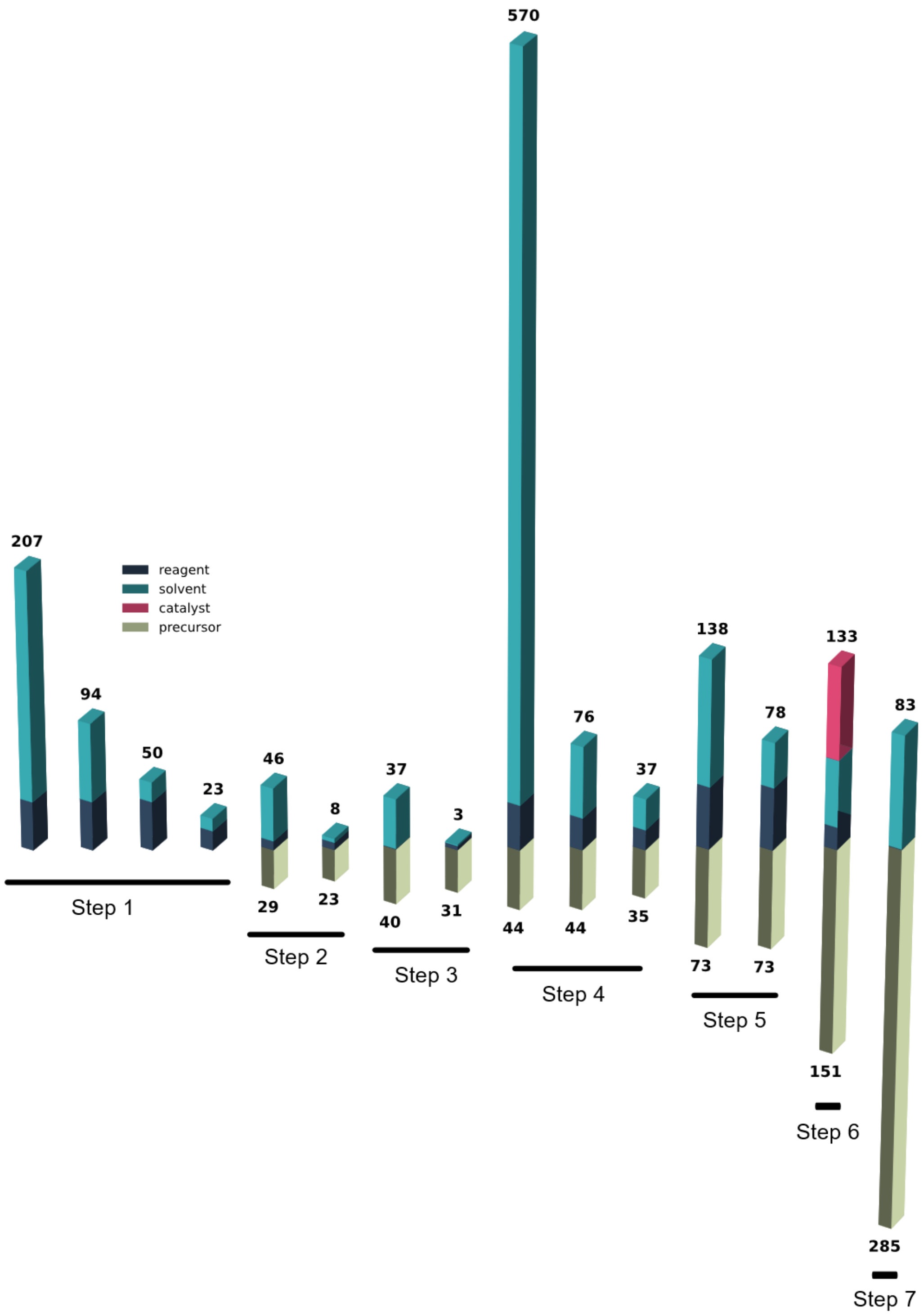

Figure S1: Impact contribution of reagents, solvent, catalyst and precursor by optimizing synthesis conditions for the Letermovir synthesis presented in this work. Precursor includes yield changes in later steps.

Variations in yield from step 2 onwards not only affect the quantities of reagents, solvents, and catalysts required for the corresponding individual step, but also impact the amount of precursor needed from preceding steps. As a result, any change in yield propagates upstream through the synthesis, altering the required amounts of reagents, solvents, and catalysts throughout the synthetic sequence. This interdependency has been carefully accounted for in all material and energy balance calculations presented herein.

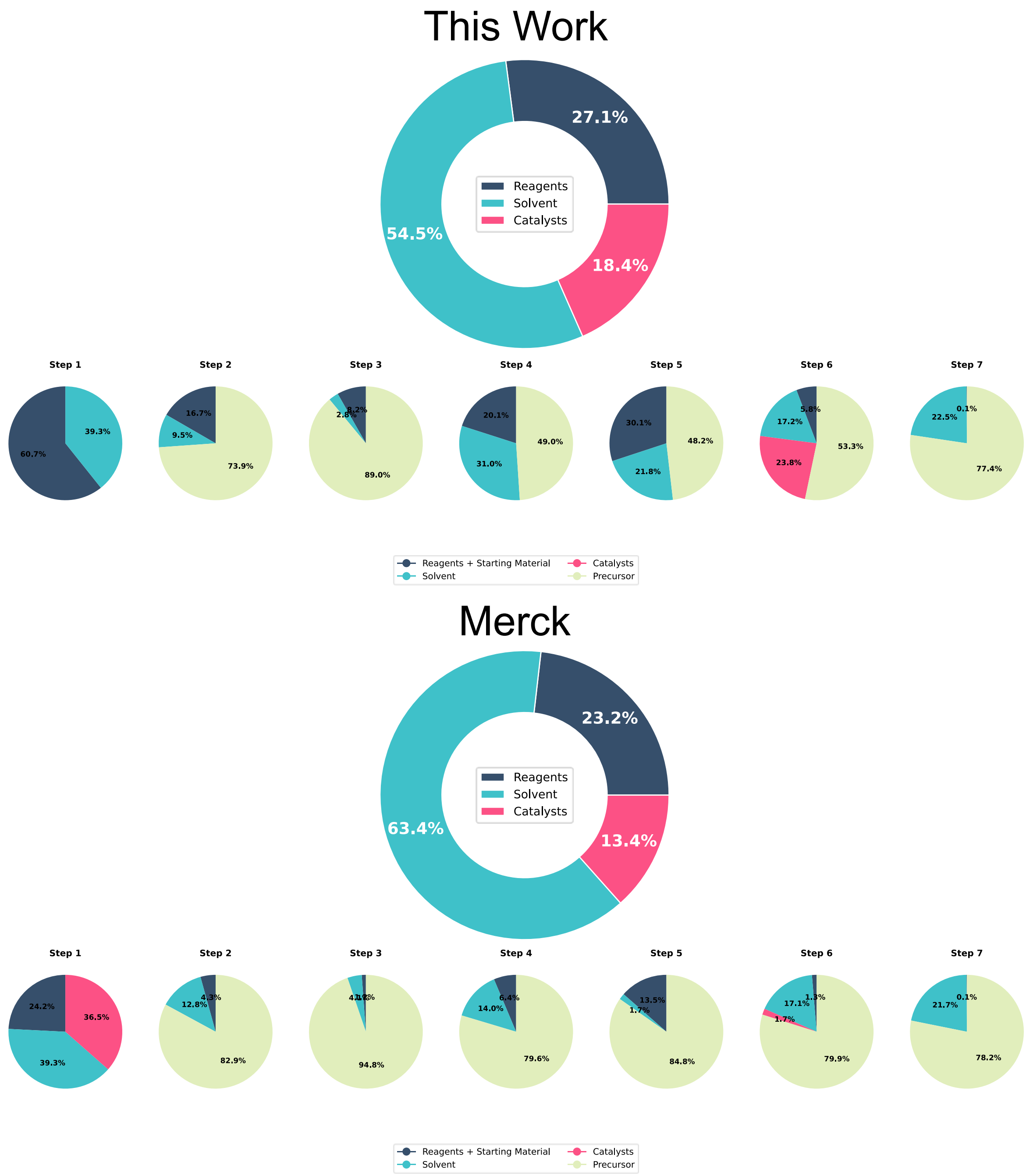

Figure S2: Step-by-step contribution analysis of both this work and the synthesis published by Merck.

To supplement the step-by-step analysis in this work, all contributions for each individual step are visualized. The total contribution of both synthesis (ring chart) are the weighted contributions by each step, based on the impact of the according step.

ReCiPe Single Score Calculation

In order to set the midpoint and endpoint analysis into perspective, a single-score evaluation was performed. This procedure involves a weighting and normalization of the endpoint values, based on provided reference values.[3]

Table S3: Weighting Factors for ReCiPe Methods.

Perspective:	Ecosystems - Weights	Human health - Weights	Resources - Weights	Total
Average	400	400	200	1000
Individualist	250	550	200	1000
Hierarchist	400	300	300	1000
Egalitarian	500	300	200	1000

Table S4: Normalization Factors for ReCiPe (E) methods.

Endpoints	ReCiPe Midpoint (E) World – Normalization
Ecosystems	2.48E-03
Human health	2.42E-02
Resources	2.45E+02

Table S5: ReCiPe single score results for both synthesis routes.

	Weighting Egalitarian		Weighting Average	
**Endpoints**	**This Work** [Pt]	**Merck** [Pt]	**This Work** [Pt]	**Merck** [Pt]
Ecosystems	3.684	5.858	2.947	4.686
Human health	452.2	797.3	602.9	1063.0
Resources	60.34	65.72	60.34	65.72
Total	516.2	868.8	666.2	1130

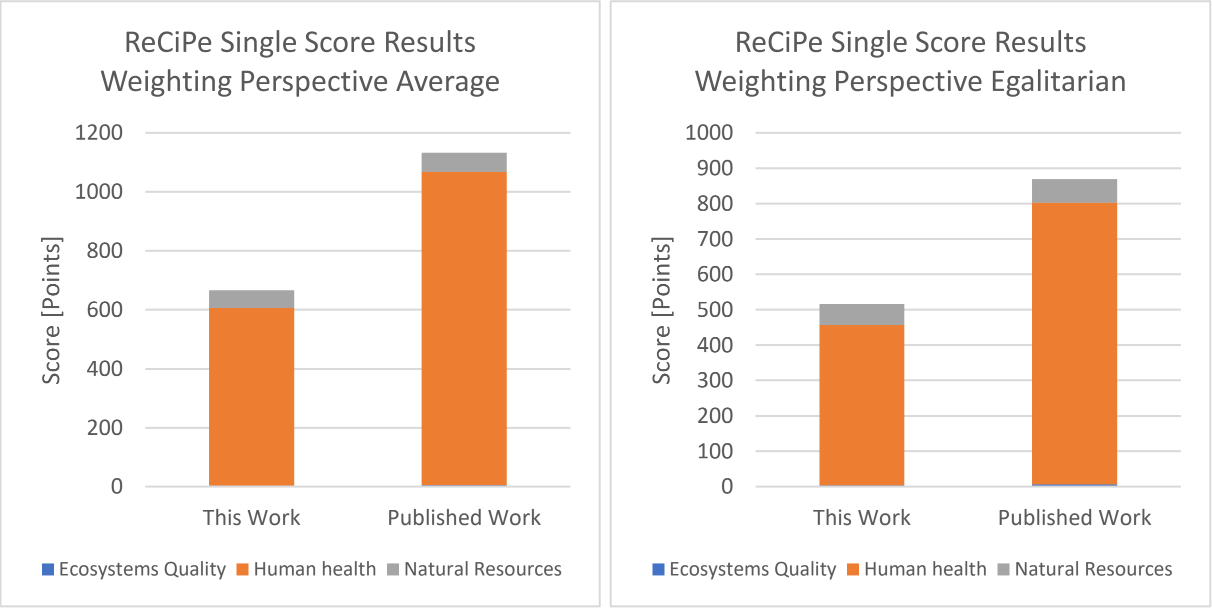

Figure S3: Comparison of the single-score values of both syntheses.

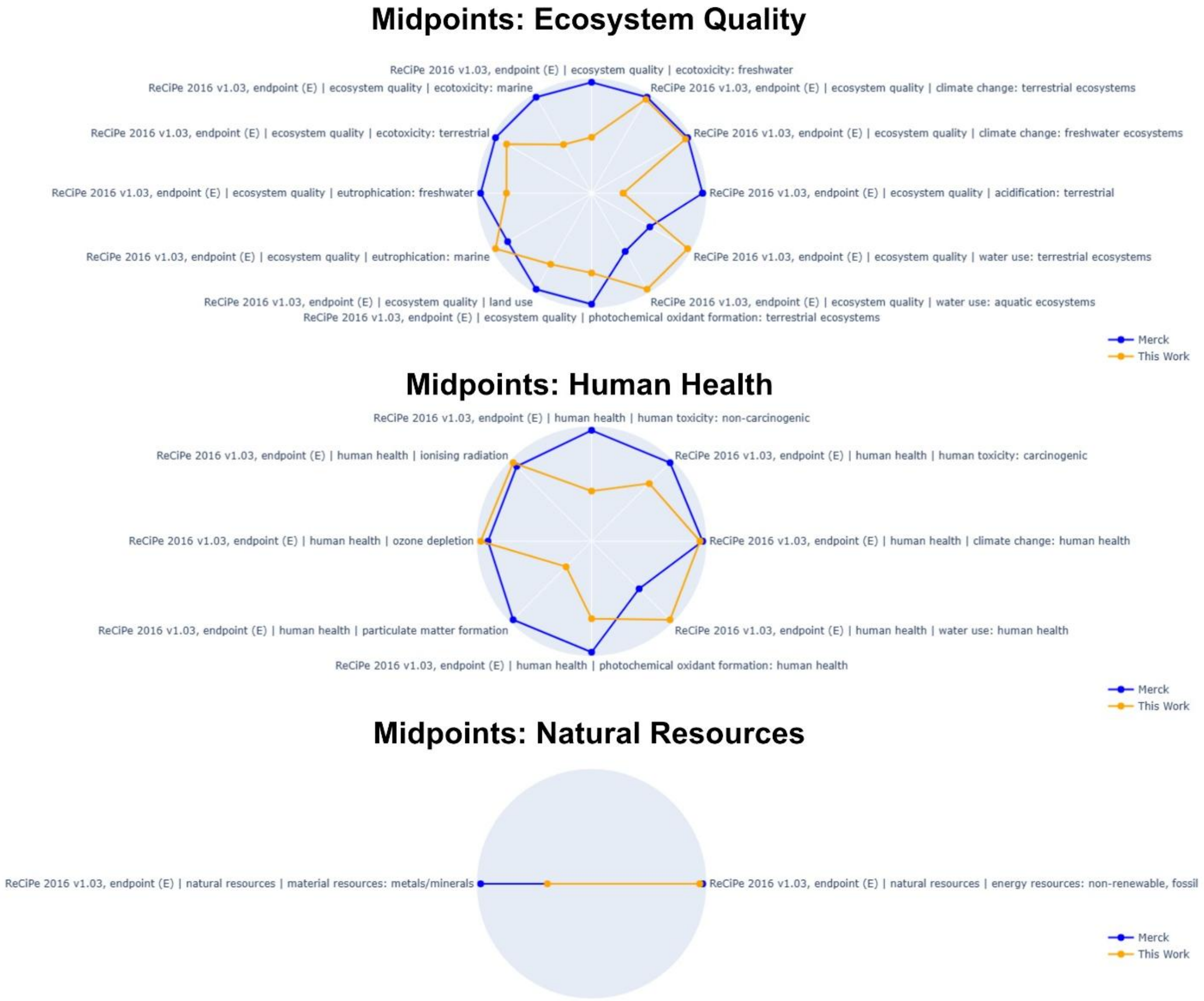

Figure S4: ReCiPe 2016 v1.03 Midpoint analysis. Blue, Merck's synthesis route, orange, synthesis route of this work.

Table S6: ReCiPe 2016 v1.03 Endpoints (E), midpoint analysis of both investigated synthesis routes.

Methode	Merck's Synthesis Route	This Work	Unit
ecosystem quality | acidification: terrestrial	1.6462E-06	4.70261E-07	species.yr
ecosystem quality | climate change: freshwater ecosystems	2.22049E-10	2.17269E-10	species.yr
ecosystem quality | climate change: terrestrial ecosystems	8.13876E-06	7.9636E-06	species.yr
ecosystem quality | ecotoxicity: freshwater	2.64227E-08	1.32991E-08	species.yr
ecosystem quality | ecotoxicity: marine	1.86367E-05	9.48514E-06	species.yr
ecosystem quality | ecotoxicity: terrestrial	4.29328E-08	3.84124E-08	species.yr
ecosystem quality | eutrophication: freshwater	8.66415E-08	6.64245E-08	species.yr
ecosystem quality | eutrophication: marine	5.84129E-11	5.82212E-11	species.yr
ecosystem quality | land use	7.72631E-08	5.70063E-08	species.yr
ecosystem quality | photochemical oxidant formation: terrestrial ecosystems	1.9455E-07	1.37856E-07	species.yr
ecosystem quality | water use: aquatic ecosystems	2.1143E-12	3.65717E-12	species.yr
ecosystem quality | water use: terrestrial ecosystems	4.72568E-08	8.17415E-08	species.yr
human health | climate change: human health	0.0040698	0.003982203	DALYs
human health | human toxicity: carcinogenic	0.018573524	0.014052644	DALYs
human health | human toxicity: non-carcinogenic	0.039366191	0.017891973	DALYs
human health | ionising radiation	4.26494E-07	4.3943E-07	DALYs
human health | ozone depletion	4.86919E-07	3.70119E-07	DALYs
human health | particulate matter formation	0.001475136	0.000480667	DALYs
human health | photochemical oxidant formation: human health	1.22083E-06	8.41307E-07	DALYs
human health | water use: human health	7.77112E-06	1.34419E-05	DALYs
natural resources | energy resources: non-renewable, fossil	72.69223739	70.68496154	USD 2013
natural resources | material resources: metals/minerals	9.12402641	2.893969243	USD 2013

2. General Information for Calculation of Life Cycle Inventory (LCI) Data

Synthetic routes were identified by iterative retrosynthesis and forward synthesis formulation simultaneously with life cycle database comparison. Further information for the calculation of the life cycle inventories (LCIs) for each chemical are to be found in the excel sheet as notes.

14 synthesis route for LCI:

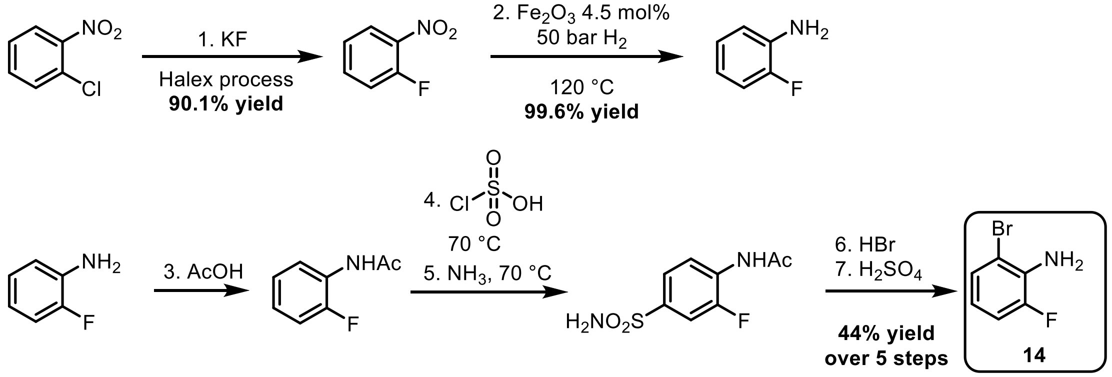

Step 1. Halex process:
Siegemund, G., Schwertfeger, W., Feiring, A., Smart, B., Behr, F., Vogel, H. and McKusick, B. (2000). Fluorine Compounds, Organic. In Ullmann's Encyclopedia of Industrial Chemistry, (Ed.). DOI: 10.1002/14356007.a11_349

Step 2. Wanhua Chemical Group Co Ltd - CN115322123, 2022, A.

Step 3-7. Shandong Chengchuang Blue Sea Pharmaceutical Technology Co ltd - CN115784896A, 2022.

2 synthesis route for LCI:

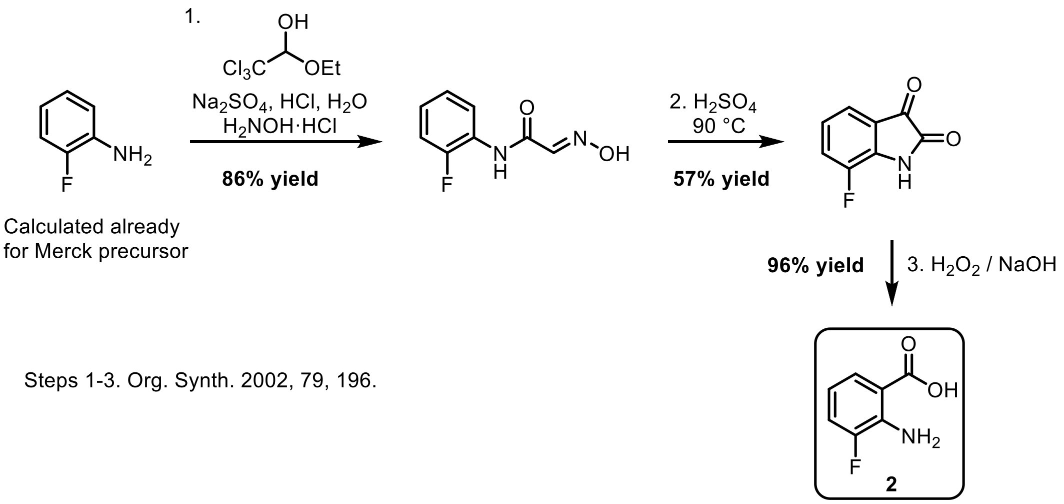

Steps 1-3. Org. Synth. 2002, 79, 196.

10 synthesis route for LCI:

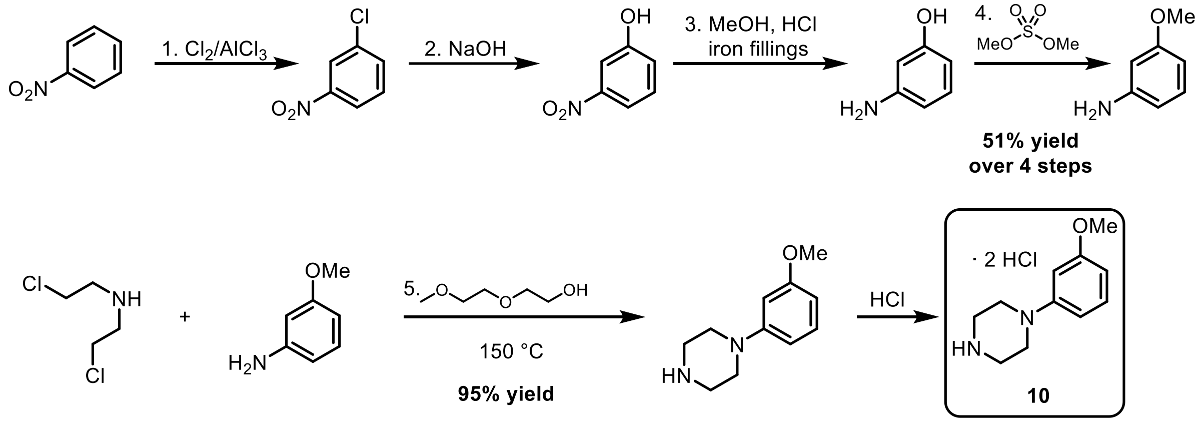

Step 1. Bromine Compounds Ltd. - WO9919275 A1, 1999.

Step 2. Rhodia Chimie SAS - US20080045756A1, 2007.

Step 3. J. Chem. Soc., Trans., 1925,127, 494–498.

Step 4. J. Org. Chem. 1957, 22, 3, 333–334.

Step 5. Tetrahedron Letters 2005, 46, 7921–7922.

dichlorodiethylamine: IG Farbenindustrie AG - CH195842A, 1938.
IG Farbenindustrie AG - FR802416A, 1936.
IG Farbenindustrie AG - US2163181A, 1936.

19 and 4 synthesis routes for LCIs:

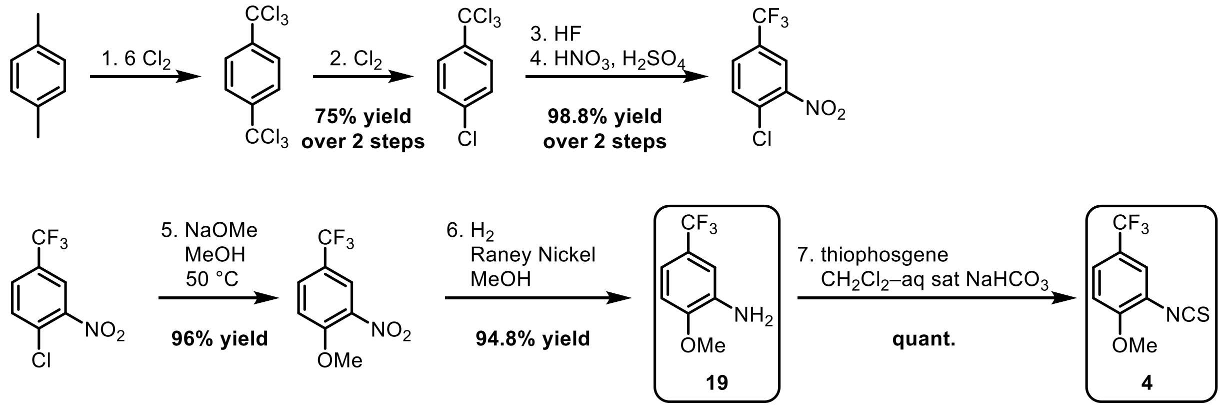

Steps 1-2.Corteva Agriscience LLC - EP0534412A2, 1992.

Step 3. SRF Limited - WO2015125155A1, 2015.

Step 4. SINOCHEM SAFETY SCIENCE RESEARCH SHENYANG - CN113121356, 2021, A.

Step 5 and 6. EISNITE CHENGDU BIOPHARMACEUTICAL - CN114163316, 2022, A.

Step 7. BAYER PHARMA - WO2016/166186, 2016, A1.

MMPP synthesis route for LCI:

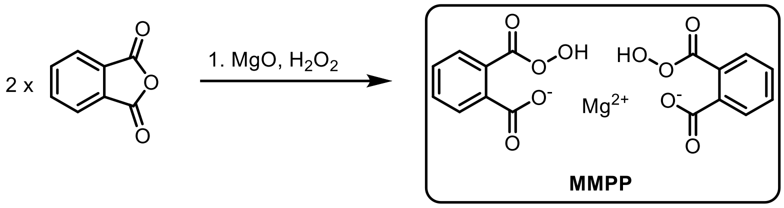

Step 1. Interox Chemicals Ltd - EP0027693A1, 1980.

*m*CPBA synthesis route for LCI:

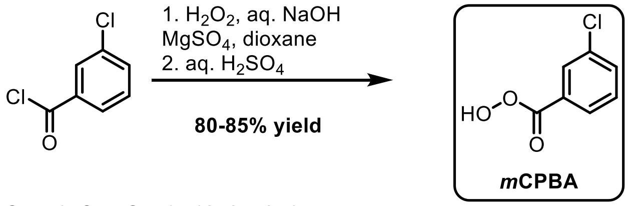

Step 1. Org. Synth. 1970, 50, 15.

EDC·HCl synthesis route for LCI:

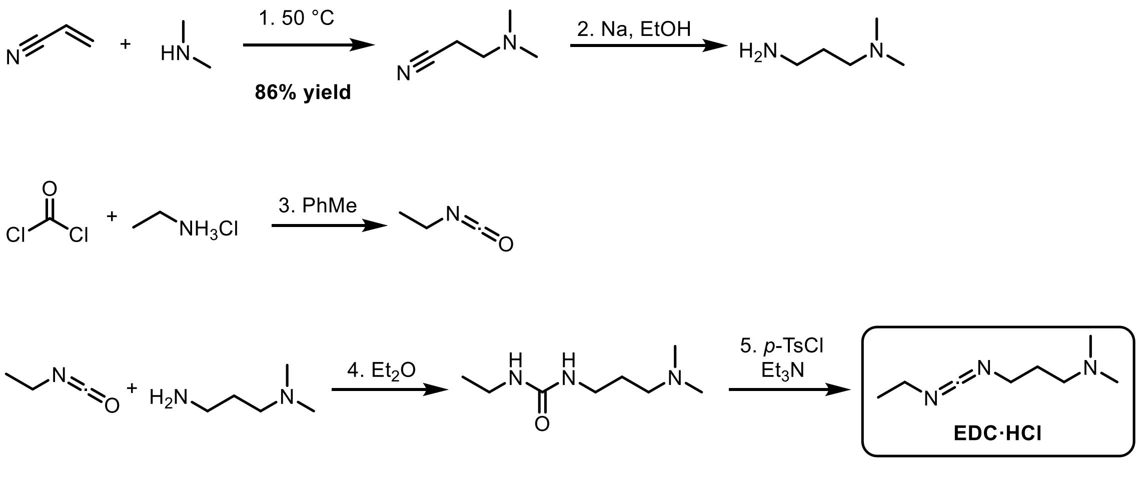

Steps 1 and 2. Vogel's Textbook of Practical Organic Chemistry
Longman Inc: New York, USA 1978, p. 744–798.
J. Phys. Org. Chem. 2005, 18, 880–885.

Step 3. Wanhua Chemical Group Co Ltd - CN107935889A, 2017.

Step 4 and 5. J. Org. Chem. 1961, 26, 7, 2525–2528.
SHANDONG JINCHENG KERUI CHEMICAL CO Ltd - CN109369458A, 2018.

n-BuLi synthesis route for LCI:

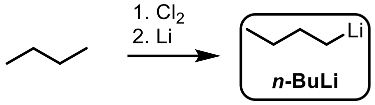

Steps 1 and 2. FMC Corp - US5523447A, 1995.
J. Am. Chem. Soc. 1949, 71, 4, 1499–1500.

NaH synthesis route for LCI:

$$2\ Na + H_2 \xrightarrow{1.} 2\ NaH$$

Step 1. ALUMINAL OBERFLACHENTECHNIK GMBH & CO. KG - US20040258613A1, 2004.

Phenyl chloroformate synthesis route for LCI:

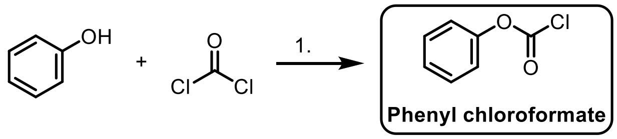

Step 1. Us Ind Chemicals Inc - US2496091A, 1947.
Hoechst AG - DE2131555C3, 1971.

(t-Bu)$_3$P-Pd G2 synthesis route for LCI:

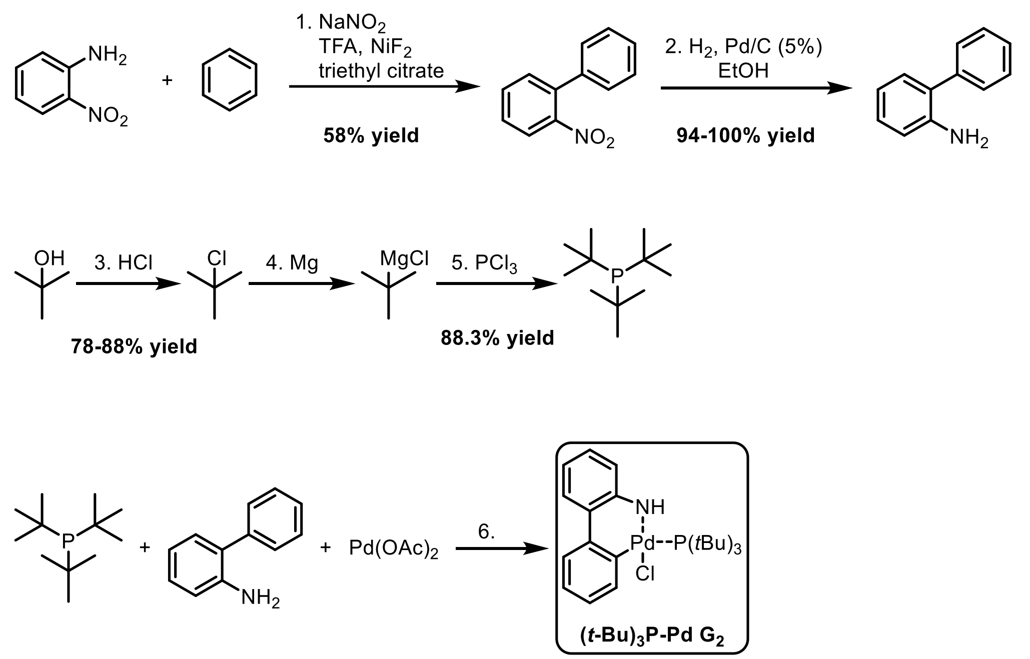

Step 1. Xuzhou B&c Chemical Co ltd - CN108069861B, 2018.

Step 2. Org. Synth. 1966, 46, 85.

Step 3. Org. Synth. 1928, 8, 50.

Step 4. DOW TORAY - WO2006/38707, 2006, A2.

Step 5. SALTIGO - US2003/229240, 2003, A1.

Step 6. Chem. Sci. 2013, 4, 916-920.

BINOL synthesis route for LCI:

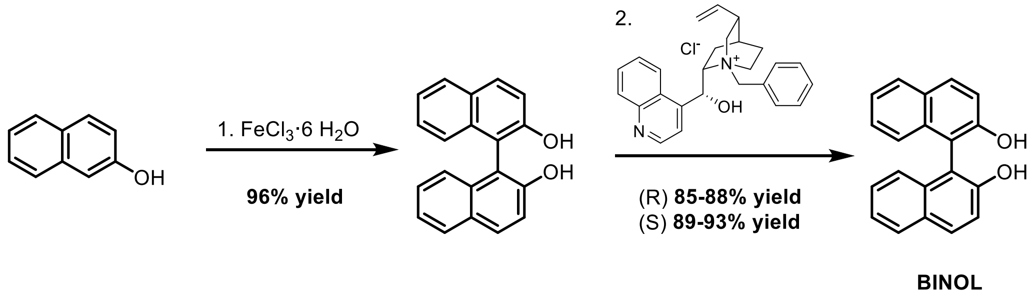

BINOL
(R) 99%ee
(S) 99%ee

Step 1. RSC Adv. 2016, 6, 39024-39038.

Step 2.Org. Synth. 1999, 76, 1.

2-bromobenzofuran synthesis route for LCI:

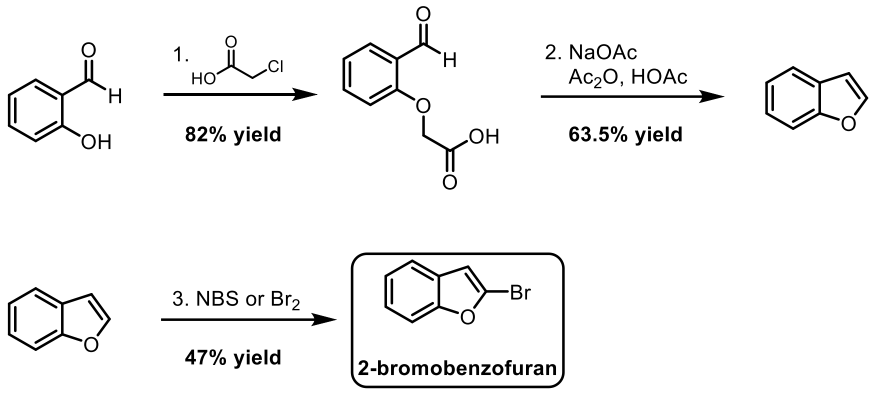

Steps 1 and 2. Org. Synth. 1966, 46, 28.

Step 3. SHANGHAI FEIKAI MATERIAL TECH CO LTD; SHANGHAI CHUANQIN NEW MAT CO LTD - CN115873044A, 2023.
AOLAIDE SHANGHAI OPTOELECTRONIC MATERIALS TECH CO LTD - CN116199705A, 2023.

Benzyl bromide building block synthesis routes for LCI:

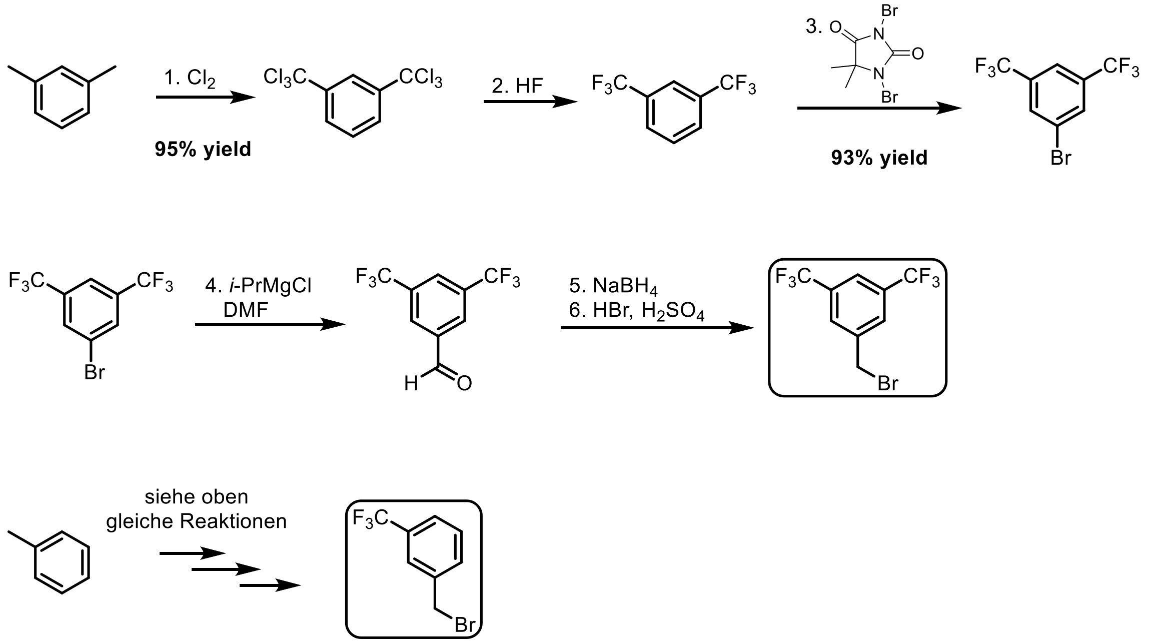

Step 1. SHANGHAI FANGLUN NEW MATERIAL TECHNOLOGY Co Ltd - CN104447188B, 2016.

Step 2. IG FARBENINDUSTRIE HISTORIC - DE575593C, 1931.

Step 3. Merck and Co Inc - US6350915B1, 2002.

Steps 4 to 6. Org. Process Res. Dev. 2021, 25, 12, 2795–2805.

S11 LCI synthesis route:

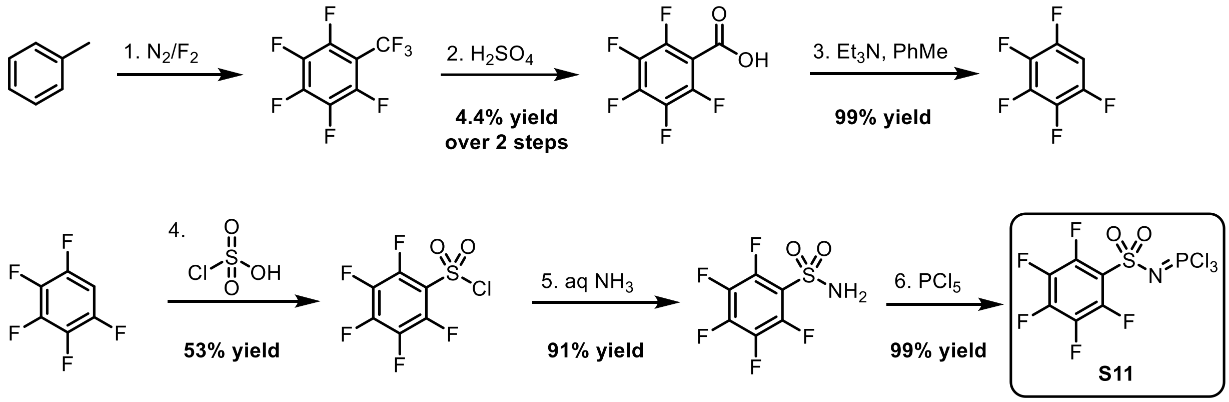

Steps 1 and 2. Nature 1959, 183, 588–589.

Step 3. SHANGHAI CHEMSPEC COPRORATION - CN106810423, 2017, A.

Step 4. Phosphorus, Sulfur, and Silicon and the Related Elements 1995, 107, 27-31.

Step 5. FUJIFILM - JP2015/163674A, 2015.

Step 6. Angew. Chem. Int. Ed. 2018, 57, 12162-12166.

3. Chemistry

General Information

All reactions were carried out under ambient atmosphere and at room temperature unless otherwise noted. Reactions sensitive to oxygen and water were carried out under nitrogen atmosphere and in glassware dried with a heatgun (ca. 650 °C) under high vacuum (<1 mbar). Syringes were purged trice with nitrogen prior to use for transferring of anhydrous solvents or sensitive reagents. Concentration under reduced pressure (*in vacuo*) was performed at 40 °C with a rotary evaporator at the corresponding pressure regarding the individual solvents unless otherwise noted. The yields refer to purified products, unless otherwise mentioned.

Reagents and Solvents

All commercially available reagents were obtained from suppliers (ABCR GmbH, Apollo Scientific Ltd., Sigma Aldrich, Fluorochem Ltd) and used without further purification unless otherwise noted. Anhydrous solvents, stored over molecular sieves, were purchased from AcrosOrganics B.V.B.A. and used as received. The organocatalyst were purchased from commercial sources (ABCR, Acros Organics, Alfa–Aesar, Apollo Scientific, Sigma–Aldrich, Strem, TCI) or prepared according to literature.[4-6]

Nuclear Magnetic Resonance (NMR) Spectroscopy

1H NMR, 13C NMR, and 19F NMR spectra were recorded at ambient temperature on Bruker Neo 500 MHz and 400 MHz spectrometers as well as Bruker Ascend 400 MHz and Oxford 400 MHz spectrometers. Chemical shifts are reported in ppm with the solvent resonance as the reference unless noted otherwise ($CDCl_3$, ^{1}H: δ = 7.26 ppm, ^{13}C: δ = 77.2 ppm; Acetone-d_6, ^{1}H: δ = 2.05 ppm, ^{13}C: δ = 206.3, 29.9 ppm; MeCN-d_3, ^{1}H: δ = 1.94 ppm, ^{13}C: δ = 1.3, 118.3 ppm; DMSO-d_6, ^{1}H: δ = 2.50 ppm, ^{13}C: δ = 39.52 ppm). Peaks are reported as (s = singlet, d = doublet, dd = doublet of doublets, ddd = doublet od doublets of doublets, t = triplet, dt = doublet of triplets, tt = triplet of triplets, q = quartet, sept. = septet, m = multiplet or unresolved, br = broad signal, coupling constant(s) in Hz, integration).

Infrared (IR) Spectroscopy

IR were recorded neat on a Perkin-Elmer Spectrum Two FT-IR spectrometer. The main peaks are reported as absorption maxima (cm^{-1}).

High Resolution Mass Spectrometry (HRMS)

HRMS data were obtained at the mass spectrometry service operated by the Laboratory of Organic Chemistry at the ETHZ on a Bruker Daltonics maXis ESI-QTOF instrument or a Thermo Q-Exactive GC Orbitrap instrument and are reported as (m/z).

Reverse phase high performance liquid chromatography (HPLC)

Reverse phase high performance liquid chromatography (HPLC) was performed on a Dionex ultimate 3000 HPLC system (Thermo-Fischer) with a diode array detector or a Waters normal phase HPLC e2695 separations module with a 2998 PDA deterctor under the conditions given for each measurement.

Optical Rotation

Specific rotations (α) were measured on a Jasco P-2000 digital polarimeter at the sodium D line with a 10 cm cell length. Data are reported as follows: $[\alpha]^{T}$ in parentheses concentration (c = 1.00 corresponds to 10.0 mg·mL-1), and solvent. The temperature (T) at which the measurement was made is given as the superscript number (°C).

Chromatography

Analytical thin layer chromatography (TLC) was performed on Merck TLC Silica gel 60 F_{254} glass plates and visualized with 254 nm light and potassium permanganate or ceric ammonium molybdate staining solutions followed by heating.

$KMnO_4$ solution: $KMnO_4$ (3.0 g), 5 drops of conc. H_2SO_4 in water (300 mL).

Ceric ammonium molybdate solution: $Ce(SO_4)_2$ (5 g), $(NH_4)_6Mo_7O_{24}$*$4H_2O$ (25 g), conc.

H_2SO_4 (50 mL) in H_2O (450 mL).

4. Optimization Mukaiyama–Mannich Addition

Table S7: Optimization of Mukaiyama–Mannich Addition**.**

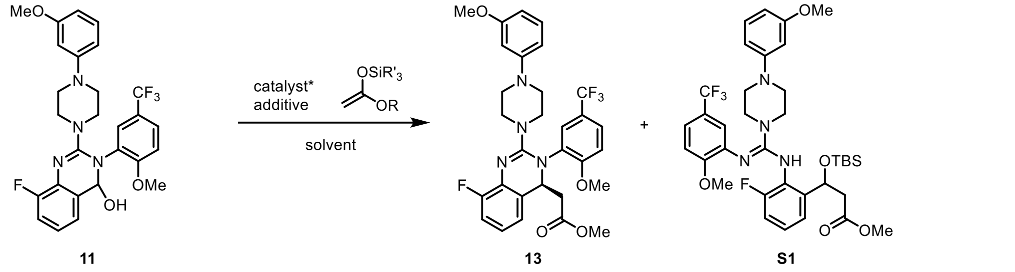

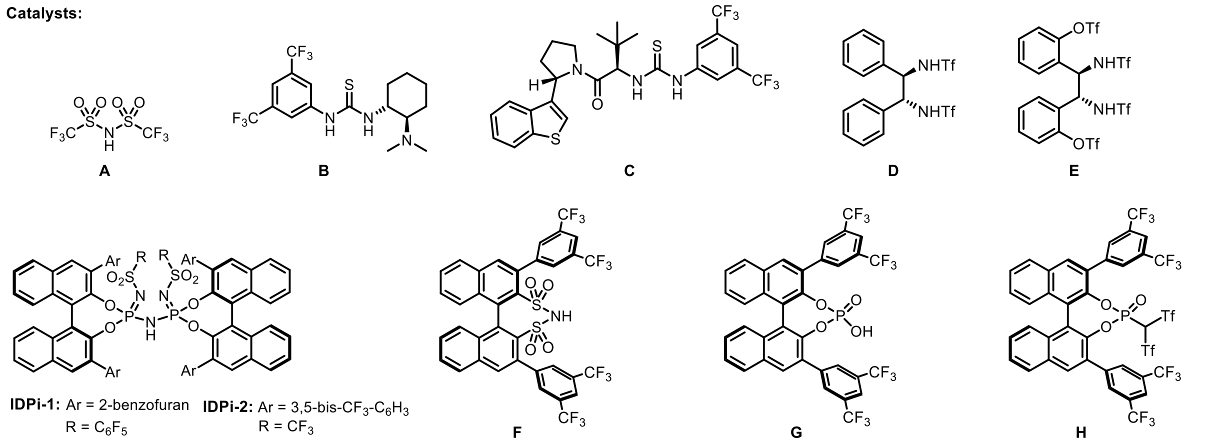

Entry	Catalyst	Additive	Temp [°C]	R	R'	Solvent	Yield [%] (11:S1)	er
1	A		25	Me	TBS	MeCN	70 (3:2)	-
2	B	TMSCl	–78 to 25 °C	Me	TBS	CH_2Cl_2	-	-
3	C	TMSCl	–78 to 25 °C	Me	TBS	CH_2Cl_2	-	-
4	D		25	Me	TBS	PhMe	N.D. (3:1)	50:50
5	E		25	Me	TBS	PhMe	N.D. (5:1)	50:50
6	**IDPi-1**		25	Me	TBS	PhMe	87 (3:1)	70:30
7	IDPi-2		25	Me	TBS	PhMe	55 (1:1)	60:40
8	**IDPi-1**		25	Me	TBS	PhMe:*n*-pentane	63 (3:1)	79:22
9	**IDPi-1**		25	i-Pr	TBS	PhMe:*n*-pentane	-	-
10	**IDPi-1**		25	Me	TES	PhMe:*n*-pentane	N.D.	77:23
11	**IDPi-1**		25	Me	TMS	PhMe:*n*-pentane	N.D.	62:38
12	F		25	Me	TBS	PhMe	N.D. (0:100)	-
13	G		25	Me	TBS	PhMe	-	-
14	H	*sec*-BuOH	25	Me	TBS	PhMe	32 (>20:1)	55:45
**15**	**IDPi-1**	***sec*-BuOH**	**25**	**Me**	**TBS**	**PhMe:pentane (1:1)**	**75 (>20:1)**	**90:10**
**16[a]**	**IDPi-1**	***sec*-BuOH**	**25**	**Me**	**TBS**	**PhMe:pentane (1:1)**	**75 (>20:1)**	**97:3**

*Ratios of **13** and **S1** were determined based on NMR ^1H analysis and **S1** was identified by LC-MS. Enantiomeric excess was determined by chiral reverse phase HPLC analysis. [a]Starting material was 3x evaporated with PhMe under inert atmosphere and in a flame-dried flask increased the er up to 97:3.

5. Mechanistic Studies

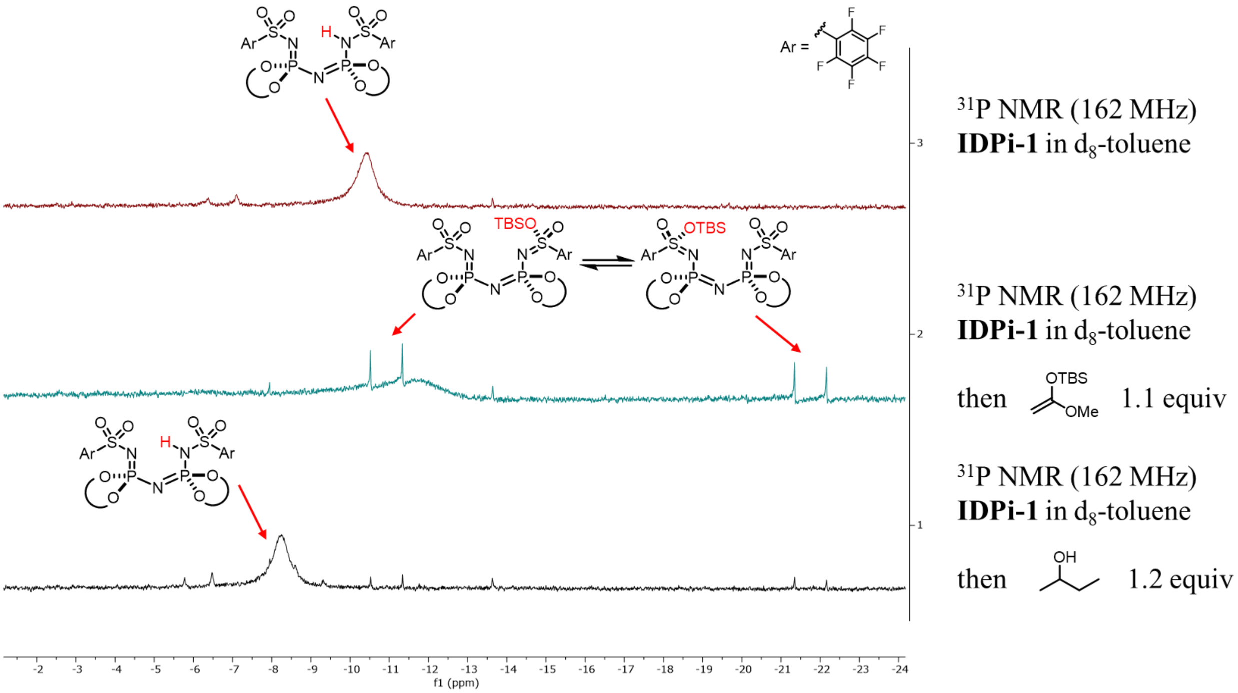

Figure S5: ^{31}P NMR studies with **IDPi-1** and sequential addition of SKA **12** and *sec*-BuOH.

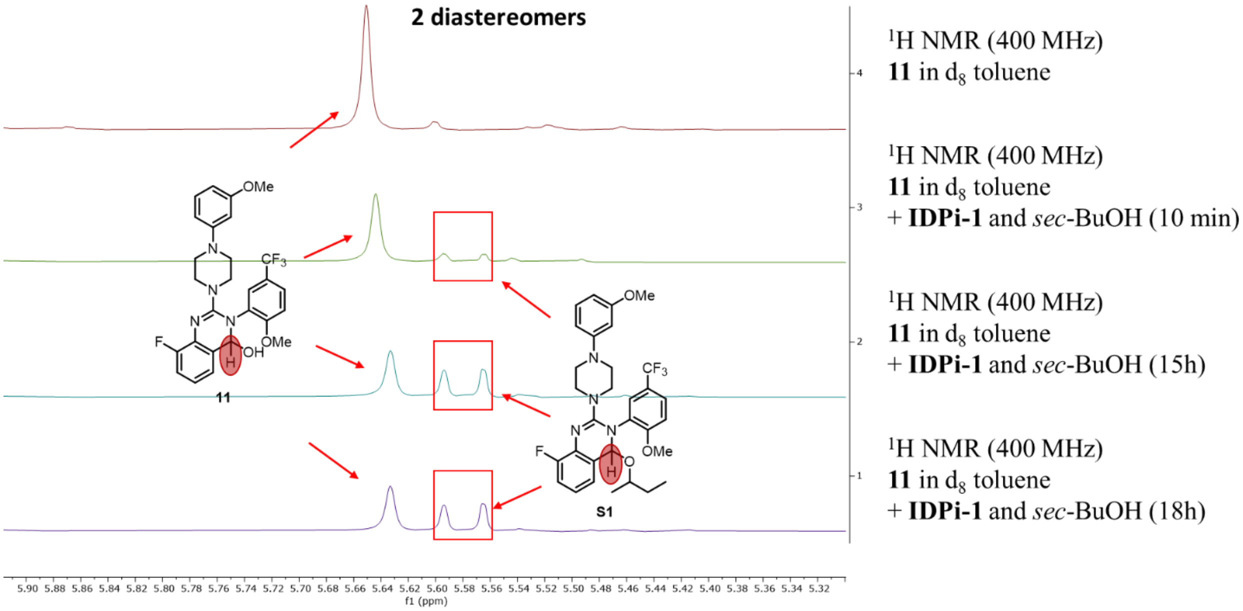

Figure S6: ^{1}H NMR studies to identify formation of S1 as competent intermediate in the Mukaiyama–Mannich reaction.

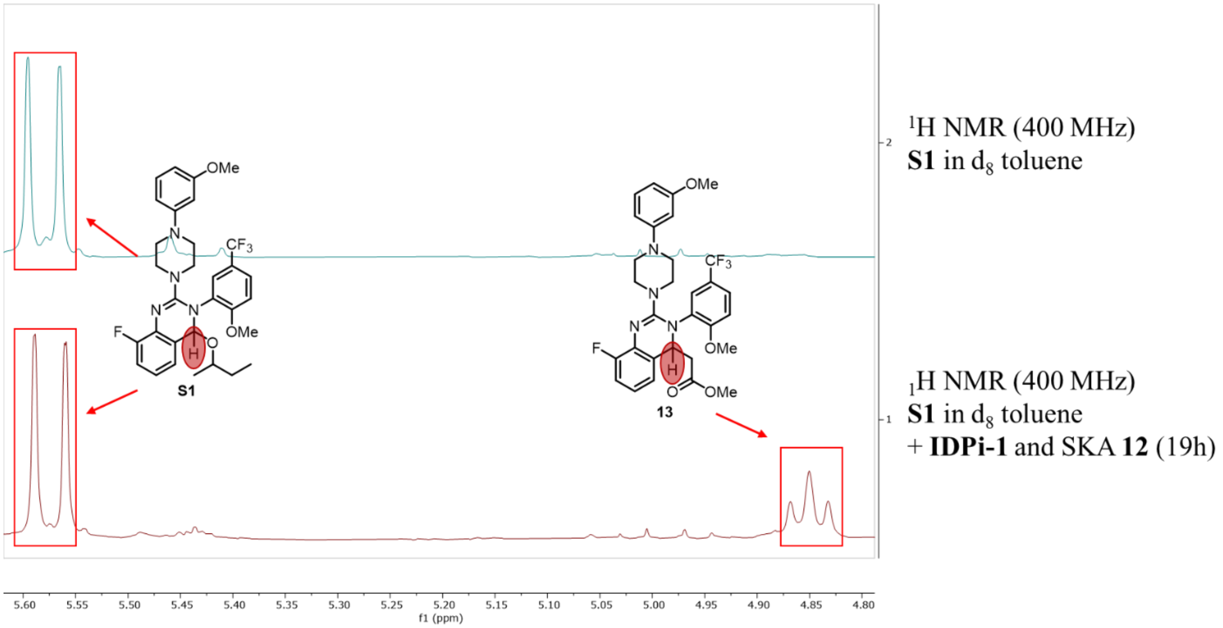

Figure S7: ^{1}H NMR studies to identify the conversion from **S1** to the desired product **13**.

2. Synthesis of IDPi

Scheme S1: Synthesis route of IPDi catalyst.

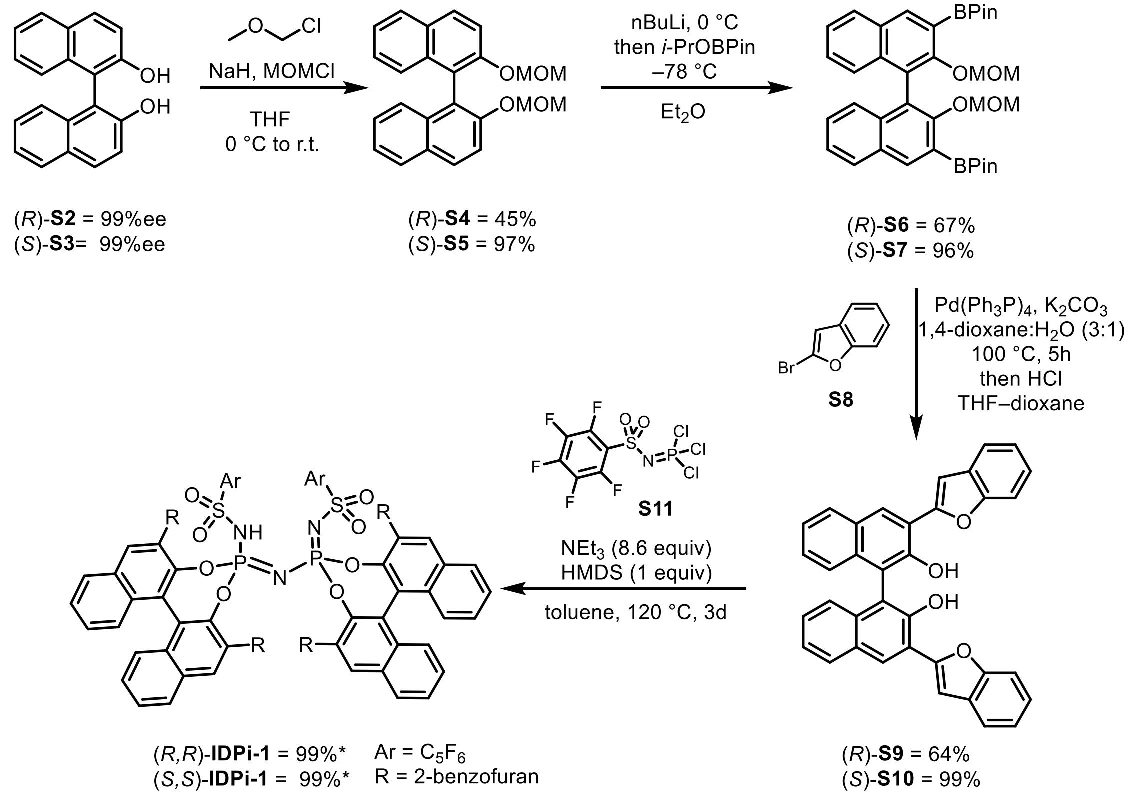

*Yield refers to the unpurified product, since over the course of the optimization it was identified that the unpurified IDPi was competent as catalyst in the Mukaiyama–Mannich addition. Isolated yields have been lower with 75%.

(*R*)- and (*S*) - 2,2'-bis(methoxymethoxy)-1,1'-binaphthalene (S4,S5)

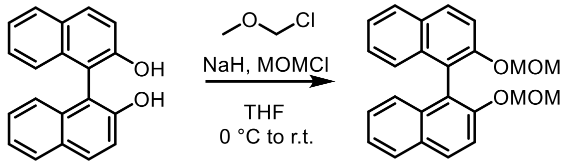

S2,S3 **S4,S5**

In a flame dried flask under nitrogen NaH 60% in oil (3.10 g, 76.8 mmol, 2.20 equiv) was added along with dry THF (100 mL) and stirred at 0 °C. To this stirring slurry a solution of (+)-BINOL (10.0 g, 34.9 mmol, 1.00 equiv) in THF (50.0 mL) was added dropwise via a dropping funnel. After complete addition the mixture was stirred at 0 °C for 1h before warming to room temperature and stir for another 30 min. The reaction mixture was cooled again to 0 °C and MOMCl (5.84 mL, 76.8 mmol, 2.20 equiv) dropwise via syringe. After the addition the reaction mixture was slowly warmed to room temperature and stirred overnight (ca. 12 h) or until full conversion was observed on TLC. Subsequently the reaction was quenched with sat. aq. NH_4Cl (75 mL) solution and the solvent was removed in vacuo. The obtained residue was diluted with CH_2Cl_2 (50 mL) and the aqueous phase was extracted with CH_2Cl_2 (3 x 20 mL). The organic layers were combined and washed with brine and dried over Na_2SO_4 and filtrated. The solvent was removed in vacuo. The material could be further purified either by flash column chromatography (Hex:EtOAc 10:1) or by recrystallization with Hex:CH_2Cl_2 to give the product (5.9 g, 15.8 mmol, 45%) as white crystalline solid.

(*R*)- 2,2'-bis(methoxymethoxy)-1,1'-binaphthalene (S4)

^{1}H NMR (400 MHz, $CDCl_3$) δ 7.95 (dt, J = 9.0, 0.6 Hz, 1H), 7.89 – 7.86 (m, 1H), 7.58 (d, J = 9.0 Hz, 1H), 7.34 (ddd, J = 8.1, 6.7, 1.3 Hz, 1H), 7.22 (ddd, J = 8.0, 6.7, 1.3 Hz, 1H), 7.15 (ddt, J = 8.5, 1.4, 0.8 Hz, 1H), 5.08 (d, J = 6.8 Hz, 1H), 4.98 (d, J = 6.7 Hz, 1H), 3.15 (s, 3H).

^{13}C NMR (101 MHz, $CDCl_3$) δ 152.80, 134.17, 130.03, 129.53, 128.00, 126.43, 125.70, 124.21, 121.46, 117.46, 95.38, 55.97.

IR (Diamond-ATR, neat): $\tilde{\nu}$ / cm^{-1} = 2901, 1622, 1592, 1506, 1475, 1238, 1147, 1068, 1032, 1012, 920, 809, 749.

HRMS (ESI) calcd for $C_{24}H_{22}NaO_4$ [M-Na]$^+$: 397.1410, found 397.1409.

TLC R_f (Hex:EtOAc 9:1) = 0.26 (UV-active, Ceric ammonium molybdate)

Optical rotation α_D = +85.556 ±0.449 (25 °C, c = 1.0)

(*S*)-2,2'-bis(methoxymethoxy)-1,1'-binaphthalene (S5)

Following the procedure above for the (R)-enantiomer with (–)-BINOL (5.00 g, 17.5 mmol, 1.00 equiv), NaH 60% in oil (1.50 g, 38.4 mmol, 2.20 equiv), MOMCl (2.90 ml, 38.4 mmol, 2.20 equiv) and in total 75.0 mL of THF. Product (6.30 g, 17.0 mmol, 97%) was obtained as white crystalline.

^{1}H NMR (500 MHz, $CDCl_3$) δ 7.59 (d, J = 9.1 Hz, 2H), 7.35 (ddd, J = 8.1, 6.7, 1.3 Hz, 2H), 7.23 (ddd, J = 8.6, 6.6, 1.3 Hz, 2H), 7.17 (ddt, J = 8.5, 1.4, 0.8 Hz, 2H), 5.09 (d, J = 6.8 Hz, 2H), 4.98 (d, J = 6.8 Hz, 2H), 3.15 (s, 6H).

^{13}C NMR (126 MHz, $CDCl_3$) δ 152.79, 134.16, 130.02, 129.52, 128.00, 126.42, 124.20, 121.45, 117.44, 95.36, 55.96.

IR (Diamond-ATR, neat): $\tilde{\nu}$ / cm^{-1} = 2902, 1622, 1592, 1506, 1475, 1238, 1197, 1148, 1069, 1014, 1033, 921, 810, 750.

HRMS (ESI) calcd for $C_{24}H_{22}NaO_4$ $[M\text{-}Na]^+$: 397.1410, found 397.1409.

TLC R_f (Hex:EtOAc 9:1) = 0.26 (UV-active, Ceric ammonium molybdate)

Optical rotation α_D = -85.187 ±0.183 (25 °C, c = 1.0)

(*R*)- and (*S*)-2,2'-(2,2'-bis(methoxymethoxy)-[1,1'-binaphthalene]-3,3'-diyl)bis(4,4,5,5-tetramethyl-1,3,2-dioxaborolane) (S6,S7)

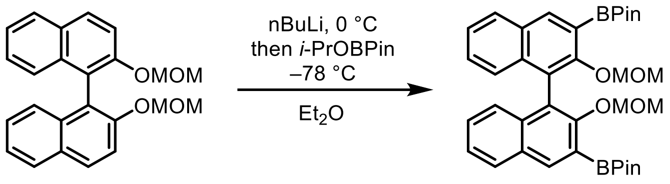

S4,S5 **S6,S7**

In a flame-dried flask under nitrogen the (R)-MOM-BINOL (3.70 g, 10.0 mmol, 1.00 equiv) was dissolved in 100 mL Et_2O and cooled to 0 °C with an ice-bath. *n*-BuLi 1.6M in hexane (18.7 mL, 30.0 mmol, 3.00 equiv) was added dropwise. The resultant reaction mixture was warmed slowly to room temperature and stirred for 3 h. The solution turned into a brownish emulsion. Afterwards the reaction solution was cooled with a dry-ice acetone bath to –78 °C and *i*-PrOBpin (8.20 mL, 40.0 mmol, 4.00 equiv) were added dropwise. The reaction mixture was then slowly warmed to room temperature and stirred for 12 h. The reaction solution became homogenous and yellowish. The reaction could be monitored via TLC and upon completion sat. aq. NH_4Cl solution (75 mL) and the aqueous phase was extracted with EtOAc (3 x 45 mL). The combined organic phases were washed with brine and dried over Na_2SO_4 and filtrated. Removal of the solvent under vacuum gave the unpurified material. Purification could be performed via flash column chromatography (Hex:EtOAc 9:1 to 8:2) or via recrystallization with Hex:EtOAc to afford the product (4.20 g, 6.70 mmol, 67%) as a yellow solid.

(*R*)-2,2'-(2,2'-bis(methoxymethoxy)-[1,1'-binaphthalene]-3,3'-diyl)bis(4,4,5,5-tetramethyl-1,3,2-dioxaborolane) (S6)

^{1}H NMR (400 MHz, $CDCl_3$) δ 8.45 (d, J = 0.7 Hz, 2H), 7.89 (dt, J = 8.1, 0.9 Hz, 2H), 7.36 (ddd, J = 8.1, 6.7, 1.3 Hz, 2H), 7.30 – 7.21 (m, 2H), 7.18 (dq, J = 8.6, 0.9 Hz, 2H), 4.90 – 4.85 (m, 4H), 2.28 (s, 6H), 1.38 (d, J = 2.3 Hz, 24H).

^{13}C NMR (101 MHz, $CDCl_3$) δ 157.19, 139.29, 136.26, 130.30, 128.36, 127.30, 126.89, 125.97, 124.74, 100.15, 83.96, 55.67, 25.02 (d, J = 3.7 Hz).

IR (Diamond-ATR, neat): $\tilde{\nu}$ / cm^{-1} = 2978, 2934, 1620, 1587, 1448, 1372, 1350, 1315, 1232, 1144, 1035, 980, 915, 867, 752, 669.

HRMS (ESI) calcd for $C_{36}H_{44}B_2NaO_8$ [M-Na]$^+$: 649.3114, found 649.3121.

TLC R_f (Hex:EtOAc 8:2) = 0.38 (UV-active, Ceric ammonium molybdate)

Optical rotation α_D = +34.074 ±0.499 (25 °C, c = 1.0)

(*S*)-2,2'-(2,2'-bis(methoxymethoxy)-[1,1'-binaphthalene]-3,3'-diyl)bis(4,4,5,5-tetramethyl-1,3,2-dioxaborolane) (S7)

Following the procedure above for the (R)-enantiomer with (–)-MOM-BINOL (5.00 g, 13.4 mmol, 1.00 equiv), *n*-BuLi 1.6M in hexane (25.1 mL, 40.1 mmol, 3.00 equiv), *i*-PrOBpin (10.9 ml, 53.5 mmol, 4.00 equiv) and 134 mL Et_2O. Product (8.10 g, 12.9 mmol, 96%) was obtained as yellow solid.

^{1}H NMR (400 MHz, $CDCl_3$) δ 8.45 (s, 2H), 7.89 (dt, J = 8.2, 0.9 Hz, 2H), 7.36 (ddd, J = 8.1, 6.7, 1.3 Hz, 2H), 7.30 – 7.21 (m, 2H), 7.18 (dd, J = 8.5, 1.2 Hz, 2H), 4.90 – 4.85 (m, 4H), 2.27 (s, 5H), 1.38 (d, J = 2.4 Hz, 24H).

^{13}C NMR (101 MHz, $CDCl_3$) δ 157.19, 139.28, 136.26, 130.29, 128.35, 127.29, 126.89, 125.97, 124.74, 100.14, 83.95, 55.66, 25.02 (d, J = 3.9 Hz).

IR (Diamond-ATR, neat): $\tilde{\nu}$ / cm^{-1} = 2978, 2934, 1621, 1587, 1448, 1350, 1315, 1232, 1208, 1144, 1035, 980, 914, 867, 752, 668.

HRMS (ESI) calcd for $C_{36}H_{44}B_2NaO_8$ [M-Na]$^+$: 649.3114, found 649.3121.

TLC R_f (Hex:EtOAc 8:2) = 0.38 (UV-active, Ceric ammonium molybdate)

Optical rotation α_D = -31.269 ±0.093 (25 °C, c = 1.0)

(*R*)- and (*S*)-3,3'-di(benzofuran-2-yl)-[1,1'-binaphthalene]-2,2'-diol (S9,S10)

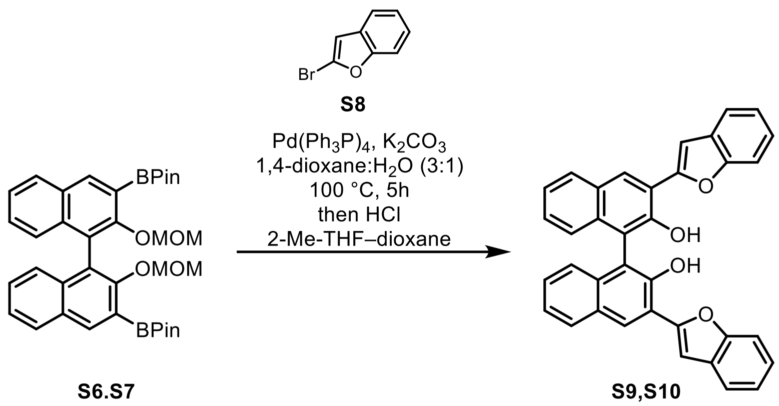

S6.S7 **S9,S10**

In a two-necked flask the (R)-Bpin-MOM-BINOL **S6** (0.590 g, 942 μmol, 1.00 equiv) was added along with 1,4-dioxane 9.0 mL, K_2CO_3 (729 mg, 5.30 mmol, 5.60 equiv) and water 3.00 mL. Then 2-bromo-benzofuran **S8** (501 mg, 2.50 mmol, 2.70 equiv) was added and the reaction solution was degassed by bubbling argon through the solution for 20 minutes. Then $Pd(PPh_3)_4$ (54.4 mg, 47.1 μmol, 0.05 equiv) were added and the reaction was heated to 100 °C under argon atmosphere. The reaction could be monitored via TLC and normally showed completion after 5-12 h. After cooling of the reaction mixture to room temperature sat. aq. NH_4Cl solution was added and the aqueous phase was extracted with EtOAc. The combined organic phases were washed with brine, dried over Na_2SO_4 then filtrated and concentrated under vacuo. The unpurified mixture was redissolved in THF 5.00 ml and HCl in dioxane 4M (4.20 mL, 17.0 mmol, 18.0 equiv) was added. This solution was further stirred for 16h at room temperature. Then EtOAc (5 mL) was added to dilute the mixture and aq. 1M HCl (5 mL) was added. The organic phase was separated and the aqueous phase was extracted with EtOAc (3 x 10 mL). The combined organic phases were washed once with sat. aq. $NaHCO_3$ solution (15 mL) and once with brine (15 mL) before drying over Na_2SO_4 and filtration. Concentration under vacuum afforded the unpurified material. Further purification was conducted via flash column chromatography (Hex:EtOAc 9:1). The product (488.0 mg, 941 μmol, 99% yield) was obtained as slightly brown foam.

Note: The THF in the second step as solvent for the hydrolysis of the MOM-ether could be substituted with 2-Me-THF for the LCA analysis.

(*R*)- 3,3'-di(benzofuran-2-yl)-[1,1'-binaphthalene]-2,2'-diol (S9)

^{1}H NMR (400 MHz, $CDCl_3$) δ 8.79 (s, 2H), 8.05 (ddd, J = 8.6, 1.2, 0.6 Hz, 2H), 7.65 – 7.59 (m, 4H), 7.52 (d, J = 0.9 Hz, 2H), 7.44 (ddd, J = 8.1, 6.8, 1.2 Hz, 2H), 7.34 (dddd, J = 8.2, 6.7, 5.5, 1.3 Hz, 4H), 7.28 – 7.23 (m, 2H), 7.18 (dq, J = 8.4, 0.8 Hz, 2H), 5.88 (d, J = 0.6 Hz, 2H).

^{13}C NMR (101 MHz, $CDCl_3$) δ 154.44, 151.81, 149.99, 133.02, 129.70, 129.45, 129.20, 128.55, 128.27, 125.00, 124.25, 123.16, 121.62, 119.55, 111.97, 111.10, 107.88.

IR (Diamond-ATR, neat): $\tilde{\nu}$ / cm^{-1} = 3502, 3062, 1618, 1604, 1504, 1456, 1386, 1360, 1296, 1257, 1210, 1196, 1168, 1147, 1123, 1107, 1010, 924, 900, 819, 778, 750.

HRMS (ESI) calcd for $C_{36}H_{22}NaO_4$ [M-Na]$^+$: 541.1410, found 541.1410.

TLC R_f (Hex:EtOAc 9:1) = 0.53 (UV-active, Ceric ammonium molybdate)

Optical rotation α_D = -82.735 ±0.220 (25 °C, c = 1.0)

(*S*)-3,3'-di(benzofuran-2-yl)-[1,1'-binaphthalene]-2,2'-diol (S10)

Following the procedure above for the (R)-enantiomer with (S)-Bpin-MOM-BINOL **S7** (590 mg, 942 μmol, 1.00 equiv), 1,4-dioxane 9.00 mL, K_2CO_3 (729 mg, 5.30 mmol, 5.60 equiv), water 3.00 mL, 2-bromo-benzofuran **S8** (501 mg, 2.50 mmol, 2.70 equiv) and $Pd(PPh_3)_4$ (54.4 mg, 47.1 μmol, 0.05 equiv). Second step with 5.00 mL of THF and HCl in dioxane 4M (4.20 mL, 17.0 mmol, 18.0 euqiv). The product (482 mg, 941 μmol, 99% yield) was obtained as off-white solid.

^{1}H NMR (400 MHz, $CDCl_3$) δ 8.79 (s, 2H), 8.08 – 8.01 (m, 2H), 7.62 (dddd, J = 8.4, 7.6, 1.5, 0.8 Hz, 4H), 7.48 (d, J = 1.0 Hz, 2H), 7.44 (ddd, J = 8.1, 6.8, 1.2 Hz, 2H), 7.34 (dddd, J = 15.3, 8.2, 7.0, 1.3 Hz, 4H), 7.29 – 7.23 (m, 2H), 7.19 (ddd, J = 8.4, 5.9, 2.0 Hz, 2H), 5.88 (s, 2H).

^{13}C NMR (101 MHz, $CDCl_3$) δ 154.39, 151.76, 149.96, 132.99, 129.68, 129.41, 129.18, 128.49, 128.21, 124.96, 124.20, 123.11, 121.61, 119.51, 111.92, 111.06, 107.85.

IR (Diamond-ATR, neat): $\tilde{\nu}$ / cm^{-1} = 3500, 3062, 1618, 1604, 1503, 1456, 1386, 1360, 1296, 1257, 1210, 1196, 1168, 1147, 1124, 1107, 1010, 824, 889, 819, 778, 751.

HRMS (ESI) calcd for $C_{36}H_{22}NaO_4$ [M-Na]$^+$: 541.1410, found 541.1409.

TLC R_f (Hex:EtOAc 9:1) = 0.53 (UV-active, Ceric ammonium molybdate)

Optical rotation α_D = +72.855 ±0.473 (25 °C, c = 1.0)

((2,3,4,5,6-pentamethylphenyl)sulfonyl)phosphorimidoyl trichloride

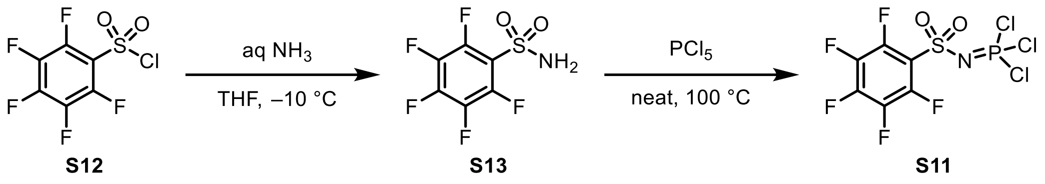

Step 1:

Pentafluorobenzenesulfonyl chloride **S12** (10.0 g, 5.60 mL, 37.5 mmol, 1.00 equiv) was dissolved in THF (600 mL) and cooled to –10 °C with a salt-ice bath and aqueous ammonia 25% (12.0 mL, 159 mmol, 4.20 equiv) was added dropwise to the stirring reaction solution. The reaction was monitored via TLC and upon completion the THF was evaporated under vacuo. The resultant precipitate was washed with H_2O and could be further recrystallized from H_2O to increase purity if needed. The product **S13** (8.10 g, 32.8 mmol, 87%) was obtained as off-white solid.

^{1}H NMR (500 MHz, DMSO-d_6) δ 8.47 (s, 2H).

^{13}C NMR (126 MHz, DMSO-d_6) δ 144.96 – 142.51 (m), 144.45 – 141.95 (m), 139.12 – 138.70 (m), 137.08 – 136.70 (m), 119.52 (t, J = 16.0 Hz).

^{19}F NMR (471 MHz, DMSO-d_6) δ -138.83 – -139.18 (m), -148.85 (tt, J = 22.3, 5.3 Hz), -160.17 – -160.88 (m).

IR (Diamond-ATR, neat): $\tilde{\nu}$ / cm^{-1} = 3342, 3263, 1644, 1512, 1493, 1354, 1302, 1168, 1096, 989, 932, 728, 644, 613.

HRMS (EI) calcd for $C_6H_2F_5NO_2S$ [M]$^+$: 246.9721, found 246.9720.

The analytical data is in full agreement with the reported literature.[7]

Step 2:

The pentafluorobenzene sulfonamide **S13** (200 mg, 809 mmol, 1.00 equiv) from step 1 and PCl_5 (185 mg, 890 mmol, 1.10 equiv) were placed in a flame-dried Schlenk tube under nitrogen atmosphere with a stirring bar. The reaction mixture was heated to 100 °C with an outlet attached to a HCl trap (filled with sat. aq. $NaHCO_3$). The continuous nitrogen flow could be tested for acidity (HCl release) with a wet piece of pH-paper. Upon no significant color change on the pH-paper was observed the HCl trap outlet was removed and the Schlenk tube was sealed to be kept at this temperature under high vacuum (1 h). Then the Schlenk tube was flushed with nitrogen and the set up was changed once again to remove excess of PCl_5 by sublimation under

reduced pressure. The residue obtained was analyzed by NMR and showed the characteristic ^{19}F and ^{31}P signals. The material **S11** could be stored in the freezer (became an off-white solid at lower temperatures) and directly be used for the subsequent step.

Note: Information about the set up for this procedure are reported in form of photographs just below. The sublimation was performed multiple times until no visible solid could be observed anymore at the cooling finger.

^{19}F NMR (377 MHz, CD_2Cl_2) δ -159.84, -146.82, -136.76.

^{31}P NMR (162 MHz, CD_2Cl_2) δ 9.62.

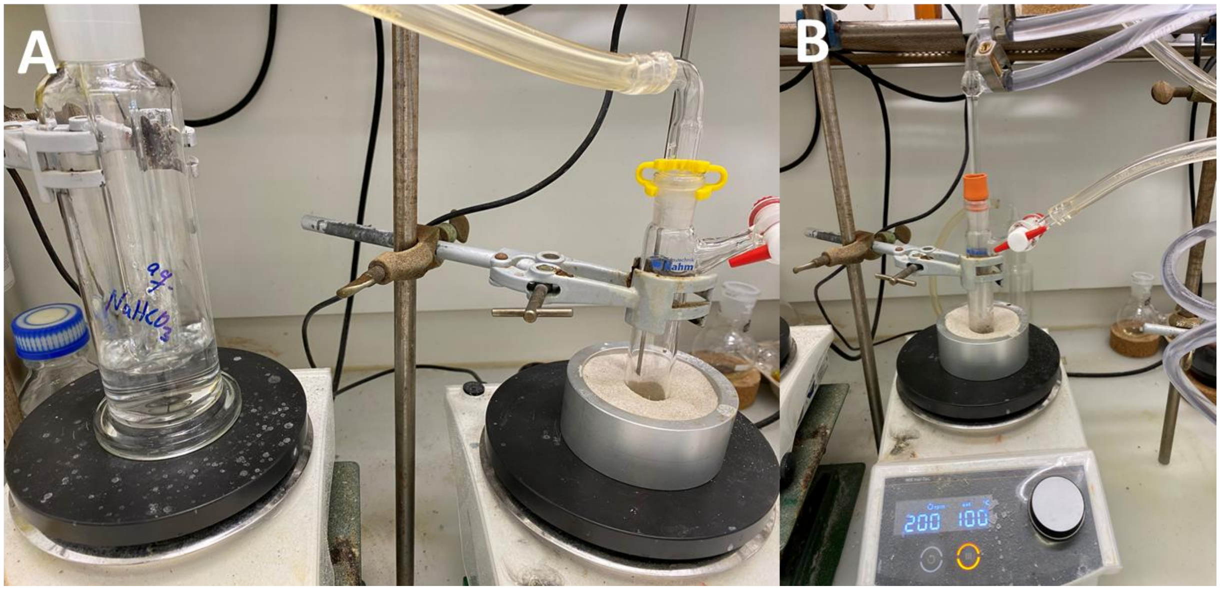

Figure S8: Photo of the experimental set up for the preparation of **S11**. A) Step 1 from the experimental procedure. B) Step 2 from the experimental procedure.

Synthesis of (*R*,*R*)-and (*S*,*S*)-O-cat:

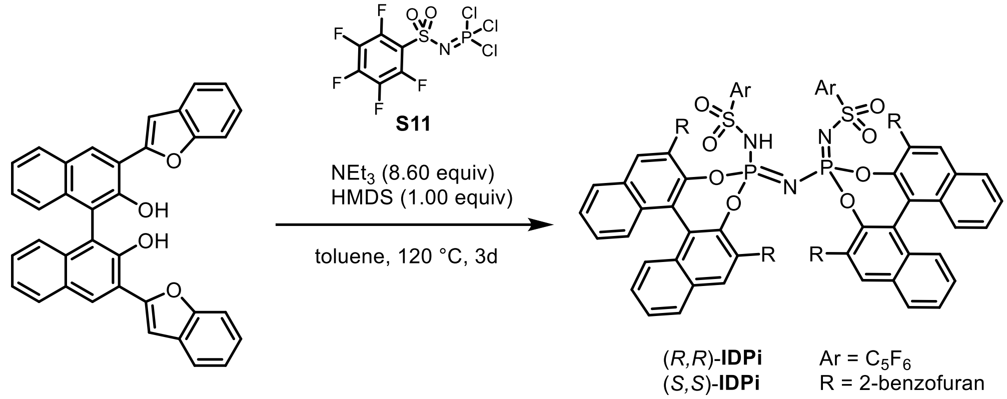

S9, S10

BINOL **S9** or **S10** (198 mg, 382 μmol, 2.00 equiv) and **S11** (146 mg, 382 μmol, 2.00 equiv) were placed into a flame dried Schlenk flask under inert atmosphere (or in the glovebox). The solids were gently heated with a heatgun under vacuum until melting is observed (ca. 50 °C). Then toluene (4.00 mL) is added, followed by Et_3N (229 μl, 1.64 mmol, 8.60 equiv) and the mixture was stirred for 1 h at room temperature. Monitoring by TLC revealed full consumption of the (*R*)-BINOL **S9** starting material (new spot R_f (Hex:EtOAc 9:1) = baseline). HMDS (40.0 μl, 191 μmol, 1.00 equiv) was added and the mixture was further stirred at room temperature for 10 min and was subsequently heated to 120 °C for 3 days. TLC indicated again full consumption of the before observed intermediate (new spot R_f (Hex:EtOAc 2:1) = 0.35). After cooling the reaction mixture to room temperature CH_2Cl_2 (5 mL) was added for dilution and the organic phase was washed with aq. 2M HCl (5 mL). The aqueous layer was extracted with CH_2Cl_2 (3 x 5 mL), the organic phases were combined, dried over Na_2SO_4 and after filtration the solvent was removed *in vacuo*.

Further purification could be achieved by flash column chromatography with a gradient of 2-5% EtOAc in CH_2Cl_2.

The obtained product was acidified by filtration over a short plug of DOWEX 50WX8 (H-form, eluted with 5%EtOAc in CH_2Cl_2). The DOWEX plug was rinsed through with a 0.0125M aq H_2SO_4 solution, followed by ethanol, followed by 5% EtOAc in CH_2Cl_2 mixture before applying the material. The solvent was removed *in vacuo* and the obtained residue was dissolved in as little as possible CH_2Cl_2 and subsequent addition of excess *n*-pentane resulted in precipitation of an off-white solid. Evaporation of the solvents gave a bench stable off-white powder.

Note: Over the course of the study it was found that with these slightly adapted conditions in comparison to the literature[3] *the product could be obtained in nearly quantitative yield with only small impurities detected by NMR analysis. In that case unpurified product was directly acidified*

following the above described conditions. These "unpurified catalysts" were tested in the Mukaiyama–Mannich addition and very similar results were be obtained.

(*R*,*R*)-IDPi:

^{1}H NMR (500 MHz, CD_2Cl_2) δ 8.56 (s, 2H), 8.12 (d, J = 8.2 Hz, 2H), 8.06 (d, J = 8.4 Hz, 2H), 7.84 (s, 2H), 7.73 (d, J = 7.7 Hz, 2H), 7.67 – 7.49 (m, 8H), 7.44 – 7.36 (m, 2H), 7.32 (dd, J = 8.7, 6.7 Hz, 4H), 7.29 – 7.05 (m, 10H), 6.79 (s, 4H), 6.63 – 6.54 (m, 2H).

^{13}C NMR (126 MHz, CD_2Cl_2) δ 154.67, 154.45, 149.98, 149.70, 142.76 – 142.48 (m), 141.97 (t, J = 5.4 Hz), 132.16, 132.12, 131.93, 129.79, 129.41 (d, J = 6.9 Hz), 128.89, 128.11, 128.03, 127.84, 127.52, 127.21, 125.92, 125.01, 123.80, 123.28, 122.85, 122.56, 122.24, 121.98, 121.85, 111.21, 110.95, 109.09, 108.23.

^{19}F NMR (471 MHz, CD_2Cl_2) δ -136.77, -145.65, -159.59.

^{31}P NMR (202 MHz, CD_2Cl_2) δ -16.86.

IR (Diamond-ATR, neat): $\tilde{\nu}$ / cm^{-1} = 2924, 2852, 1519, 1494, 1456, 1421, 1378, 1324, 1297, 1261, 1251, 1208, 1186, 1152, 1106, 1097, 1010, 989, 979, 964, 948, 934, 896, 886, 875, 837, 752, 740, 706, 643, 606.

HRMS (ESI) calcd for $C_{84}H_{42}F_{10}N_3O_{12}P_2S_2$ [M-H]$^{+}$: 1600.1520, found 1600.1516.

TLC R_f (CH_2Cl_2:EtOAc 9:1) = 0.21 (UV-active, Ceric ammonium molybdate)

Optical rotation α_D = -426.593 ±0.689 (25 °C, c = 1.0)

(*S*,*S*)-IDPi:

^{1}H NMR (400 MHz, CD_2Cl_2) δ 8.55 (s, 2H), 8.13 (d, J = 8.2 Hz, 2H), 8.07 (d, J = 8.3 Hz, 2H), 7.85 (t, J = 7.5 Hz, 2H), 7.74 (d, J = 7.7 Hz, 2H), 7.64 – 7.52 (m, 8H), 7.43 – 7.36 (m, 2H), 7.34 – 7.25 (m, 4H), 7.13 (m, 10H), 6.78 (d, J = 4.9 Hz, 4H), 6.57 (ddd, J = 8.0, 5.3, 2.9 Hz, 2H), 4.41 (s, 1H).

^{13}C NMR (101 MHz, CD_2Cl_2) δ 154.49, 154.27, 149.82, 149.55, 142.47 (t, J = 5.0 Hz), 131.99, 131.95, 131.76, 129.64, 129.27, 129.21, 128.70, 127.94, 127.84, 127.68, 127.33, 127.04, 125.73, 124.83, 123.62, 123.11, 122.66, 122.40, 122.10, 121.83, 121.68, 111.03, 110.77, 108.91, 108.08.

^{19}F NMR (377 MHz, CD_2Cl_2) δ -136.77, -145.80, -159.77.

^{31}P NMR (162 MHz, CD_2Cl_2) δ -16.39.

IR (Diamond-ATR, neat): $\tilde{\nu}$ / cm^{-1} = 3063, 2962, 2924, 1644, 1605, 1519, 1493, 1455, 1422, 1296, 1260, 1184, 1151, 1097, 1077, 1009, 989, 934, 896, 818, 750, 741, 705, 605.

HRMS (ESI) calcd for $C_{84}H_{42}F_{10}N_3O_{12}P_2S_2$ [M-H]$^+$: 1600.1520, found 1600.1516.

TLC R_f (CH_2Cl_2:EtOAc 9:1) = 0.21 (UV-active, Ceric ammonium molybdate)

Optical rotation α_D = +482.657 ±0.876 (25 °C, c = 1.0)

6. Experimental Section of Letermovir Route

(2-amino-3-fluorophenyl)methanol (3)

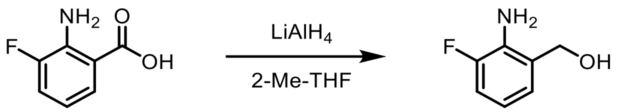

2 **3**

Procedure 1:

In a 500 mL flask was filled $LiAlH_4$ (6.51 g, 172 mmol, 2.00 equiv) and 73.0 mL of 2-Me-THF was added. This slurry was stirred at room temperature and a solution of 3-fluoro-anthranilic acid **2** (13.3 g, 85.7 mmol, 1.00 equiv) in 60.0 mL of 2-Me-THF was added dropwise over 20 minutes. After stirring for 4 h at ambient temperature the TLC indicated full conversion of starting material and the reaction was diluted with wet ether. Afterwards 6.51 mL of H_2O, 6.51 mL of 15% aq. NaOH, 19.5 mL H_2O and $MgSO_4$ were sequentially added. The slurry was stirred at room temperature for 30 minutes before filtration over Celite gave a solution of product in 2-Me-THF.

The product solution in 2-Me-THF was used without further purification in the next step.

For characterization purposes an aliquot of the mixture was evaporated and analyzed.

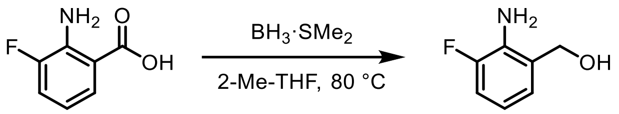

2 **3**

Procedure 2:

3-fluoro-anthranilic acid **2** (20.0 g, 129 mmol, 1.00 equiv) was charged in a round bottom flask and was dissolved in 2-Me-THF (120 ml, 1.30M). Then $BH_3{\cdot}SMe_2$ (15.5 mL, 155 mmol, 1.20 equiv) was added dropwise and gas evolution was observed along with a white precipitate formed. The reaction mixture was subsequently heated to 80 °C, upon the white precipitate started dissolving. The reaction was monitored by LC-MS until full conversion was observed. Then the reaction mixture was cooled to r.t. and MeOH (20.9 mL, 516 mmol, 4.00 equiv) was added. This mixture was stirred at r.t. for 2-3 h before 0.10M aq NaOH solution (75.0 mL) was added and further stirred for a while. Separation of the organic phase and evaporation of the volatiles affords the product as off-white solid.

^{1}H NMR (400 MHz, $CDCl_3$) δ 6.97 (ddd, J = 10.9, 8.2, 1.4 Hz, 1H), 6.86 (ddd, J = 7.5, 1.5, 0.8 Hz, 1H), 6.64 (ddd, J = 8.1, 7.5, 5.1 Hz, 1H), 4.70 (s, 2H).

^{13}C NMR (101 MHz, $CDCl_3$) δ 152.08 (d, J = 238.4 Hz), 134.44 (d, J = 13.1 Hz), 127.13 (d, J = 3.2 Hz), 124.28 (d, J = 3.0 Hz), 117.51 (d, J = 7.5 Hz), 115.17 (d, J = 19.1 Hz).

^{19}F NMR (377 MHz, $CDCl_3$) δ -136.29.

IR (Diamond-ATR, neat): $\tilde{\nu}$ / cm^{-1} = 3339, 1629, 1529, 1488, 1388, 1335, 1269, 1233, 1205, 1046, 1010, 960, 915, 781, 749.

HRMS (ESI) calcd for C_7H_9FNO [M-H]$^+$: 142.0663, found 142.0662.

TLC R_f (Hex:EtOAc 7:3) = 0.21 (UV-active, Ceric ammonium molybdate)

2-isothiocyanato-1-methoxy-4-(trifluoromethyl)benzene (4)

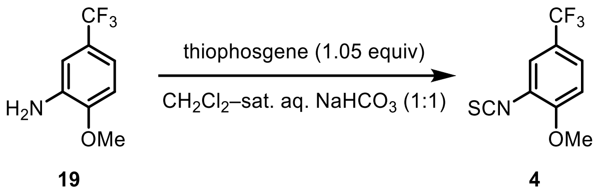

3-amino-4-methoxybenzotrifluoride (5.00 g, 26.2 mmol, 1.00 equiv) was dissolved in CH_2Cl_2 (100 mL) and sat. aq. $NaHCO_3$ (100 mL) was added. To this biphasic mixture thiophosgene (2.11 mL, 27.5 mmol, 1.05 equiv) was added dropwise and the reaction mixture was subsequently vigorously stirred until full conversion was observed on TLC (1-2 h). The reaction mixture phases were separated, and the organic phase was washed with water (30 mL) and the aqueous phase was extracted with CH_2Cl_2 (2 x 15 mL). The combined organic phases were washed with brine (75 mL) and dried over Na_2SO_4. Evaporation of the solvent led to the desired product as orange oil (6.10 g, 26.2 mmol, 100%).

^{1}H NMR (500 MHz, $CDCl_3$) δ 7.50 – 7.46 (m, 1H), 7.34 (dd, J = 2.3, 0.7 Hz, 1H), 6.97 (dd, J = 8.8, 0.9 Hz, 1H), 3.97 (s, 3H).

^{13}C NMR (126 MHz, $CDCl_3$) δ 158.47 (q, J = 0.9 Hz), 142.44, 125.25 (q, J = 3.8 Hz), 123.68 (q, J = 271.5 Hz), 123.34 (q, J = 33.6 Hz), 122.49 (q, J = 3.7 Hz), 121.74, 111.38, 56.46.

^{19}F NMR (471 MHz, $CDCl_3$) δ -61.89.

IR (Diamond-ATR, neat): $\tilde{\nu}$ / cm-1 = 2196, 2013, 1617, 1510, 1461, 1432, 1329, 1278, 1262, 1225, 1168, 1116, 2076, 1022, 970, 892, 815, 696, 646, 619, 611.

HRMS (EI) calcd for $C_9H_6ONF_3S$ $[M]^+$: 233.0117, found 233.0113.

TLC R_f (Hex:EtOAc 9:1) = 0.68 (UV-active, Ceric ammonium molybdate)

1-(2-fluoro-6-(hydroxymethyl)phenyl)-3-(2-methoxy-5-(trifluoromethyl)phenyl)thiourea (5)

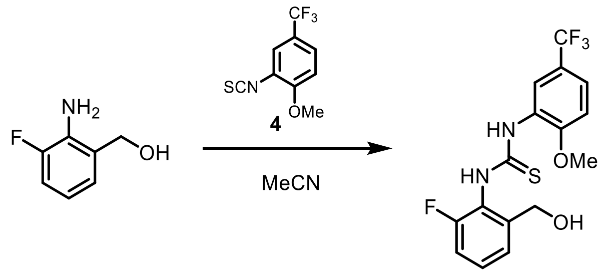

3 5

Benzylic alcohol **3** (200 mg, 1.42 mmol, 1.00 equiv) and isothiocyanate 4 (347 mg, 1.49 mmol, 1.05 equiv) were placed into a flask and MeOH (5.00 mL) was added. This mixture was stirred until full conversion was observed by TLC or LC-MS (normally 14-20 h). After full conversion the solvent was evaporated and the unpurified product was subjected to flash column chromatography with a gradient from 7:3 to 1:1 (Hex:EtOAc). The product (527 mg, 1.41 mmol, 99% yield) was isolated as colorless crystalline solid.

^{1}H NMR (500 MHz, Acetone-d_6) δ 8.98 (s, 1H), 8.91 (s, 1H), 8.78 (d, J = 2.3 Hz, 1H), 7.49 (ddd, J = 8.6, 2.3, 0.9 Hz, 1H), 7.44 – 7.35 (m, 2H), 7.22 (d, J = 8.6 Hz, 1H), 7.14 (ddd, J = 9.7, 7.5, 2.2 Hz, 1H), 4.75 (s, 2H), 4.49 – 4.34 (m, 1H), 3.94 (s, 3H).

^{13}C NMR (126 MHz, Acetone-d$_6$) δ 182.44, 159.30 (d, J = 247.4 Hz), 154.76, 143.83, 129.80 (d, J = 8.5 Hz), 129.32, 125.38 (q, J = 270.6 Hz), 124.85 – 124.49 (m), 124.07 (d, J = 3.3 Hz), 123.53 (q, J = 4.0 Hz), 122.29 (q, J = 32.6 Hz), 121.72 (q, J = 3.9 Hz), 115.27 (d, J = 20.7 Hz), 112.11, 61.05 (d, J = 3.0 Hz), 56.70.

^{19}F NMR (471 MHz, Acetone-d_6) δ -61.89, -122.22.

IR (Diamond-ATR, neat): $\tilde{\nu}$ / cm^{-1} = 3299, 2943, 1618, 1538, 1473, 1447, 1332, 1261, 1167, 1120, 1077, 1024, 817, 619.

HRMS (ESI) calcd. for $C_{16}H_{15}F_4N_2O_2S$ [M+H$^+$]: 375.0785, found 375.0784.

TLC R_f (Hex:EtOAc 1:1) = 0.21 (UV-active, Ceric ammonium molybdate)

8-fluoro-N-(2-methoxy-5-(trifluoromethyl)phenyl)-4H-benzo[d][1,3]thiazin-2-amine·HCl (8)

Procedure 1:

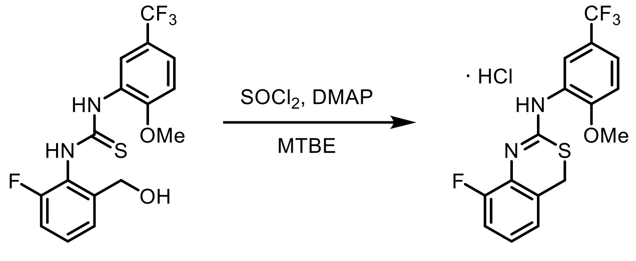

5 **8**

Benzylic alcohol **5** (2.10 g, 5.61 mmol, 1.00 equiv) was dissolved in MTBE (50.0 mL, 0.11M). DMAP (6.85 mg, 56.1 µmol, 0.01 equiv) was added at once and afterwards thionyl chloride (0.488 mL, 6.73 mmol, 1.20 equiv) was added dropwise. Upon addition a precipitate occurred. The reaction was further stirred for 5 h until full conversion was observed. The precipitate was filtered and the filter cake washed with MTBE (3 x 20 mL) to obtain a colorless solid as product (2.12 g, 5.40 mmol, 96%).

Procedure 2:

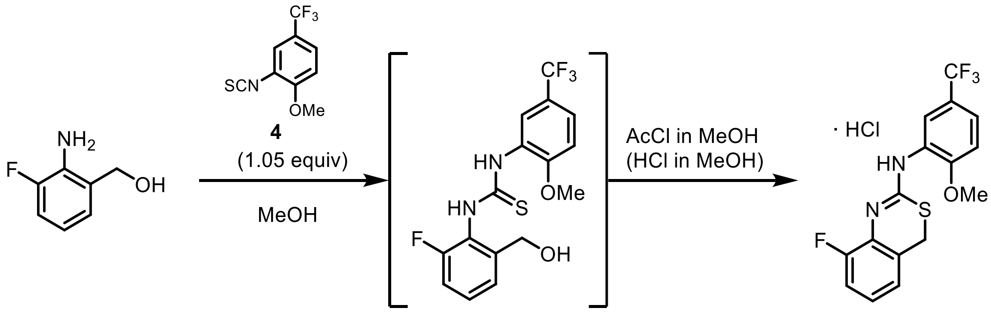

3 **5** **8**

Benzylic alcohol **3** (3.69 g, 26.2 mmol, 1.00 equiv.) was dissolved in methanol (25.0 mL) and the isothiocyanate **4** (6.10 g, 26.2 mmol, 1.00 equiv.) was added. This reaction was stirred at ambient temperature and monitored either by LC-MS and/or TLC (ca. 19 h). Until full conversion was observed the reaction was cooled to 0 °C. A solution of 10.0 mL methanol with AcCl (2.85 mL, 40.0 mmol, 1.53 equiv.) was prepared carefully at 0 °C. This solution was added to the reaction solution at 0 °C. The reaction was further stirred at ambient temperature until full conversion was observed via LC-MS and/or TLC (ca. 16 h). Over the cause of the reaction precipitate occurred that was after completion of the reaction filtered and dried under vacuum to obtain the product as off-white powder (10.1 g, 25.7 mmol, 98%).

^{1}H NMR (500 MHz, DMSO-d_6) δ 10.50 (br s, 2H) 8.53 (s, 1H), 7.60 – 7.54 (m, 1H), 7.31 (d, J = 8.6 Hz, 1H), 7.24 (ddd, J = 10.3, 8.1, 1.6 Hz, 1H), 7.20 – 7.10 (m, 2H), 4.21 (s, 2H), 3.93 (s, 3H).

^{13}C NMR (126 MHz, DMSO-d_6) δ 154.85, 153.48, 152.88, 127.55, 125.39, 123.48, 123.23, 122.82 (d, J = 3.2 Hz), 121.06 (q, J = 32.3 Hz), 114.99, 112.04, 56.36, 28.23 (d, J = 2.8 Hz).

^{19}F NMR (471 MHz, DMSO-d_6) δ -60.10, -128.08.

IR (Diamond-ATR, neat): $\tilde{\nu}$ / cm^{-1} = 1633, 1610, 1583, 1556, 1509, 1484, 1462, 1442, 1431, 1414, 1385, 1326, 1293, 1278, 1259, 1253, 1222, 1201, 1180, 1171, 1156, 1148, 1135, 1122, 1071, 1020, 966, 953, 916, 884, 866, 832, 803, 782, 743, 729.

HRMS (ESI) calcd. for $C_{16}H_{13}F_4N_2OS$ [M+H$^+$]: 357.0679, found 357.0677.

TLC R_f (Hex:EtOAc 6:4) = 0.58 (UV-active, Ceric ammonium molybdate)

8-fluoro-2-((2-methoxy-5-(trifluoromethyl)phenyl)amino)-4H-benzo[d][1,3]thiazin-4-ol (7)

Procedure 1:

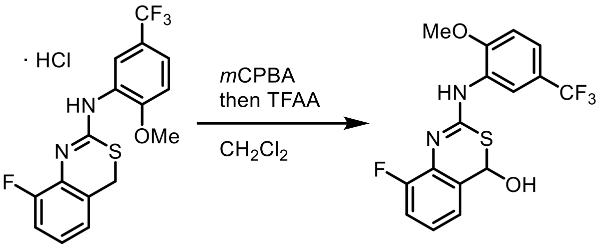

8 **7**

Isothiourea **8** (73.0 mg, 0.205 mmol, 1.00 equiv) was dissolved in CH_2Cl_2 (4.30 mL, 0.05M) and cooled to 0 °C. Then *m*CPBA (91.8 mg, 0.410 mmol, 2.00 equiv) was added at once. The reaction was stirred for 4 h until full conversion was observed. TFAA (31.8 μL, 0.225 mmol, 1.10 equiv) was added and after 10 min the reaction was quenched with sat. aq. $NaHCO_3$ (3.00 mL). The organic phase was separated and the aqueous phase was extracted with CH_2Cl_2 (3 x 5.00 mL). The combined organic phases were washed with brine (10.0 mL) and dried over Na_2SO_4. Filtration and evaporation of the solvent afforded the unpurified product (63.0 mg, 0.169 mmol, 83%).

Procedure 2:

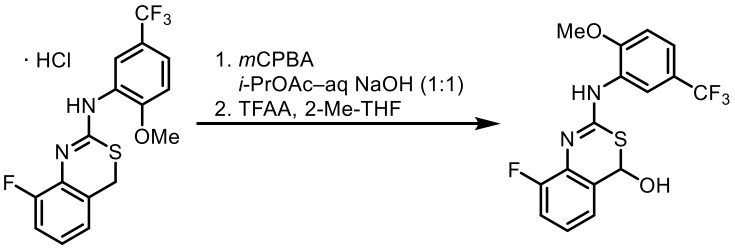

8 **7**

Isothiourea **8** (0.100 g, 0.255 mmol, 1.00 equiv) was dissolved in *i*-PrOAc (0.50 mL, 0.50M) and aq. NaOH (0.1M, 0.50 mL). Then *m*CPBA (70.3 mg, 0.306 mmol, 1.20 equiv) was added at once. The reaction was stirred for 6 h until full conversion was observed. The biphasic reaction mixture was separated and the organic solvent was evaporated. Then the residue was redissolved in 2-Me-THF (1.05 mL, 0.27M) and TFAA (39.5 μL, 0.280 mmol, 1.10 equiv) was added dropwise. The reaction was stirred for 30 min at 23 °C before addition of sat. aq. $NaHCO_3$ (2 mL) and separation of the organic phase. The organic phase was once washed with brine (2 mL) and then the solvent was removed *in vauo*. This two-pot procedure resulted in a yellow residue as product (73.5 mg, 0.197 mmol, 78%).

Procedure 3:

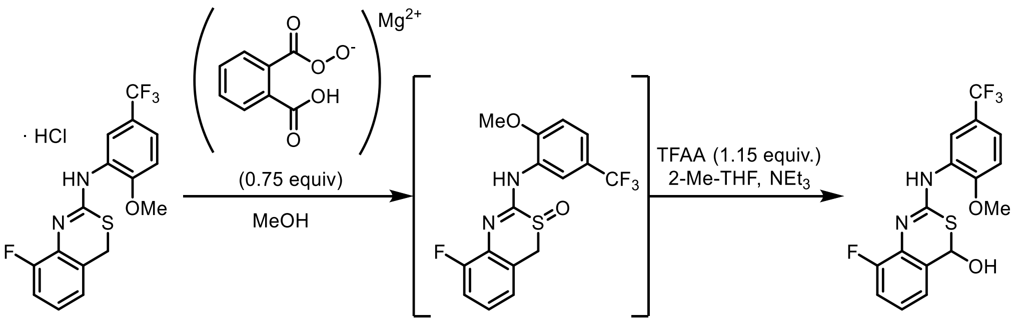

8 **9** **7**

The thiourea HCl salt **8** (20.0 g, 56.1 mmol, 1.00 equiv.) was dissolved in MeOH (100 mL) and Magnesium monoperoxy phthalic acid (26.0 g. 42.1 mmol, 0.75 equiv.) was added portionwise. The emulsion with off-white precipitate changed color to an orange heterogenous solution. The reaction was monitored via LC-MS. Upon completion of the reaction the solvent was removed, and the residue was washed with sat. aq. $NaHCO_3$ solution (100 mL) (careful gas evolution). After drying under vacuum the solid was characterized and identified as the sulfoxide (20.8 g, 55.9 mmol, 99% yield).

Some of the obtained material of **9** (7.00 g, 18.8 mmol, 1.00 equiv) was directly used without further purification and dissolved in 2-Me-THF (50.0 mL) which results in a heterogenous mixture. Et_3N (2.75 mL, 19.7 mmol, 1.05 equiv) was added dropwise before a solution of TFAA (2.74 mL, 19.7 mmol, 1.05 equiv) in 2-Me-THF (20.0 mL) was added dropwise. The reaction turned homogenous upon complete addition and the reaction was monitored via LC-MS and was complete after only 1-2 h (depending on the scale). Then the reaction was quenched by addition of sat. aq. $NaHCO_3$ solution. After stirring for 15 minutes at room temperature the phases were separated and the 2-Me-THF was removed under vacuo to obtain the desired product as yellow residue (6.82 g, 18.3 mmol, 97% yield).

Note: Characterization of the pure hemithioacetal ***7*** *turned out to be challenging due to different behavior of the material in basic or acidic medium and as well as depending on the solvent used for purification (e.g. transacetalization with MeOH). For example, it was observed on the LC-MS that under acidic conditions the hemithioacetal* ***7*** *tend to dimerize and results respectively in a mixture of at least 4 compounds (monomer, dimer, double-bond (isothiourea) isomers). The hemithioacetal can be further purified by flash column chromatography with Hex:Acetone (6:4) for analytical purposes.*

S-oxide (9):

^{1}H NMR (500 MHz, $CDCl_3$) δ 9.19 (d, J = 2.0 Hz, 1H), 9.07 (s, 1H), 7.39 (ddt, J = 8.5, 2.3, 0.9 Hz, 1H), 7.14 (ddt, J = 9.5, 8.4, 1.2 Hz, 1H), 7.05 – 6.93 (m, 3H), 4.66 (dq, J = 12.8, 1.1 Hz, 1H), 4.27 (dd, J = 12.9, 1.3 Hz, 1H), 4.01 (s, 3H).

^{13}C NMR (126 MHz, $CDCl_3$) δ 157.20, 155.20, 152.20, 150.71, 132.61 (d, *J* = 11.4 Hz), 127.68, 125.28 (d, *J* = 8.0 Hz), 124.92 (d, *J* = 3.7 Hz), 123.43 (q, *J* = 32.9 Hz), 121.52 (q, *J* = 3.9 Hz), 118.33 (d, *J* = 1.6 Hz), 117.70 (d, *J* = 19.9 Hz), 116.52 (q, *J* = 4.0 Hz), 109.63, 56.24, 51.04 (d, *J* = 3.2 Hz).

^{19}F NMR (471 MHz, $CDCl_3$) δ -61.86, -124.66 (dd, J = 9.9, 4.9 Hz).

IR (Diamond-ATR, neat): $\tilde{\nu}$ / cm^{-1} = 3298, 2135, 1639, 1600, 1540, 1493, 1474, 14443, 1346, 1322, 1272, 1211, 11178, 1161, 1149, 1127, 1079, 1065, 1042, 1024, 994, 928, 903, 814, 801, 782, 771, 729, 652, 622.

HRMS (ESI) calcd. for $C_{16}H_{13}F_4N_2O_2S$ [M+H$^+$]: 373.0628, found 373.0627.

TLC R_f (Hex:Acetone 6:4) = 0.48 (UV-active, Ceric ammonium molybdate)

Hemithioacetal (7):

^{1}H NMR (400 MHz, Acetone-*d₆*) δ 9.97 (s, 1H), 7.88 (dd, J = 2.3, 0.8 Hz, 1H), 7.74 – 7.68 (m, 1H), 7.33 – 7.29 (m, 1H), 7.26 (t, J = 1.3 Hz, 1H), 7.24 (d, J = 2.5 Hz, 1H), 7.20 – 7.13 (m, 1H), 6.22 (d, J = 8.6 Hz, 1H), 6.03 (d, J = 8.1 Hz, 1H), 3.86 (s, 3H).

^{13}C NMR (101 MHz, Acetone-d_6) δ 176.99, 158.15, 149.09 (d, J = 245.8 Hz), 133.32, 130.82 (q, J = 3.8 Hz), 127.91 (q, J = 231.57), 127.27 (q, J = 3.8 Hz), 124.60 (d, J = 1.9 Hz), 124.48 (d, J = 6.9 Hz), 123.83 (d, J = 3.7 Hz), 122.44 (q, J = 32.8 Hz), 116.32 (d, J = 17.5 Hz), 113.51, 80.84 (d, J = 2.4 Hz), 56.67.

^{19}F NMR (377 MHz, Acetone-d_6) δ –61.82, -134.27.

IR (Diamond-ATR, neat): $\tilde{\nu}$ / cm^{-1} = 2941, 1741, 1634, 1617, 1517, 1448, 1331, 1258, 1205, 1169, 1116, 1077, 1051, 1019, 952, 819, 799, 762, 721, 695, 652, 620.

HRMS (ESI) calcd. for $C_{16}H_{13}F_4N_2O_2S$ [M+H$^+$]: 373.0628, found 373.0632.

TLC R_f (Hex:Acetone 6:4) = 0.45 (UV-active, Ceric ammonium molybdate)

8-fluoro-3-(2-methoxy-5-(trifluoromethyl)phenyl)-2-(4-(3-methoxyphenyl)piperazin-1-yl)-3,4-dihydroquinazolin-4-ol (11)

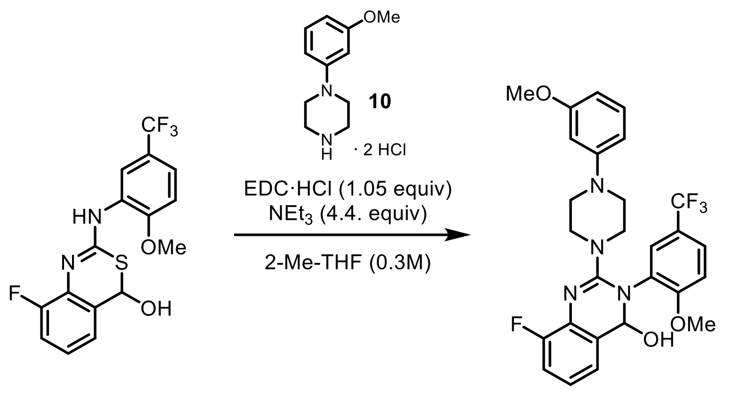

7 **11**

The hemiaminal **7** (7.00 g, 18.8 mmol, 1.00 equiv) was dissolved in 2-Me-THF (66.7 mL) to give a homogeneous solution. Piperizine·2·HCl **10** (5.24 g, 19.7 mmol, 1.05 equiv) was added along with Et_3N (11.5 mL, 82.7 mmol, 4.40 equiv). Then EDC·HCl (3.96 g, 20.7 mmol, 1.10 equiv) was added at once. The heterogenous reaction mixture was stirred for 24 h at room temperature and was monitored via LC-MS (reaction time depends on scale, smaller scale ca. 100-300 mg only needs 5-6 h). After completion of the reaction and full consumption of starting material (based on LC-MS) the reaction was quenched with 0.01 M aq. HCl and the organic phase was separated and dried under vacuum. Hemiaminal **11** was obtained as off-white solid (7.95 g, 15.0 mmol, 80% yield).

Note: Characterization of the pure hemiaminal turned out to be challenging due to different behavior of the material in basic or acidic medium and as well as depending on the solvent used for purification (e.g. transacetalization with MeOH). Nevertheless, as described above delivered sufficiently pure material for the subsequent steps. The hemiaminal can be further purified for analytical purposes by flash column chromatography with Hex:Acetone:PhMe (7.5:2.5:0.5).

^{1}H NMR (400 MHz, Acetone-d_6) δ 7.61 – 7.54 (m, 1H), 7.23 (dd, *J* = 8.0, 6.8 Hz, 1H), 7.19 – 7.13 (m, 2H), 7.09 – 7.03 (m, 2H), 7.01 – 6.93 (m, 1H), 6.49 – 6.44 (m, 1H), 6.43 – 6.35 (m, 2H), 5.98 – 5.94 (s, 1H), 3.97 – 3.92 (m, 3H), 3.72 (s, 3H), 3.50 (t, *J* = 5.0 Hz, 2H), 3.40 (t, *J* = 5.1 Hz, 2H), 2.92 (dddd, *J* = 38.3, 21.1, 13.3, 7.6 Hz, 4H).

^{13}C NMR (101 MHz, Acetone-d_6) δ 161.61, 157.31, 156.26 (d, J = 247.9 Hz), 153.68, 153.54, 138.49, 130.42, 129.76, 129.03, 126.12, 125.01 (q, J = 3.7 Hz), 123.92 (d, J = 4.1 Hz), 123.30 – 122.99 (m), 122.79 (d, J = 3.6 Hz), 115.63 (d, J = 19.6 Hz), 113.51, 109.41, 105.50, 103.05, 83.44, 56.77, 55.30, 49.31, 47.81.

^{19}F NMR (377 MHz, Acetone-d_6) δ -61.85, -130.11.

IR (Diamond-ATR, neat): $\tilde{\nu}$ / cm^{-1} = 2940, 2844, 1638, 1615, 1598, 1516, 1445, 1331, 1264, 1207, 1170,1079 1020, 982, 966, 919, 820, 732, 700, 620.

HRMS (ESI) calcd. for $C_{27}H_{27}F_4N_4O_3$ [M+H$^+$]: 531.2014, found 531.2015.

TLC R_f (Hex:Acetone 6:4) = 0.35 (UV-active, Ceric ammonium molybdate)

TBS-Me-silyl-Ketene-Acetal (12)

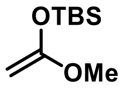

Prepared following the literature known procedure.[8]

^{1}H NMR (400 MHz, $CDCl_3$) δ 3.53 (s, 3H), 3.23 (d, J = 2.6 Hz, 1H), 3.10 (d, J = 2.6 Hz, 1H), 0.93 (s, 9H), 0.17 (s, 6H).

^{13}C NMR (101 MHz, $CDCl_3$) δ 162.48, 60.24, 55.16, 25.75, -4.57.

TBS-*i*-Pr-silyl-Ketene-Acetal (S12)

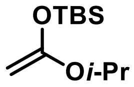

Prepared following the literature known procedure.[8]

^{1}H NMR (400 MHz, $CDCl_3$) δ 4.19 (hept, J = 6.1 Hz, 1H), 3.27 (d, J = 2.3 Hz, 1H), 3.11 – 3.06 (m, 1H), 1.25 (d, J = 6.1 Hz, 7H), 0.93 (s, 9H), 0.17 (s, 6H).

^{13}C NMR (101 MHz, $CDCl_3$) δ 159.87, 69.97, 61.75, 26.08, 21.75, -4.37.

TMS-Me-silyl-Ketene-Acetal (S13)

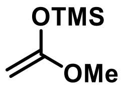

Prepared following the literature known procedure. Product mixture of O-TMS and C–TMS was obtained. Assumed to be inconsequential for the Mukaiyama–Mannich reaction.[9]

^{1}H NMR (400 MHz, $CDCl_3$) δ 3.55 (s, 1H), 3.21 (d, J = 2.8 Hz, 0H), 3.10 (d, J = 2.8 Hz, 0H), 0.23 (s, 3H).

^{13}C NMR (101 MHz, $CDCl_3$) δ 162.23, 60.02, 55.22, 0.10.

TES-Me-silyl-Ketene-Acetal (S14)

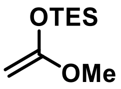

^{1}H NMR (400 MHz, $CDCl_3$) δ 3.51 (s, 3H), 3.20 (d, J = 2.7 Hz, 1H), 3.05 (d, J = 2.7 Hz, 1H), 1.01 – 0.84 (m, 9H), 0.67 (m, 6H).

^{13}C NMR (101 MHz, $CDCl_3$) δ 162.37, 59.54, 55.04, 4.94, 3.61.

Prepared following adjusted conditions from the literature. [9] TESCl was used instead of TMSCl. Product mixture of O-TMS and C–TMS was obtained. Assumed to be inconsequential for the Mukaiyama–Mannich reaction.

methyl (S)-2-(8-fluoro-3-(2-methoxy-5-(trifluoromethyl)phenyl)-2-(4-(3-methoxyphenyl)piperazin-1-yl)-3,4-dihydroquinazolin-4-yl)acetate (13)

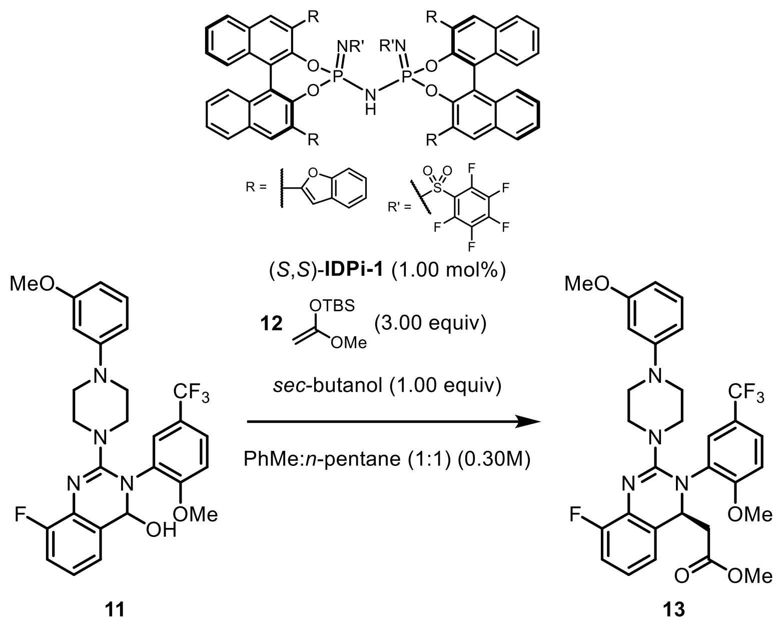

The hemiaminal **11** (300 mg, 0.511 mmol, 1.00 equiv) was dissolved in PhMe:*n*-pentane (2.10 mL, 2.10 mL 1:1) and TBS-Methyl-silyl-ketene-acetal **12** (335 µL, 1.53 mmol, 3.00 equiv) and *sec*-butanol (47.0 µl, 0.511 mmol, 1.00 equiv) were added. Then organocatalyst **IDPi-1** (8.18 mg, 5.10 µmol, 1.00 mol%, 3.00 wt%) was added and the reaction was stirred until full consumption of the starting material observed by LC-MS or TLC, up to 5 days. The reaction was quenched with a few drops of MeOH and evaporated *in vacuo* for NMR and chiral HPLC analysis. The unpurified material can be used for the following steps including either direct saponification or recrystallization. For analytic purposes and yield determination the product can be further purified by flash column chromatography with Hex:Acetone:PhMe (7.5:2.5:0.5). The product was obtained as a sticky colorless solid in 75% yield (225 mg, 0.384 mmol).

Note: *For analytic purposes the product can be further purified by flash column chromatography with Hex:Acetone:PhMe (7.5:2.5:0.5). This allows for purification of the desired product as well as the recovery of the organocatalyst (up to 92% recovery) that can be reused for the above-mentioned reaction after re-acidification with DOWEX as described above.*

Recrystallization was performed following the protocol reported by Merck:[10]

Methyl ester **13** (225 mg, 0.384 mmol, 80% ee) was dissolved in PhMe (3.00 mL) and the solution was heated to 45 °C. Then a solution of (*S*,*S*)-di-*p*-toloyltartaric acid ((–)-DTTA, 30.0 mg, 76.7 µmol, 0.20 equiv) in EtOAc (0.09 mL, 0.875M) was added and the mixture seeded with

Methyl ester **13** DTTA salt. The mixture was stirred for 2 h at 45 °C during which time a thin slurry formed. Further (–)-DTTA (118 mg, 0.307 mmol, 0.8 equiv) in EtOAc (0.35 mL, 0.875M) was then added via syringe pump over 12 h. The slurry was allowed to cool to RT and stirred for 3 h before being filtered. The cake was washed with EtOAc (3 × 0.5 mL) and air-dried to afford methyl Ester **13** DTTA salt (327 mg, 0.357 mmol, 93% yield, >99% ee) as a crystalline white solid.

^{1}H NMR (400 MHz, MeCN-d_3) δ 7.47 (ddd, J = 8.5, 2.4, 0.8 Hz, 1H), 7.27 – 7.16 (m, 3H), 7.15 – 7.07 (m, 2H), 7.01 (ddd, J = 11.1, 8.1, 1.4 Hz, 1H), 6.88 (ddd, J = 8.1, 7.5, 4.8 Hz, 1H), 6.80 (dd, J = 7.5, 1.4 Hz, 1H), 6.44 (ddd, J = 8.3, 2.2, 0.9 Hz, 1H), 6.40 – 6.37 (m, 2H), 4.94 – 4.89 (m, 1H), 3.85 (s, 3H), 3.72 (s, 3H), 3.65 (s, 3H), 3.59 – 3.36 (m, 4H), 2.97 (ddd, J = 11.9, 7.0, 3.4 Hz, 2H), 2.91 (dd, J = 14.4, 7.1 Hz, 1H), 2.83 (s, 2H), 2.56 (dd, J = 14.4, 7.5 Hz, 1H).

^{13}C NMR (101 MHz, MeCN-d_3) δ 170.92, 160.68, 156.60 (d, J = 26.2 Hz), 153.77 (d, J = 50.4 Hz), 152.80, 137.93, 134.29, 132.72 (d, J = 11.1 Hz), 129.78, 129.31 (d, J = 3.0 Hz), 128.96, 128.25, 125.58, 125.29, 123.86 (q, J = 4.0 Hz), 122.83 (t, J = 5.6 Hz), 122.33 (d, J = 7.7 Hz), 120.84 (d, J = 3.4 Hz), 114.63 (d, J = 20.0 Hz), 112.96, 108.68, 104.74, 102.26, 58.97, 55.92, 54.71, 51.34, 48.52, 45.81, 39.67,

^{19}F NMR (377 MHz, Acetonitrile-d_3) δ -61.92, -131.12.

IR (Diamond-ATR, neat): $\tilde{\nu}$ / cm^{-1} = 2934, 2844, 1735, 1583, 1558, 1516, 1496, 1453, 1435, 1330, 1274, 1252, 1202, 1168, 1139, 1119, 1080, 1046, 1139, 993, 982, 963, 820, 753, 691, 621.

HRMS (ESI) calcd. for $C_{30}H_{31}F_4N_4O_4$ [M+H$^+$]: 587.2276, found 587.2275.

TLC Hex:Acetone:PhMe (7.2:2.2:0.5*)* R_f = 0.25 (UV-active, Ceric ammonium molybdate no heating required)

Optical rotation α_D = +124.417±1.638 (25 °C, c = 0.3)

Racemic sample of 13:

ReproSil chiral-OM, 5 µm (250 x 4.6 mm), instrument method: 65% H_2O+0.1% formic acid 35% MeCN isocratic_15min run.

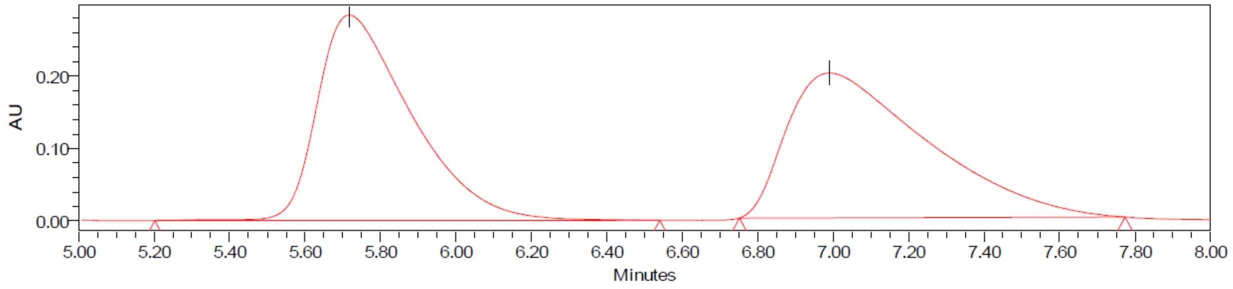

Processed Channel: PDA 254.0 nm

	Processed Channel	Retention Time (min)	Area	% Area	Height
1	PDA 254.0 nm	5.716	4891737	48.73	283708
2	PDA 254.0 nm	6.991	5146958	51.27	199973

90:10 er sample of 13:

ReproSil chiral-OM, 5 µm (250 x 4.6 mm), instrument method: 65% H_2O+0.1% formic acid 35% MeCN isocratic_15min run.

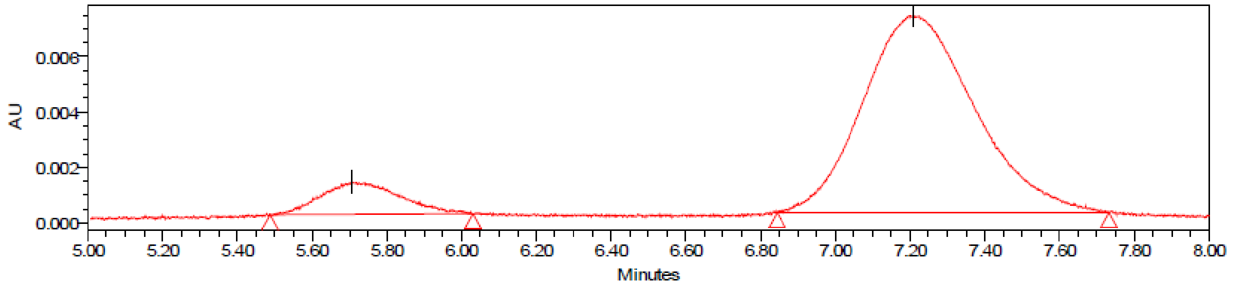

Processed Channel: PDA 254.0 nm

	Processed Channel	Retention Time (min)	Area	% Area	Height
1	PDA 254.0 nm	5.707	16704	10.19	1147
2	PDA 254.0 nm	7.209	147224	89.81	7137

97:3 er sample of 13:

ReproSil chiral-OM, 5 µm (250 x 4.6 mm), instrument method: 65% H_2O+0.1% formic acid 35% MeCN isocratic_15min run.

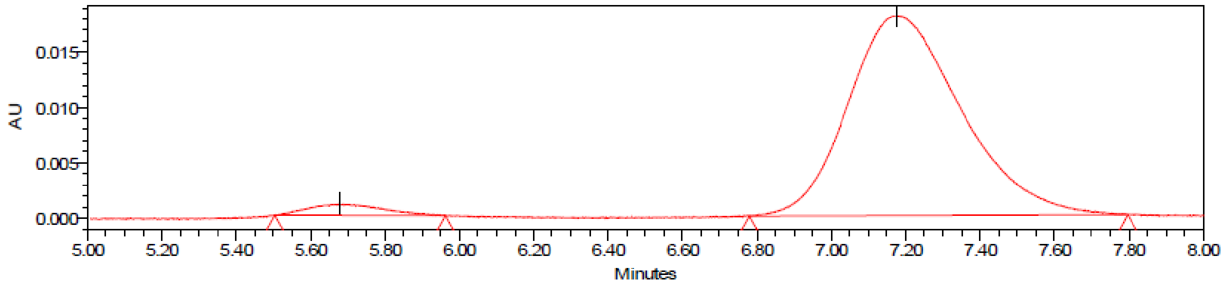

Processed Channel: PDA 254.0 nm

	Processed Channel	Retention Time (min)	Area	% Area	Height
1	PDA 254.0 nm	5.678	13734	3.47	1000
2	PDA 254.0 nm	7.174	381575	96.53	18098

>99% ee sample of 13:

ReproSil chiral-OM, 5 µm (250 x 4.6 mm), instrument method: 65% H_2O+0.1% formic acid 35% MeCN isocratic_15min run.

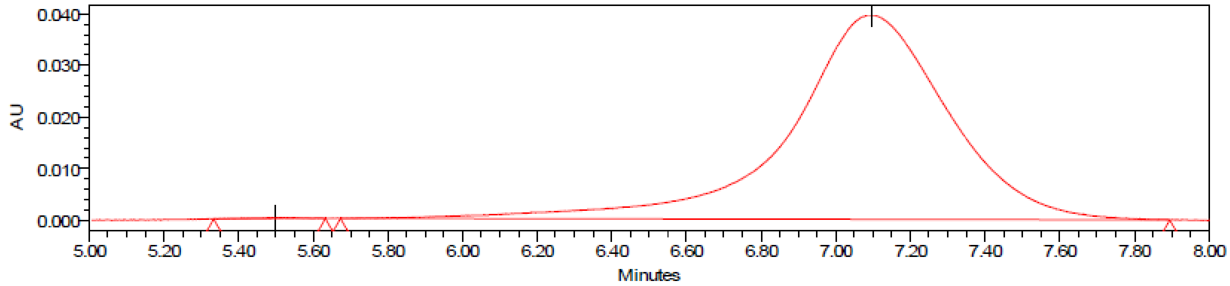

Processed Channel: PDA 254.0 nm

	Processed Channel	Retention Time (min)	Area	% Area	Height
1	PDA 254.0 nm	5.498	1513	0.13	177
2	PDA 254.0 nm	7.097	1160385	99.87	39527

Letermovir (1): Following the procedure of Merck:[10]

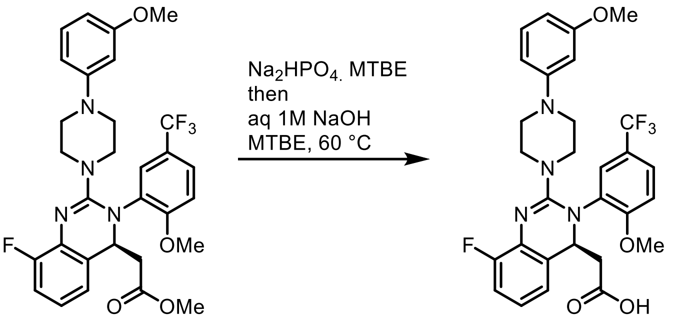

Methyl Ester **13** (–)-DTTA salt (327 mg, 0.357 mmol, 1.00 equiv) was slurried in MTBE (7.60 mL) and aq. 0.5M Na_2HPO_4 (1.78 mL, 0.792 mmol, 2.50 equiv) added. The mixture was stirred for 2 h before the aqueous layer was separated and aq. 1M NaOH (1.43 mL, 1.43 mmol, 4.00 equiv) was added. The stirred mixture was then heated to 60 °C, with continuous removal of MTBE by distillation. The resulting aqueous reaction mixture was stirred at 60 °C for 5 h before being cooled to RT. H_2O (2 mL) and MTBE (2 mL) were then added, and the organic phase was separated (discarded). The aqueous phase was carefully acidified (pH 5–6) with aq. 1M HCl. The organic phase was separated and concentrated. The residue was taken up in acetone (as little as possible) and slowly added to H_2O (3.2 mL), and the ensuing slurry was stirred for a further 1 h before being filtered. The cake was washed with H_2O (3 x 2 mL) and air-dried to provide letermovir **1** (192 mg, 0.335 mmol, 94% yield) as an amorphous white powder.

^{1}H NMR (400 MHz, DMSO-d_6) δ 12.46 (s, 1H), 7.57 – 7.50 (m, 1H), 7.39 (s, 1H), 7.23 (d, J = 8.7 Hz, 1H), 7.11 – 6.99 (m, 2H), 6.87 (q, J = 2.8 Hz, 2H), 6.48 – 6.42 (m, 1H), 6.41 – 6.32 (m, 2H), 4.88 (t, J = 7.3 Hz, 1H), 3.80 (s, 3H), 3.68 (s, 3H), 3.35 (s, 6H), 2.94 (ddd, J = 10.8, 6.9, 3.3 Hz, 2H), 2.79 (dd, J = 14.8, 7.4 Hz, 1H), 2.44 (dd, J = 14.8, 7.3 Hz, 1H).

^{13}C NMR (101 MHz, DMSO-d_6) δ 171.74, 160.14, 156.54, 154.48 (d, J = 246.5 Hz), 153.15, 152.17, 134.09, 132.24, 129.61, 124.04 (q, J = 271.3 Hz), 123.89, 122.40, 122.12, 121.39 (q, J = 32.8 Hz), 120.84, 114.52 (d, J = 19.3 Hz), 113.36, 108.27, 104.64, 101.89, 58.93, 56.32, 54.85, 47.87, 45.59, 39.93.

^{19}F NMR (377 MHz, DMSO-d_6) δ -59.94, -129.64.

IR (Diamond-ATR, neat): $\tilde{\nu}$ / cm^{-1} = 2925, 2853, 1718, 1599, 1583, 1556, 1516, 1496, 1452, 1330, 1274, 1251, 1202, 1170, 1143, 1120, 1080, 1046, 1024, 985, 965, 821, 754.

HRMS (ESI) calcd. for $C_{29}H_{29}F_4N_4O_4$ [M+H$^+$]: 573.2119, found 573.2120.

TLC DCM:MeOH (9:1) R_f = 0.17 (UV-active, Ceric ammonium molybdate no heating required)

Optical rotation α_D = +219.409±2.046 (25 °C, c = 1)

	Literature (Gong et al.)[11]	Literature (Merck)[10]	This work
**^{1}H NMR (DMSO-*d_6*)**	7.56 (dd, J = 8.8, 2.7 Hz, 1H)<br>7.41 (brs, 1H)<br>7.25 (d, J = 8.3 Hz, 1H)<br>7.09 (t, J = 8.1 Hz, 1H)<br>7.12 – 7.01 (m, 1H)<br>6.94 – 6.83 (m, 2H)<br>6.47 (dd, J = 8.1, 2.6 Hz, 1H)<br>6.41 (t, J = 2.3 Hz, 1H)<br>6.38 (dd, J = 7.8, 2.4 Hz, 1H),<br>4.89 (t, J = 7.3 Hz, 1H)<br>3.81 (brs, 3H), 3.70 (s, 3H)<br>3.53−3.40 (m, 4H)<br>2.98−2.83 (m, 4H)<br>2.79 (dd, J = 14.7, 7.5 Hz, 1H)<br>2.45 (dd, J = 14.7, 7.3 Hz, 1H)	7.52 (dd, J = 8.7, 1.7 Hz,1H)<br>7.40 (brs, 1H), 7.21 (m, 1H)<br>7.07 (t, J = 8.2 Hz, 1H)<br>7.04 (m, 1H), 6.87 (m, 2H)<br>6.44 (dd, J = 8.2, 1.9 Hz, 1H)<br>6.40(t, J = 2.3 Hz, 1H)<br>6.36 (dd, J = 8.0, 2.0 Hz, 1H)<br>4.89 (t, J =7.2 Hz, 1H)<br>3.80 (brs, 3H), 3.68 (s, 3H)<br>3.39−3.48 (m, 4H)<br>2.82−2.95 (m, 4H)<br>2.80 (dd, J = 14.8, 7.4 Hz, 1H)<br>2.46 (dd, J= 14.9, 7.4 Hz, 1H)	12.46 (s, 1H)<br>7.57 – 7.50 (m, 1H)<br>7.39 (s, 1H)<br>7.23 (d, J = 8.7 Hz, 1H)<br>7.11 – 6.99 (m, 2H)<br>6.87 (q, J = 2.8 Hz, 2H)<br>6.48 – 6.42 (m, 1H)<br>6.41 – 6.32 (m, 2H)<br>4.88 (t, J = 7.3 Hz, 1H)<br>3.80 (s, 3H)<br>3.68 (s, 3H)<br>3.35 (s, 6H),<br>2.94 (ddd, J = 10.8, 6.9, 3.3 Hz, 2H)<br>2.79 (dd, J = 14.8, 7.4 Hz, 1H)<br>2.44 (dd, J = 14.8, 7.3 Hz, 1H)
**^{13}C NMR (DMSO-*d_6*)**	171.86, 160.15, 156.57<br>154.55 (d, J_{CF} = 246.1 Hz)<br>153.18, 152.22 134.19<br>132.3 (d, J_{CF} = 11.6 Hz)<br>129.65,<br>124.08 (q, J_{CF} = 272.7Hz)<br>123.86 (q, J_{CF} = 3.9 Hz)<br>122.40<br>122.07 (q, J_{CF} = 7.3 Hz)<br>121.4 (q, J_{CF} = 30.0 Hz)<br>123.87<br>114.5 (d, J_{CF} = 19.0 Hz)113.36, 108.31 104.67<br>101.90, 59.01, 56.35, 54.89<br>47.92, 45.58, 40.0	171.8, 160.2, 156.5<br>154.6 (d, JCF = 246.3 Hz)<br>153.2, 152.2 134.2<br>132.3 (d, J_{CF} = 11.2 Hz)<br>129.6<br>124.1 (q, J_{CF} = 271.3Hz)<br>123.8 (q, J_{CF} = 3.7 Hz)<br>122.4 122.1 (q, J_{CF} = 7.1 Hz)<br>121.4 (q, J_{CF} = 29.2 Hz)<br>120.8 114.5 (d, J_{CF} = 19.5 Hz)<br>113.3, 108.3, 104.6, 101.9, 59.0, 56.3, 54.8, 47.9, 45.6, 40.0	171.74, 160.14, 156.54<br>154.48 (d, J_{CF} = 246.5 Hz)<br>153.15, 152.17, 134.09, 132.24, 129.61<br>124.04 (q, J_{CF} = 271.3 Hz)<br>123.89, 122.40, 122.12<br>121.39 (q, J_{CF} = 32.8 Hz)<br>120.84, 114.52 (d, J_{CF} = 19.3 Hz)<br>113.36, 108.27, 104.64, 101.89, 58.93, 56.32, 54.85, 47.87, 45.59, 39.93.

7. NMR Spectra

^{1}H NMR (500 MHz)

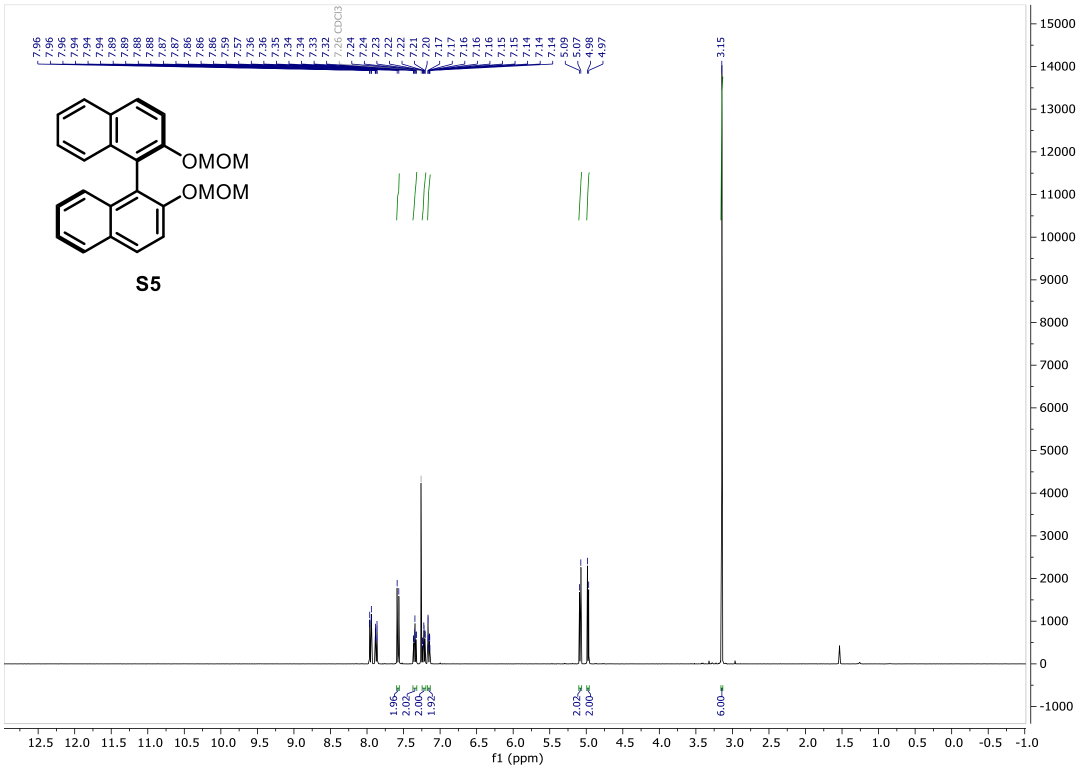

^{13}C NMR (126 MHz)

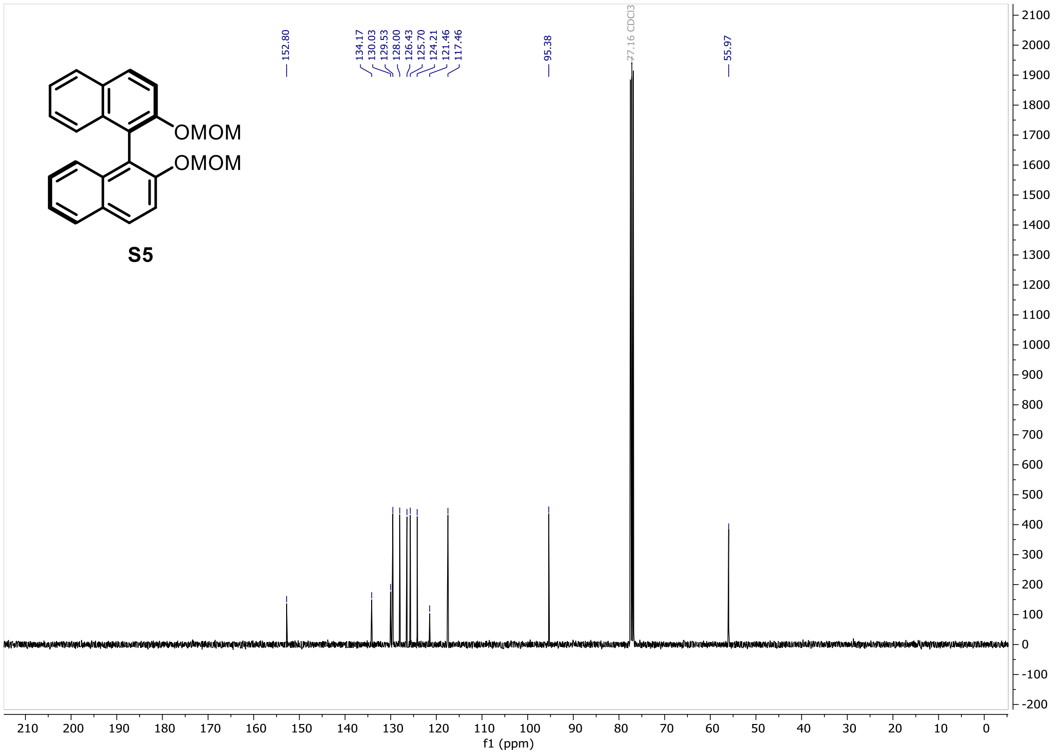

^{1}H NMR (400 MHz)

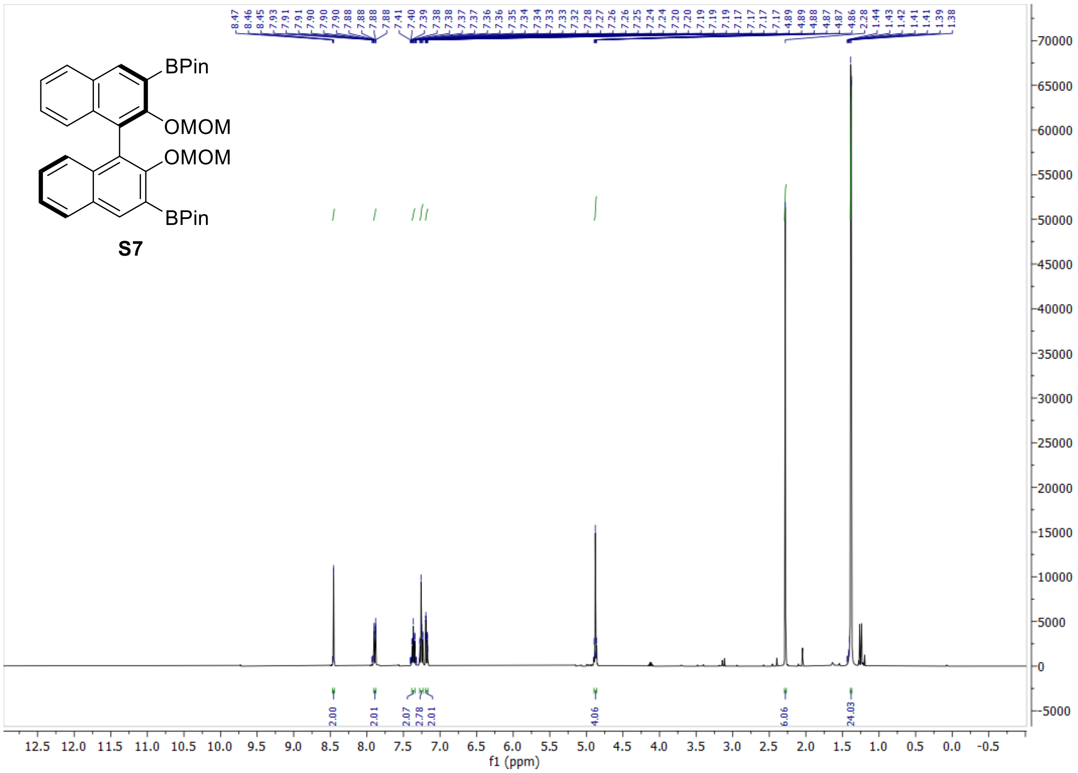

^{13}C NMR (101 MHz)

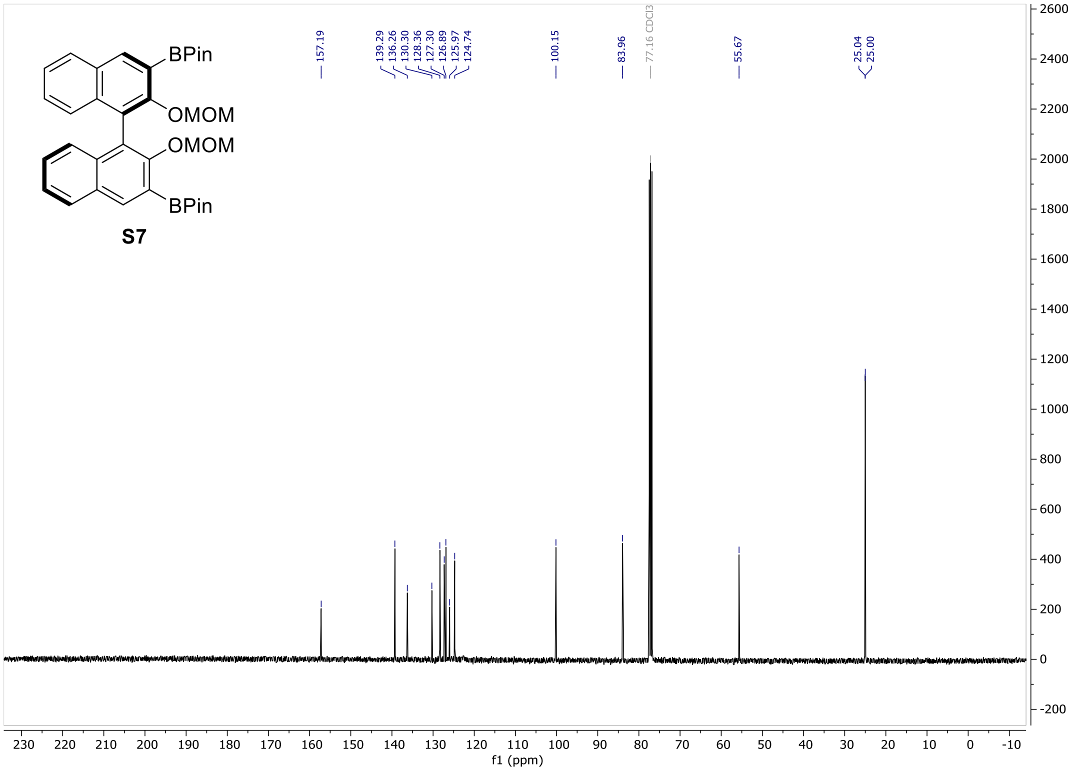

^{1}H NMR (400 MHz)

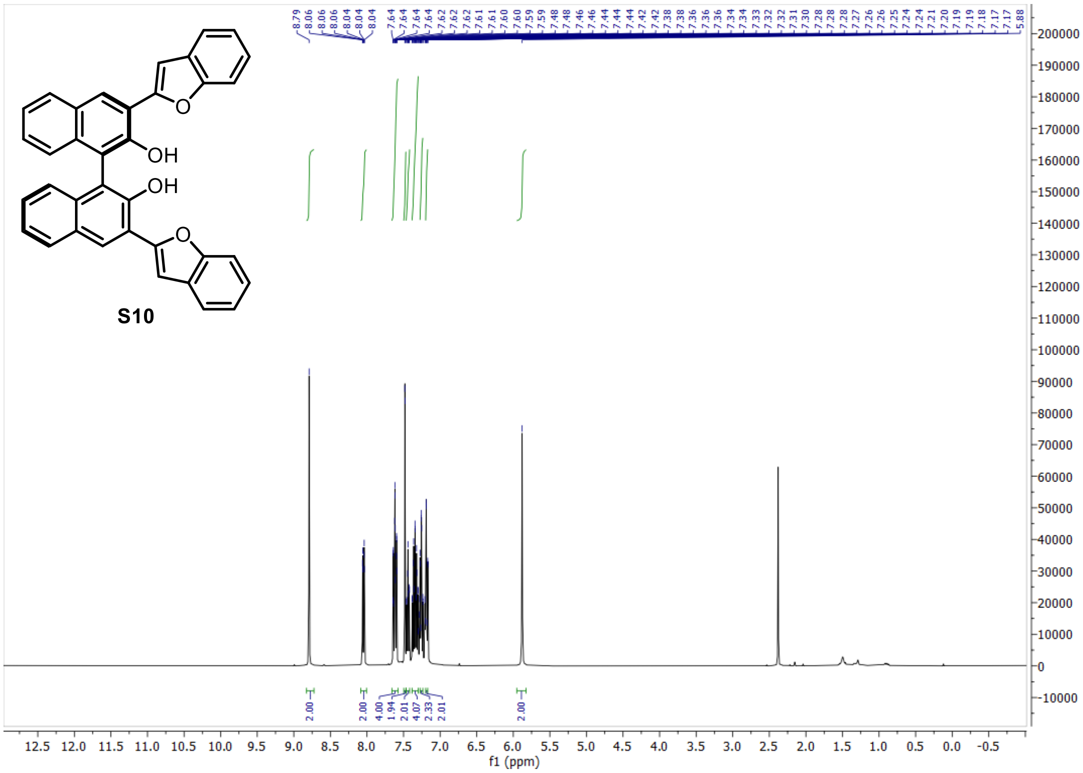

^{13}C NMR (101 MHz)

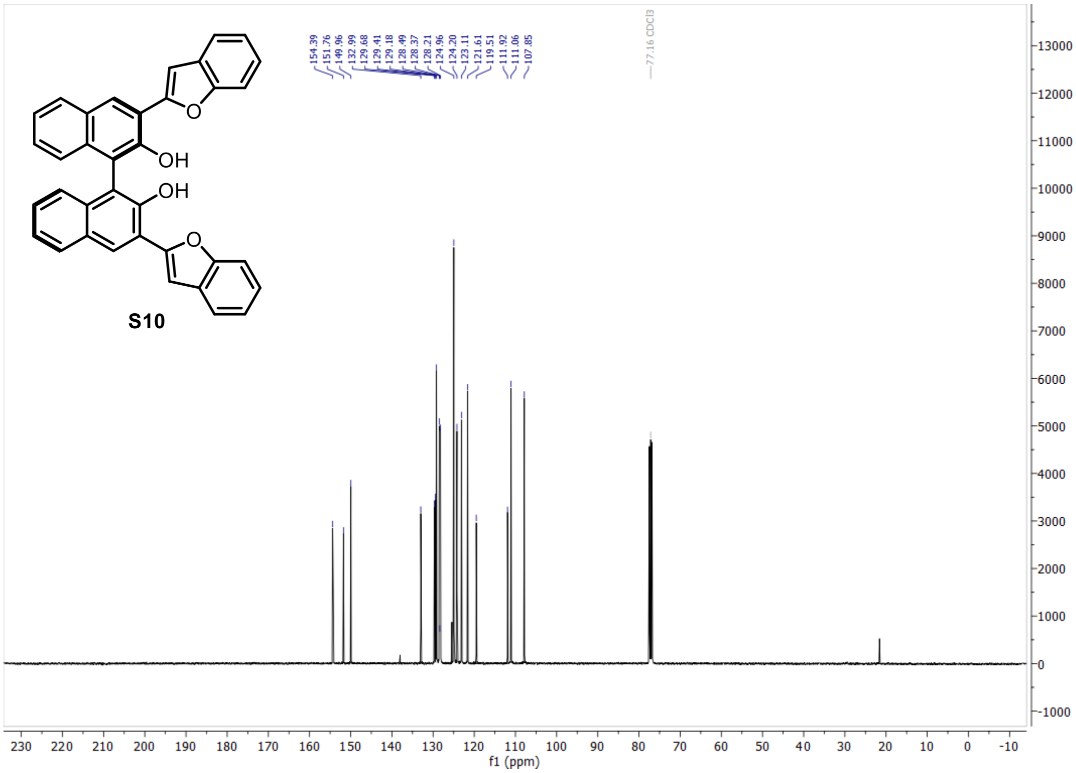

^{1}H NMR (400 MHz)

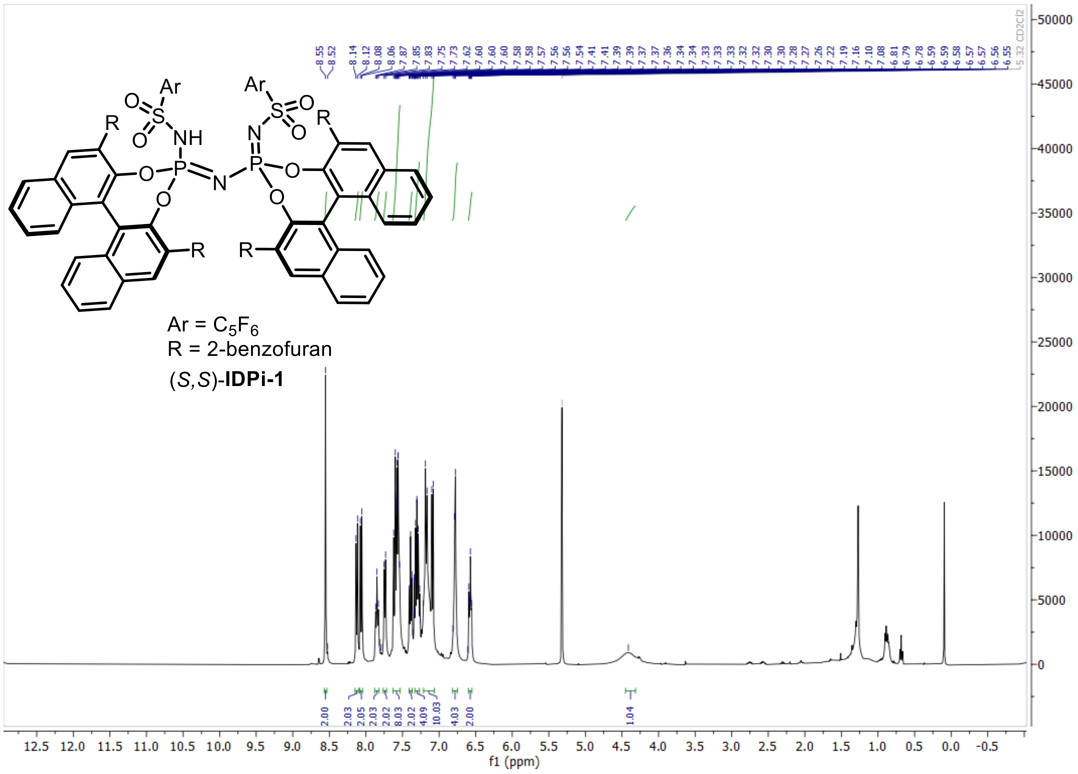

^{13}C NMR (101 MHz)

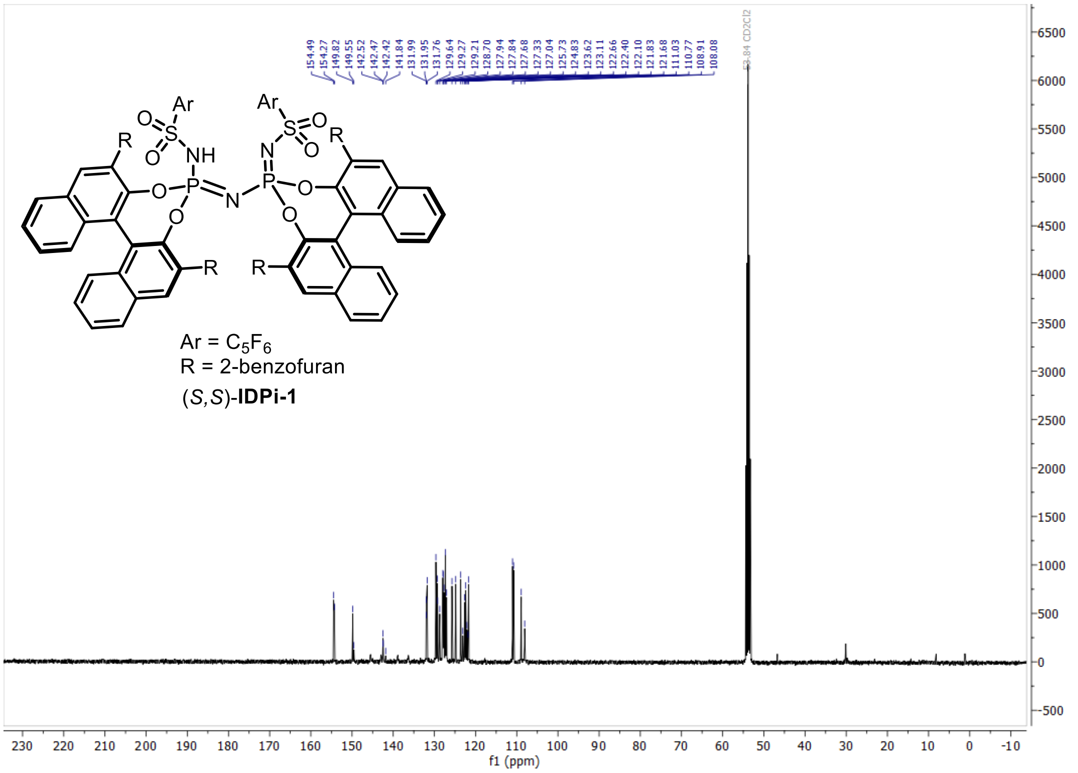

^{1}H NMR (400 MHz)

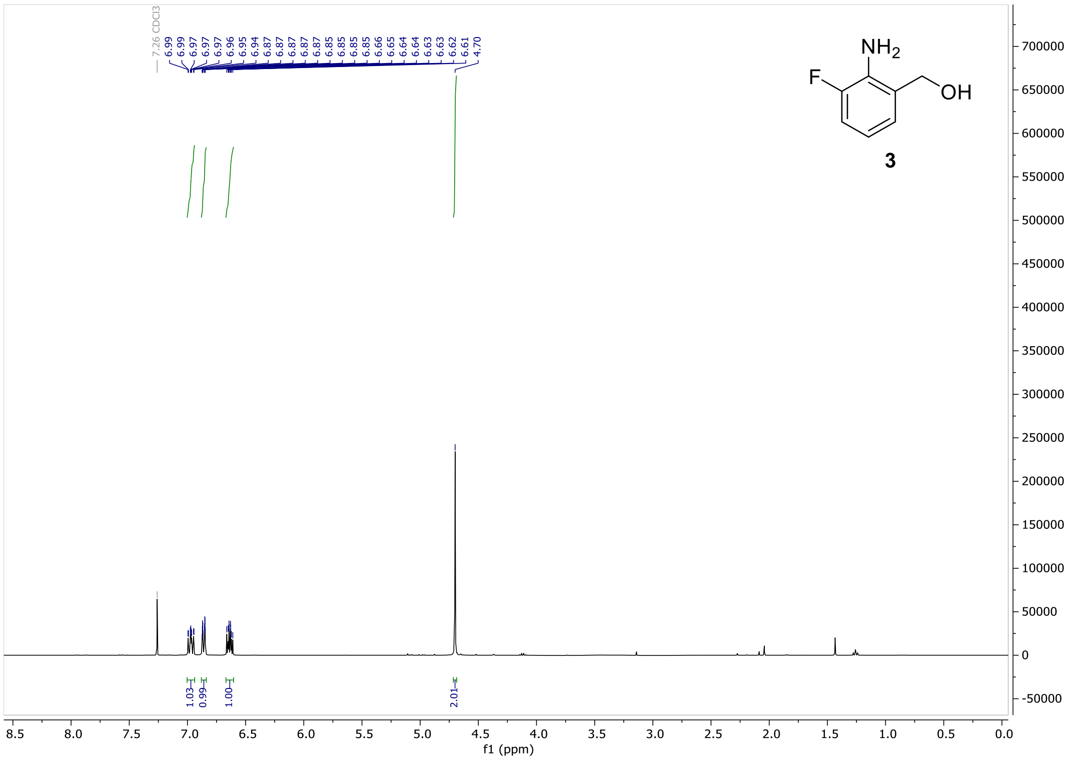

^{13}C NMR (101 MHz)

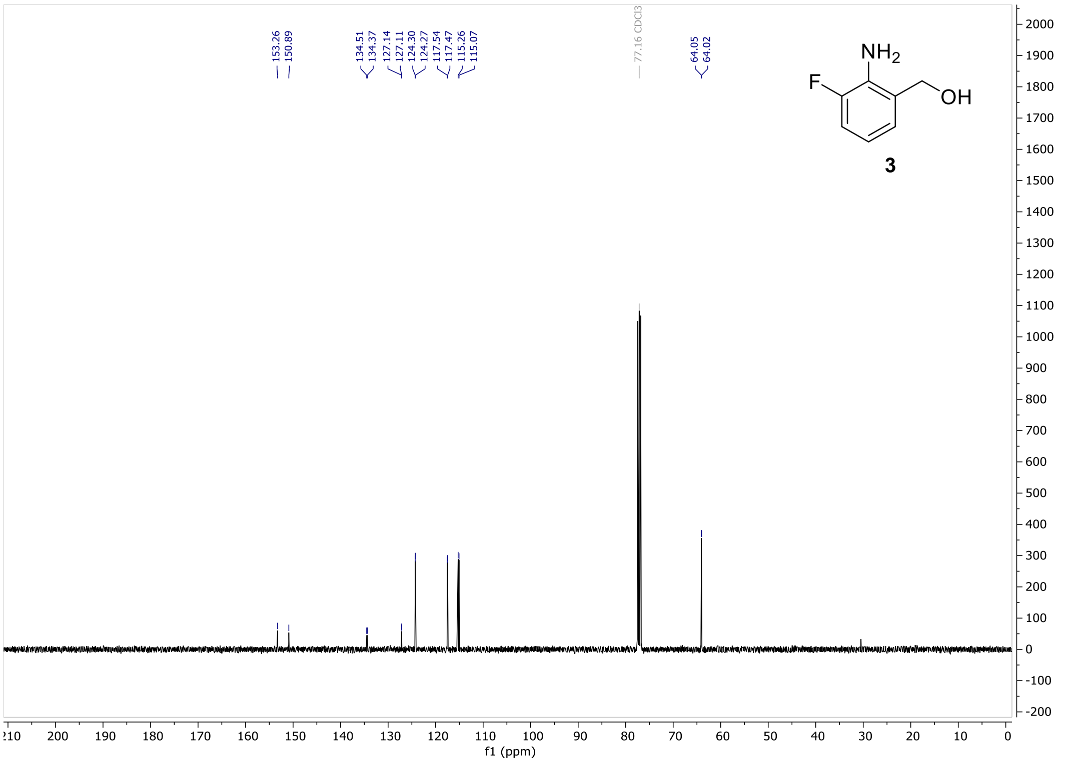

^{19}F NMR (377 MHz)

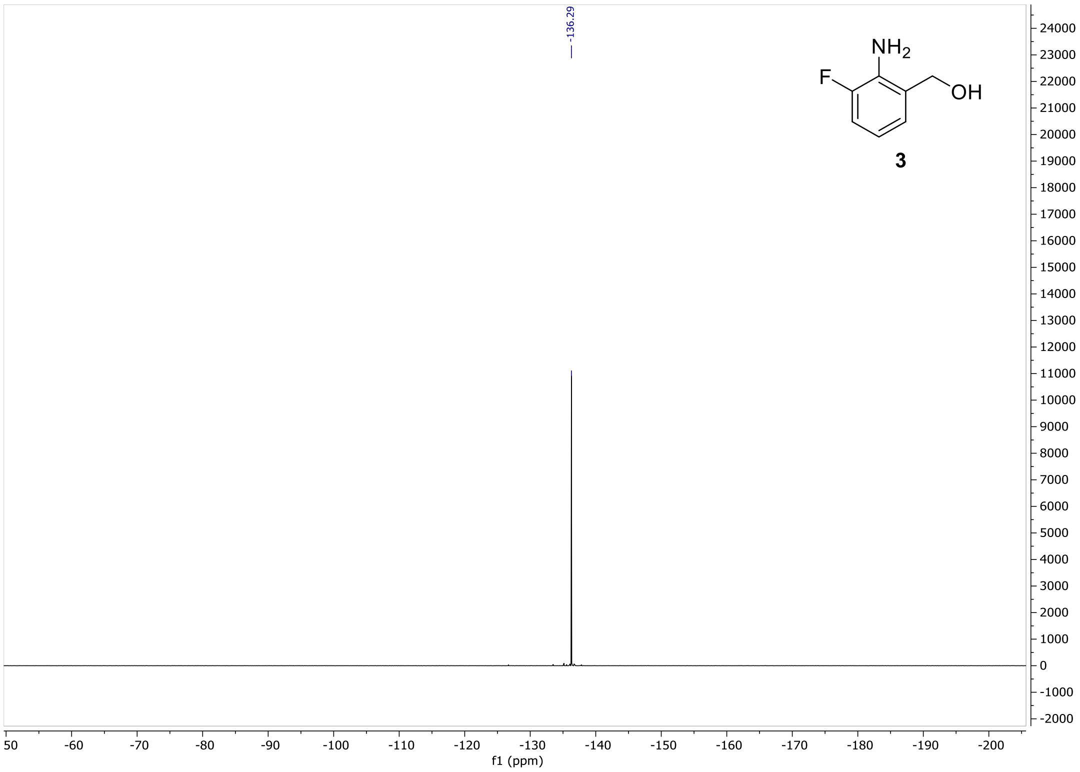

^{1}H NMR (500 MHz)

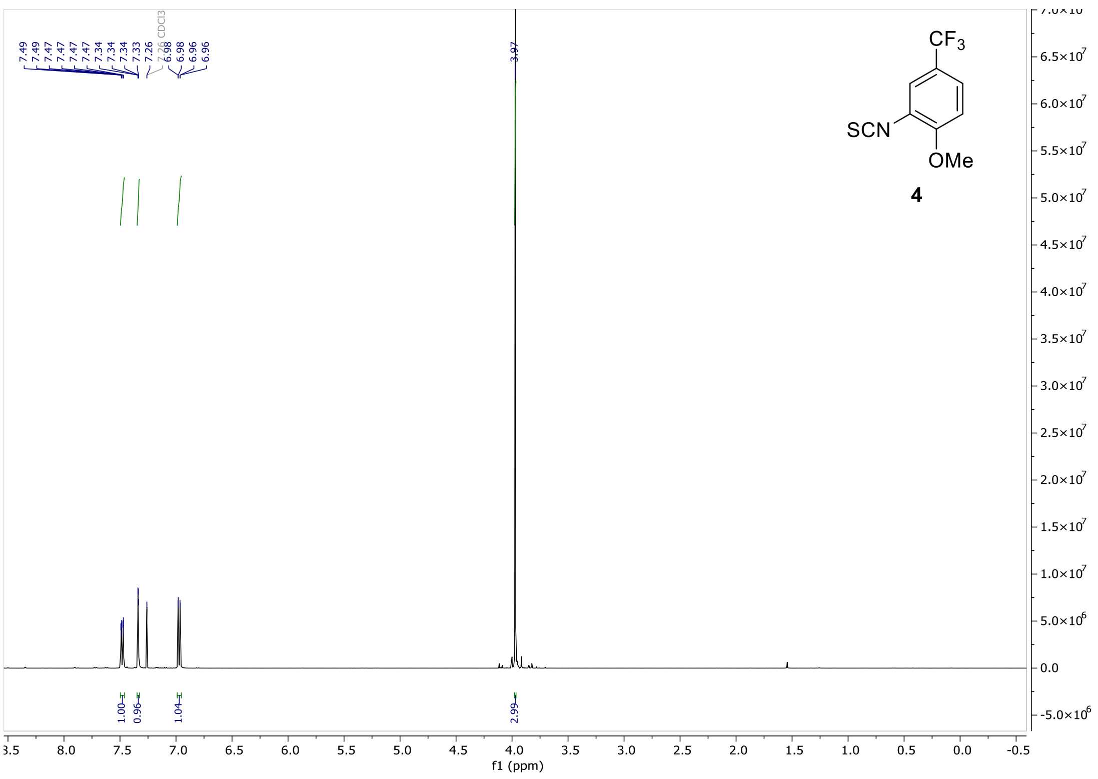

^{13}C NMR (126 MHz)

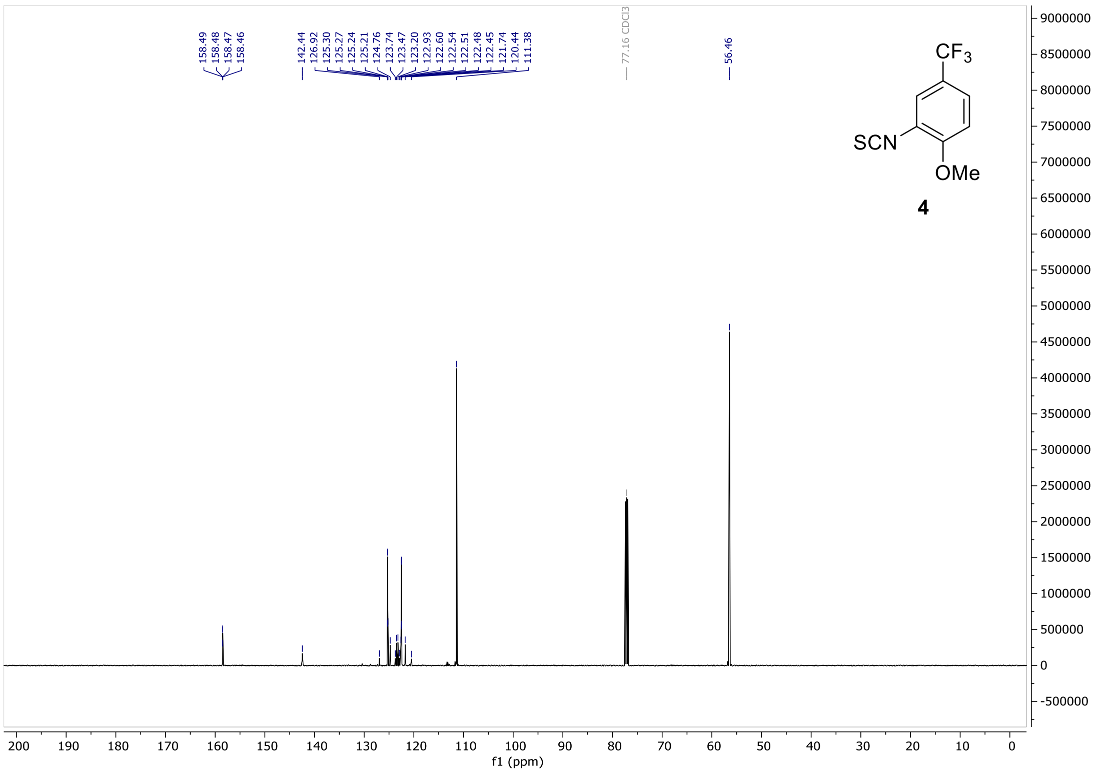

^{19}F NMR (471 MHz)

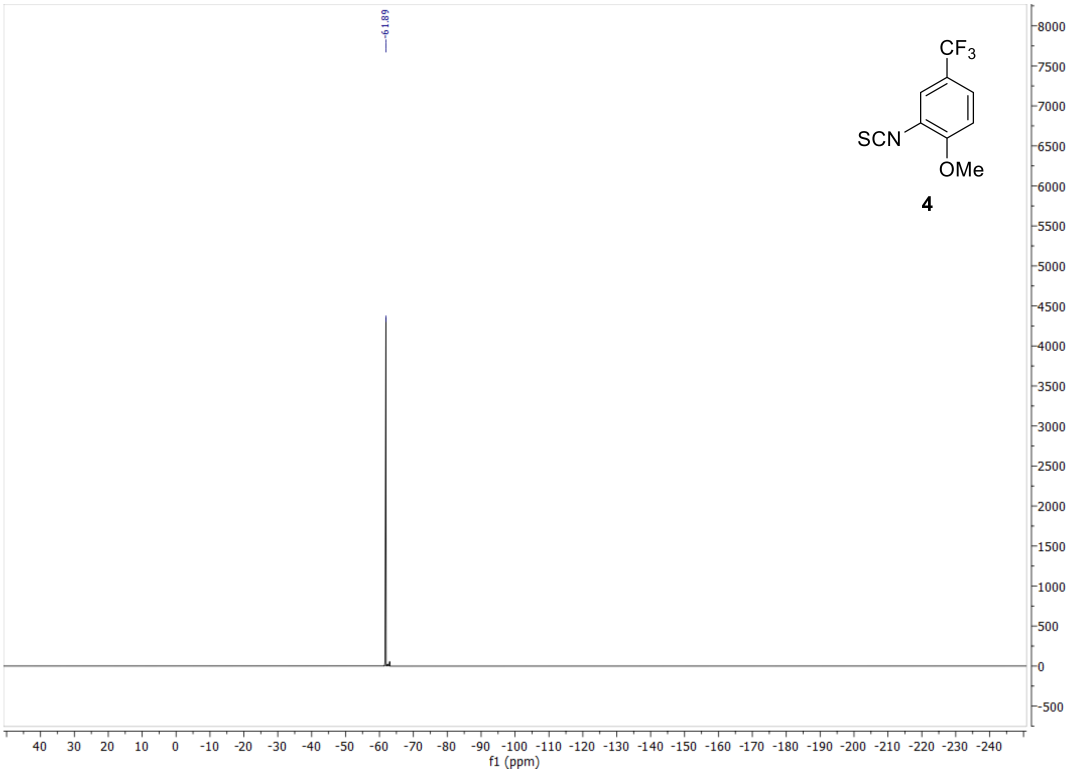

^{1}**H NMR** (500 MHz)

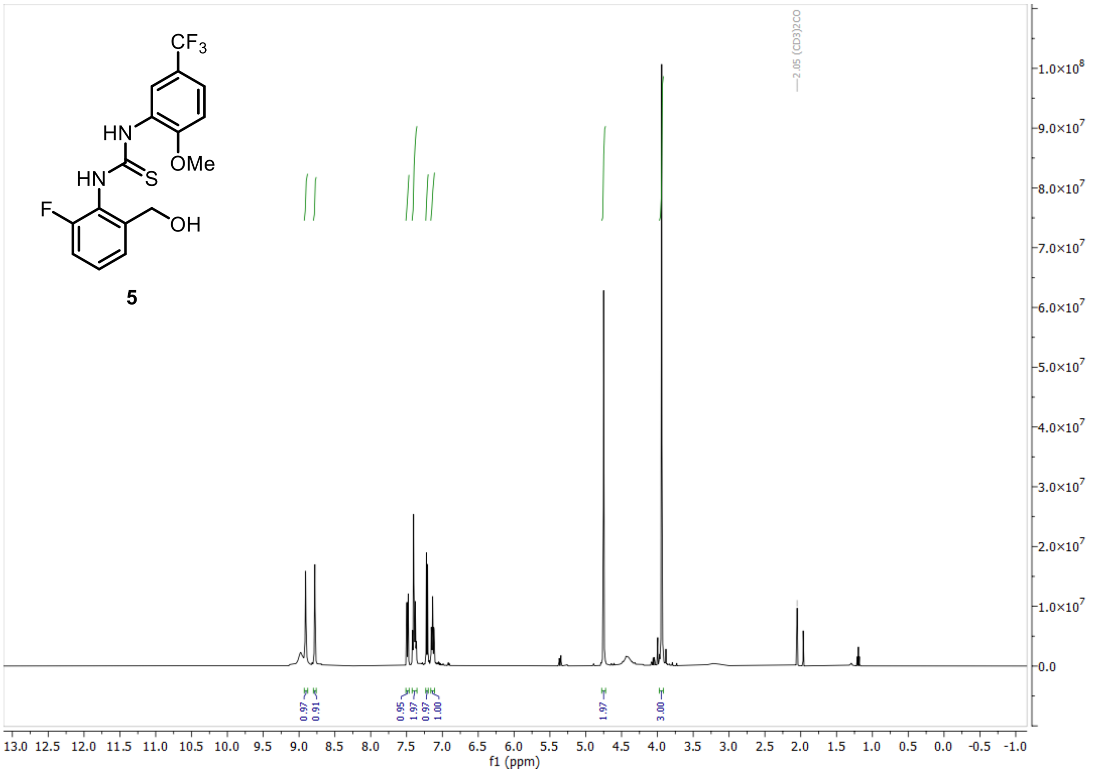

^{13}**C NMR** (126 MHz)

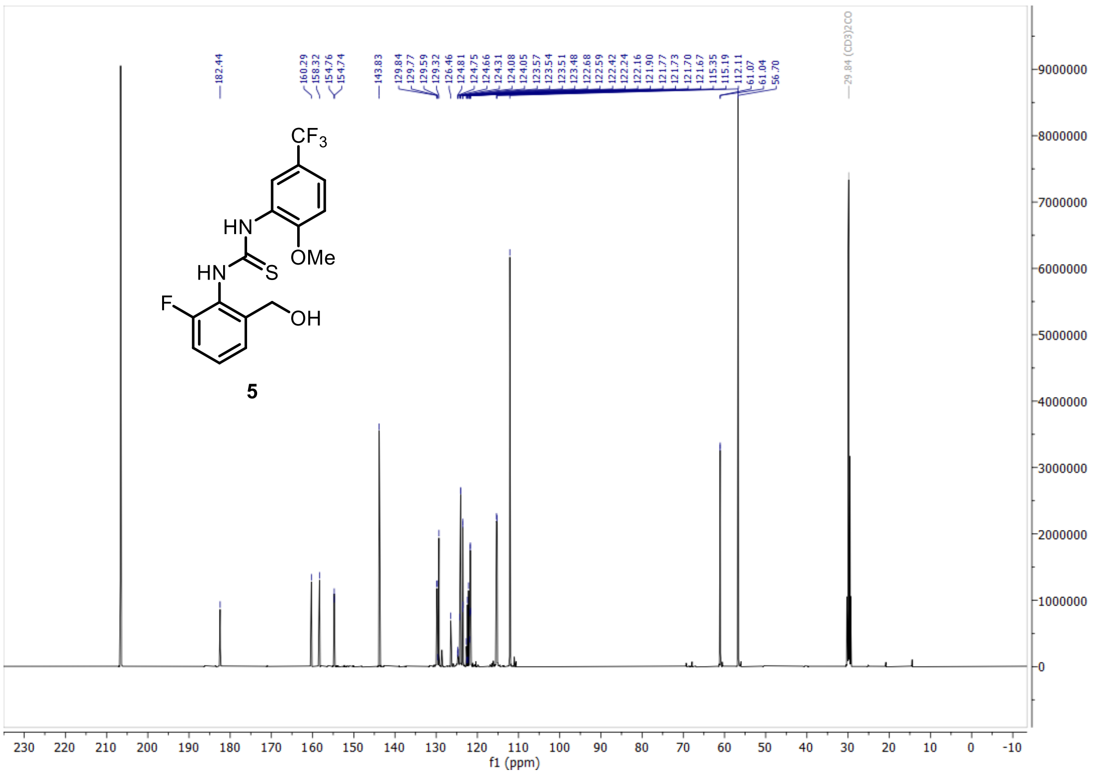

^{19}F NMR (471 MHz)

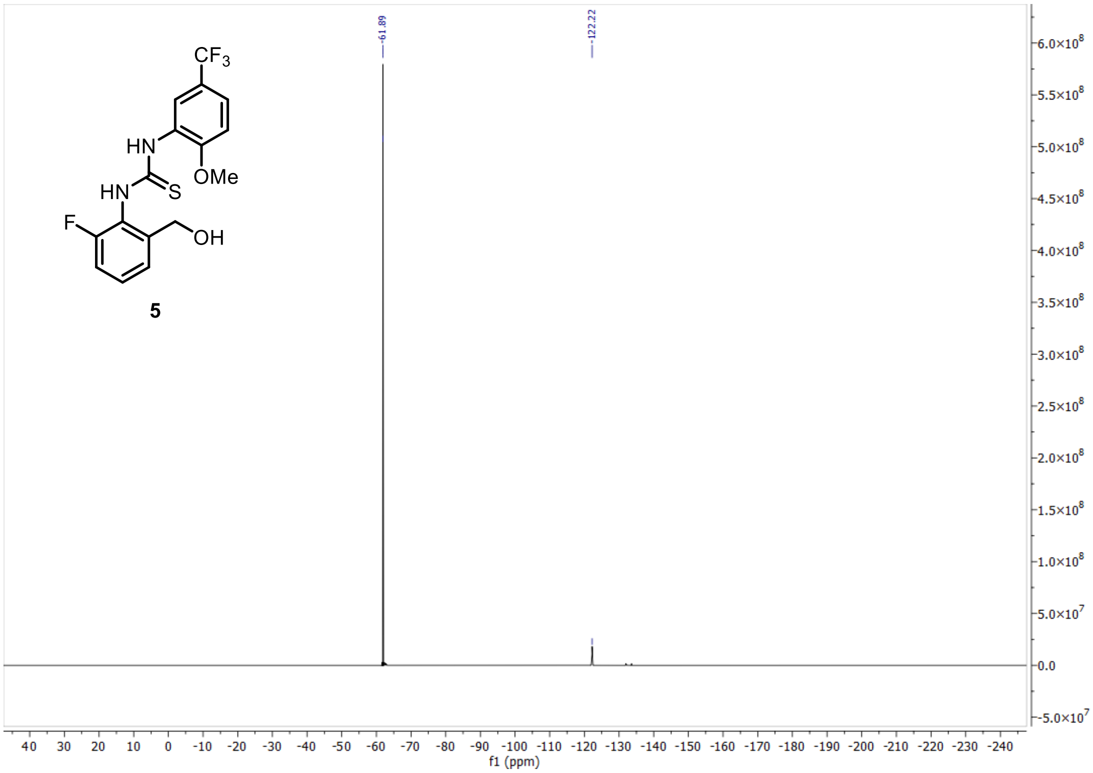

^{1}H NMR (500 MHz)

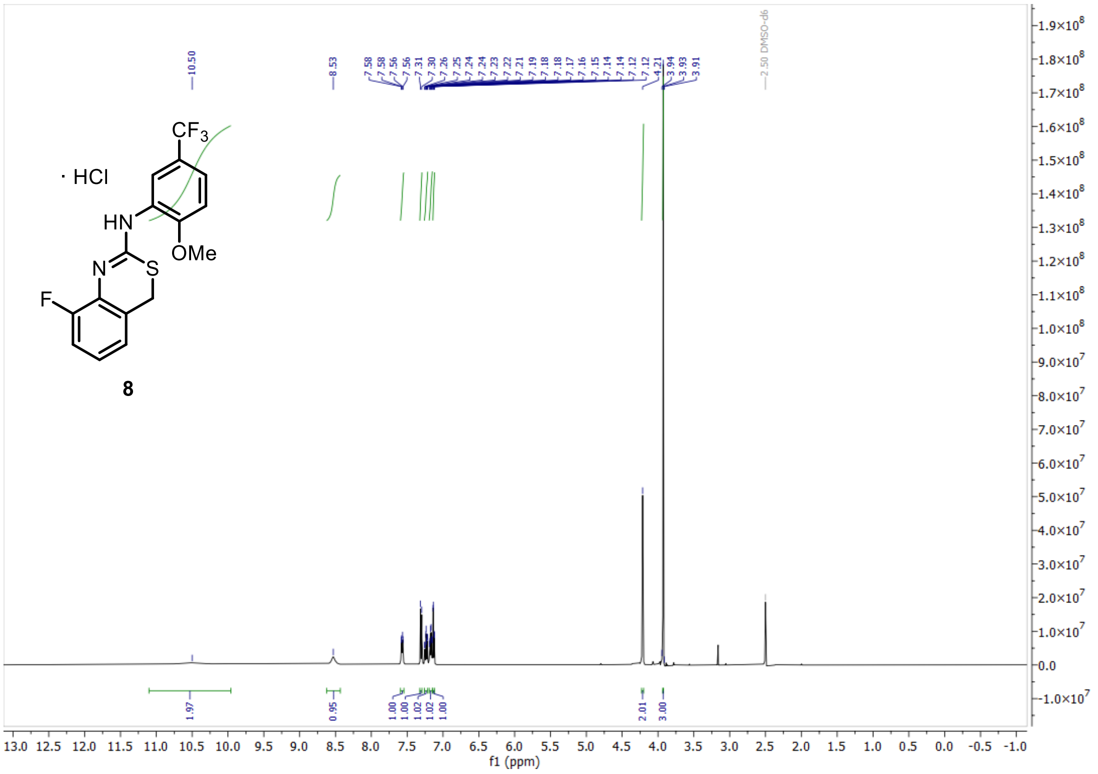

^{13}C NMR (126 MHz)

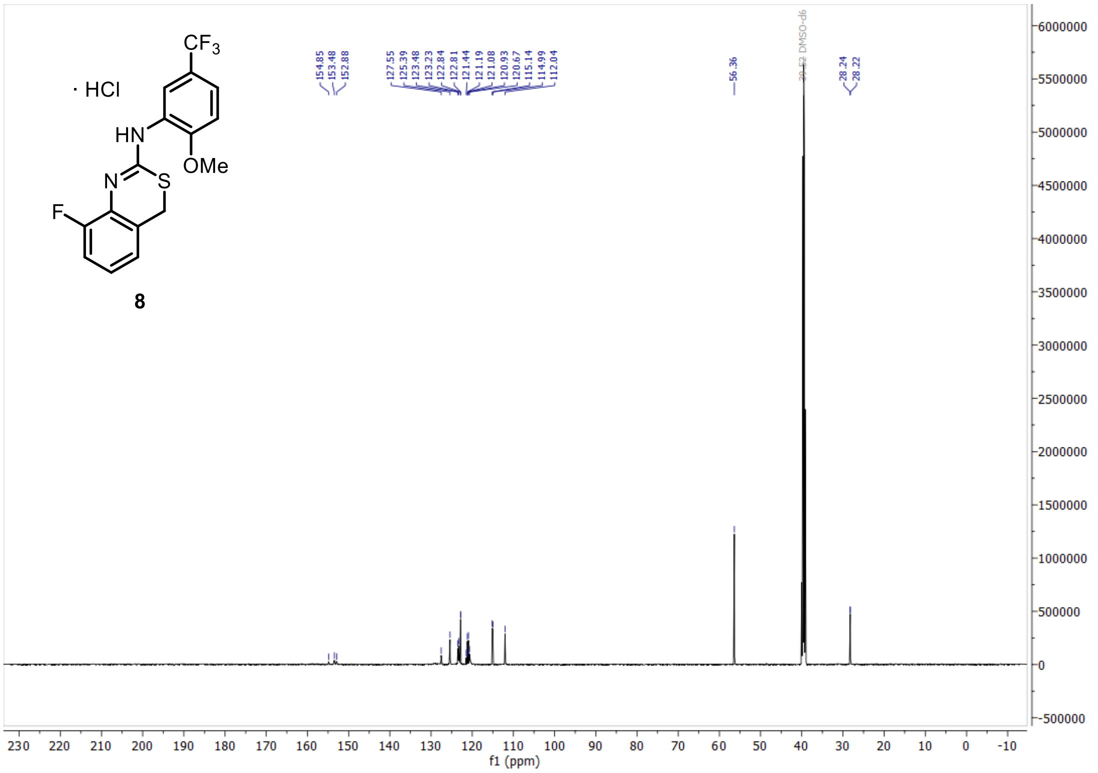

^{19}F NMR (471 MHz)

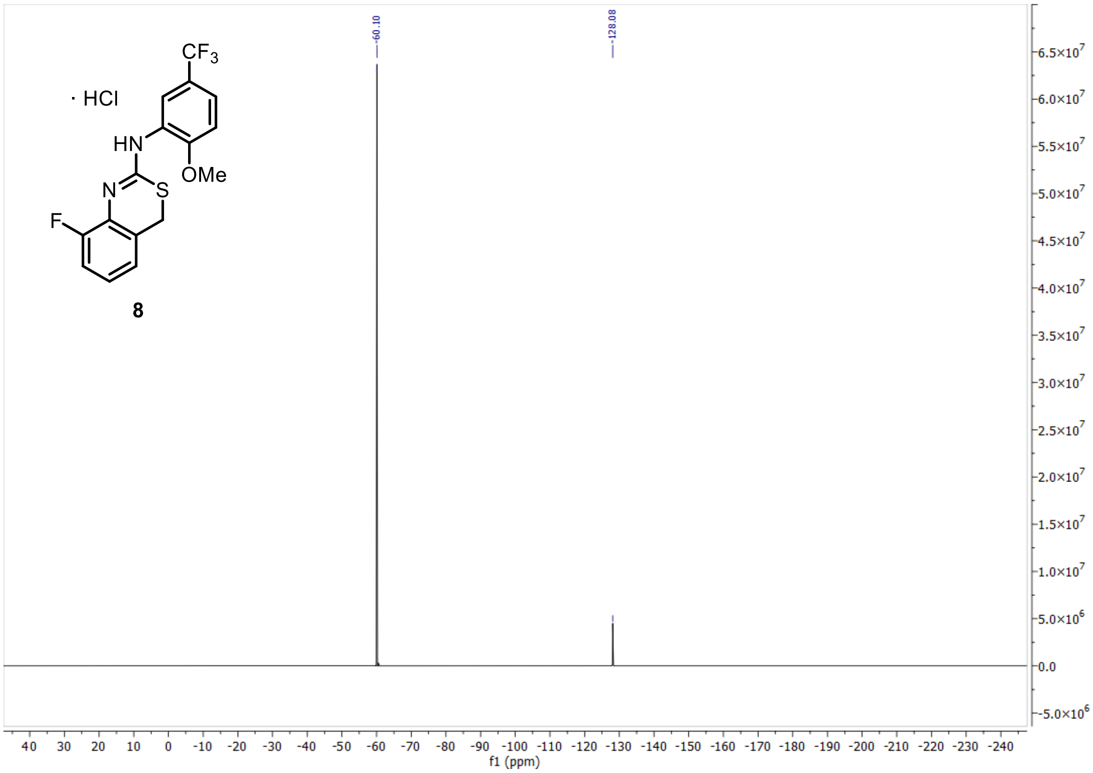

^{1}H NMR (500 MHz)

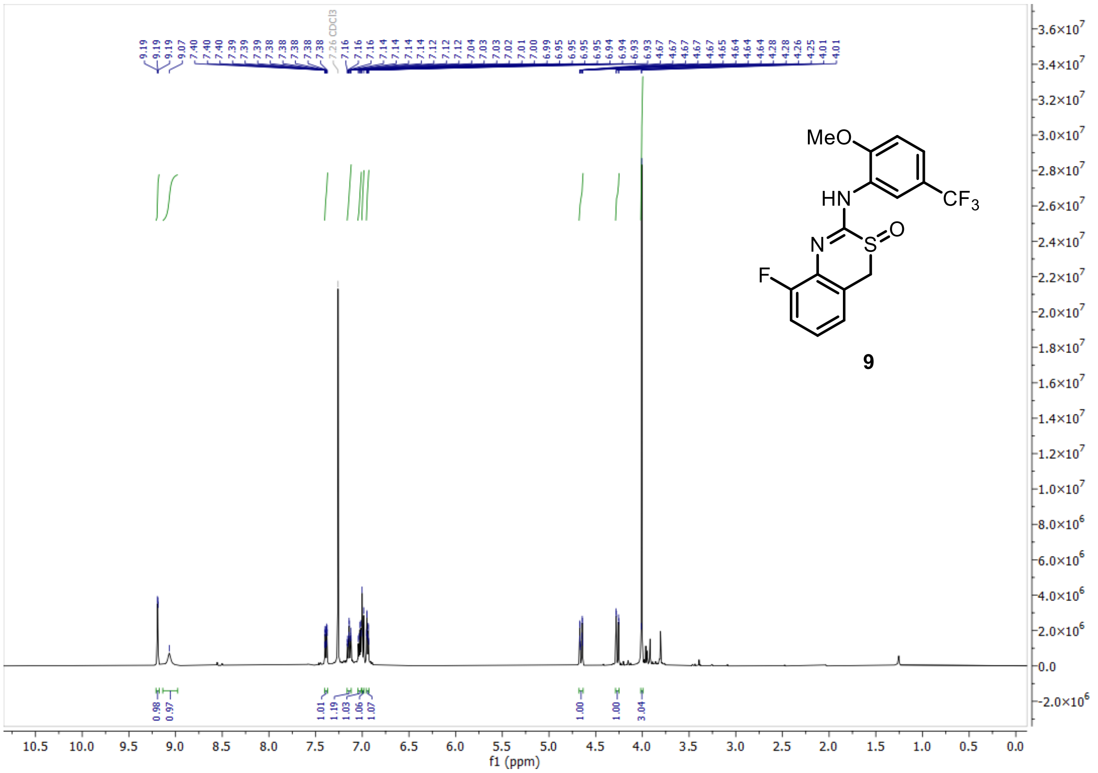

^{13}C NMR (126 MHz)

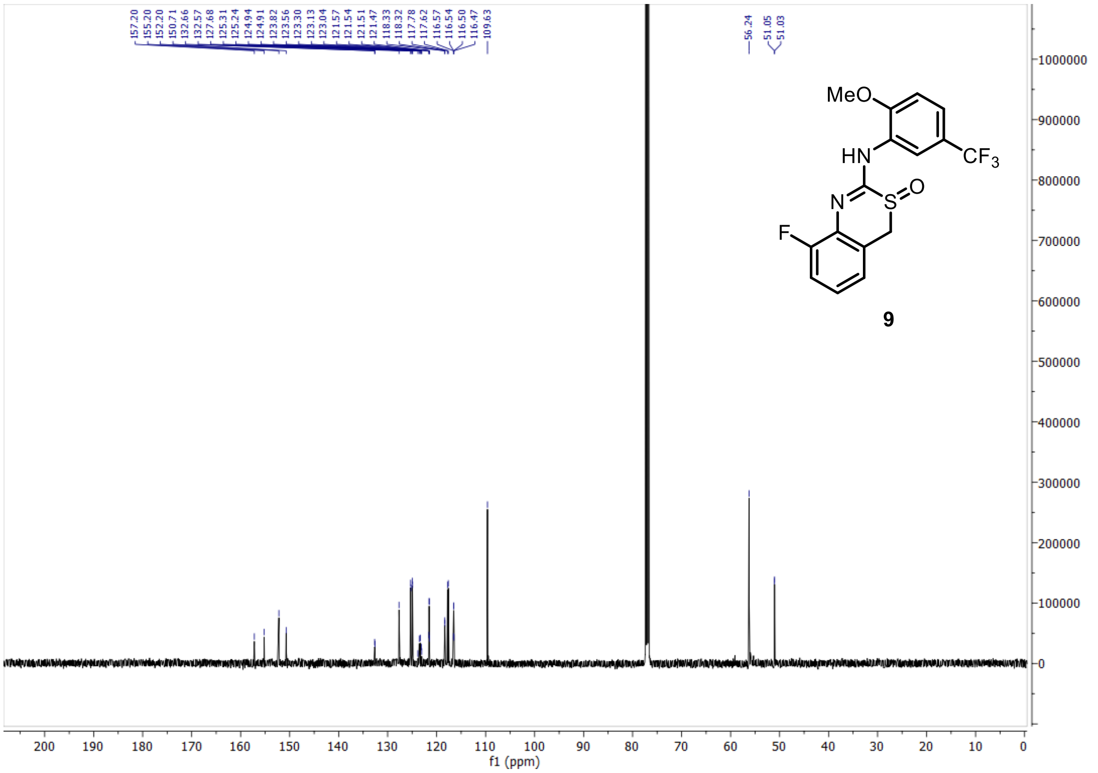

^{19}F NMR (471 MHz)

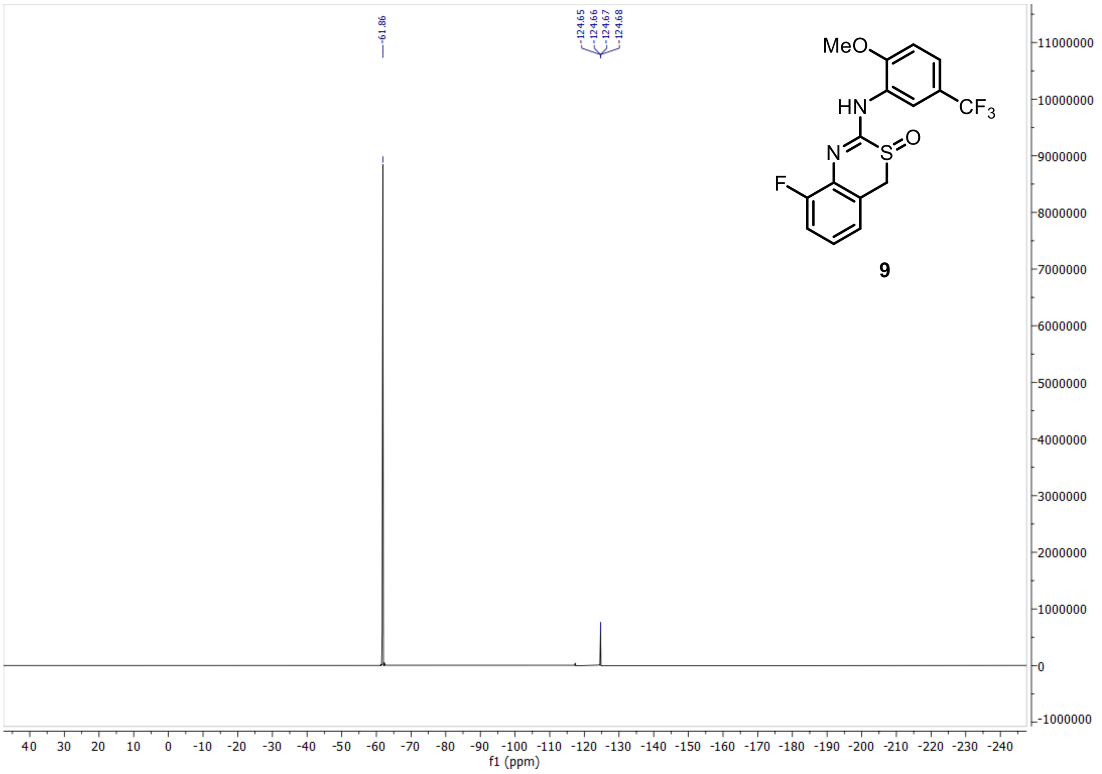

^{1}H NMR (400 MHz)

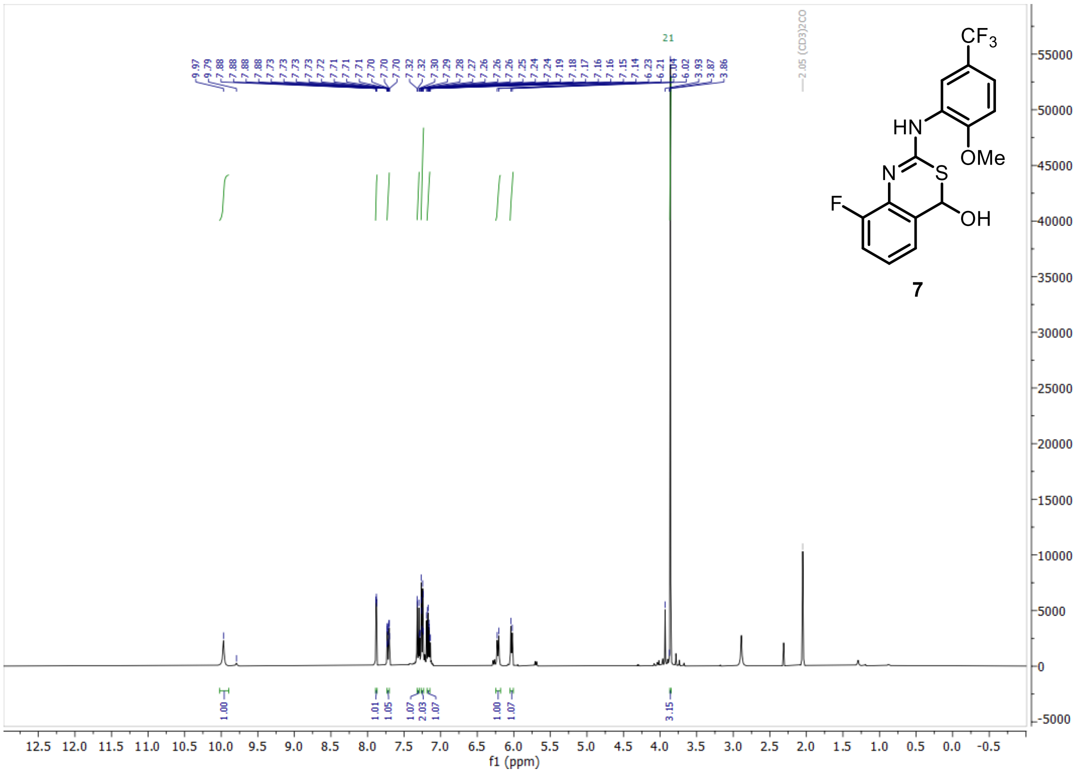

^{13}C NMR (101 MHz)

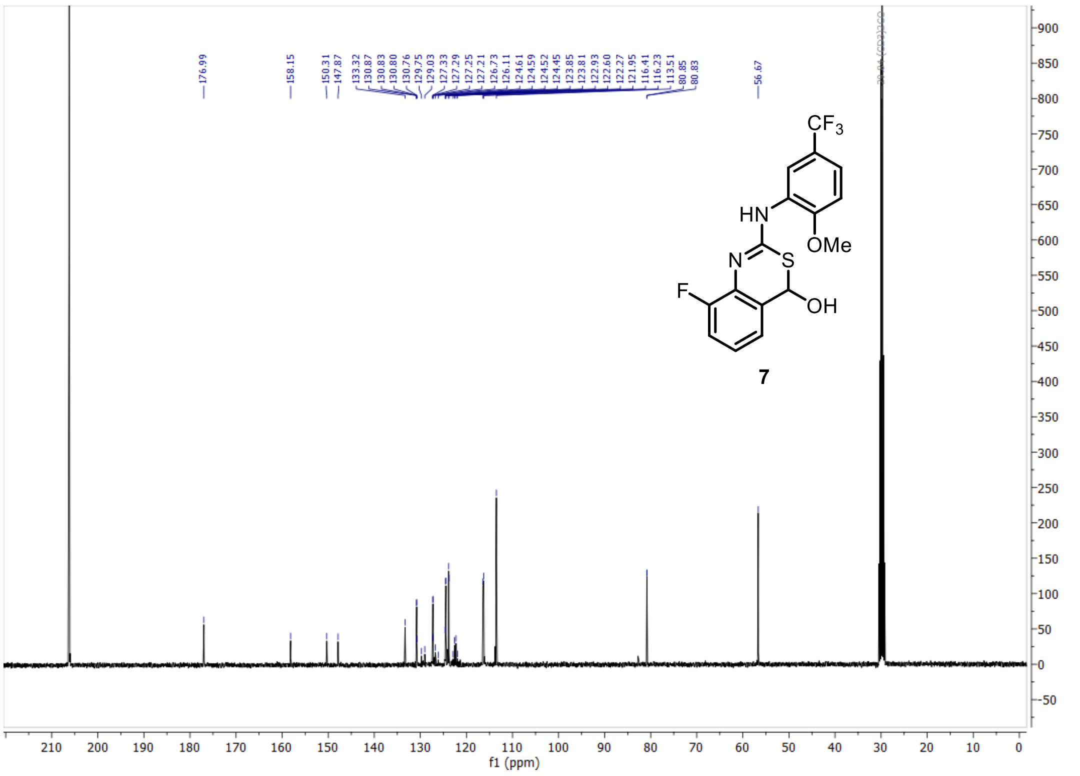

^{19}F NMR (377 MHz)

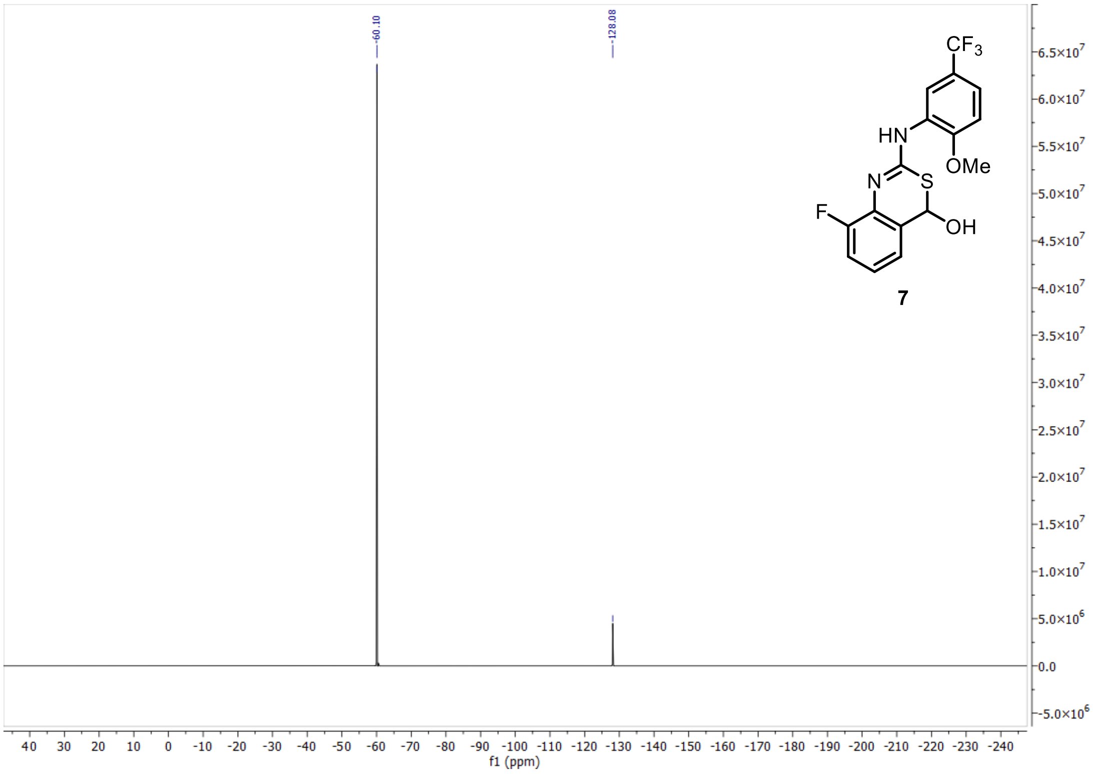

^{1}H NMR (400 MHz)

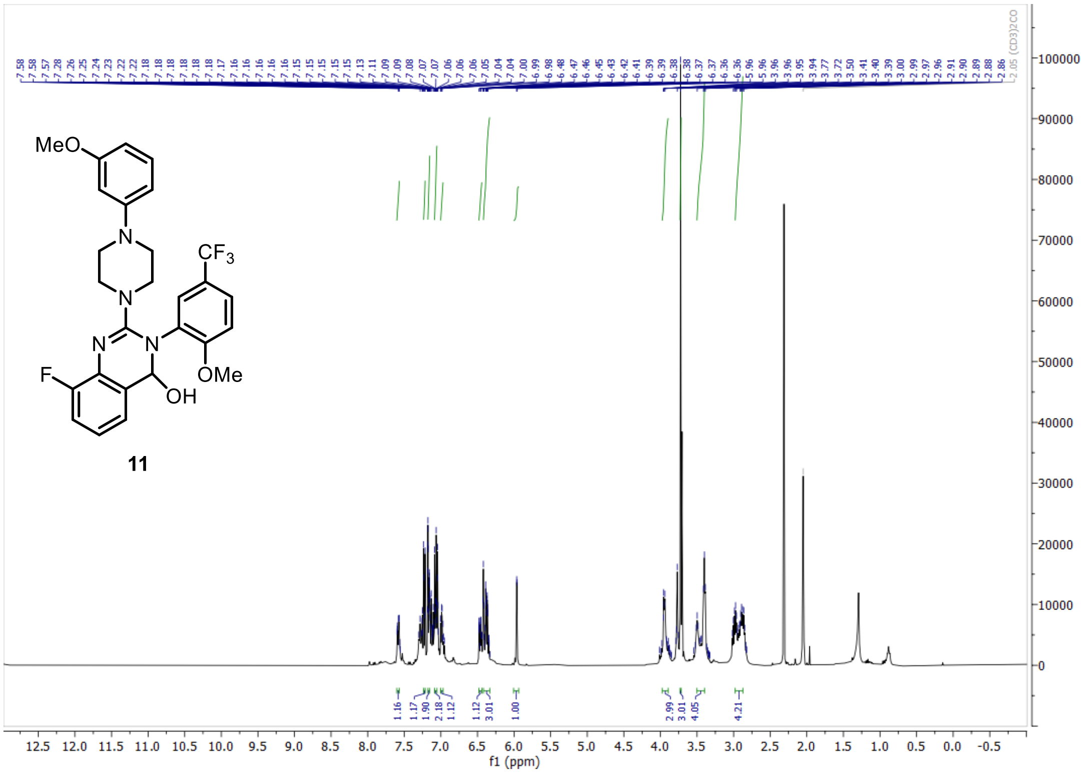

^{13}C NMR (101 MHz)

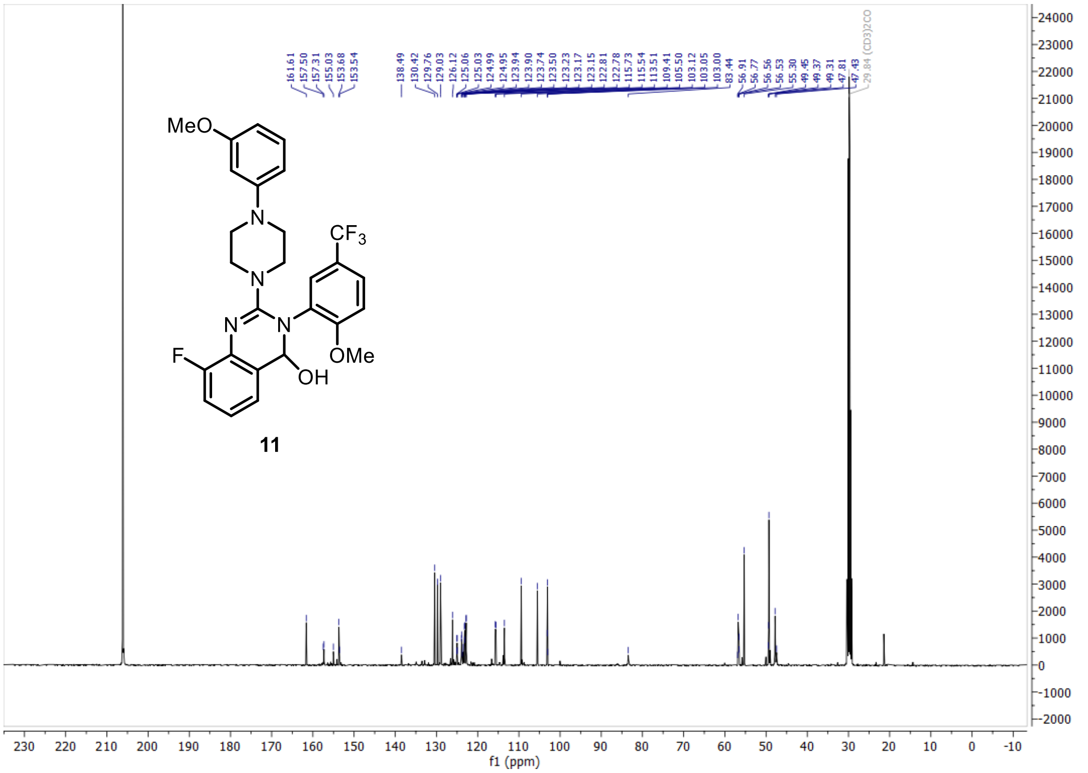

^{19}F NMR (377 MHz)

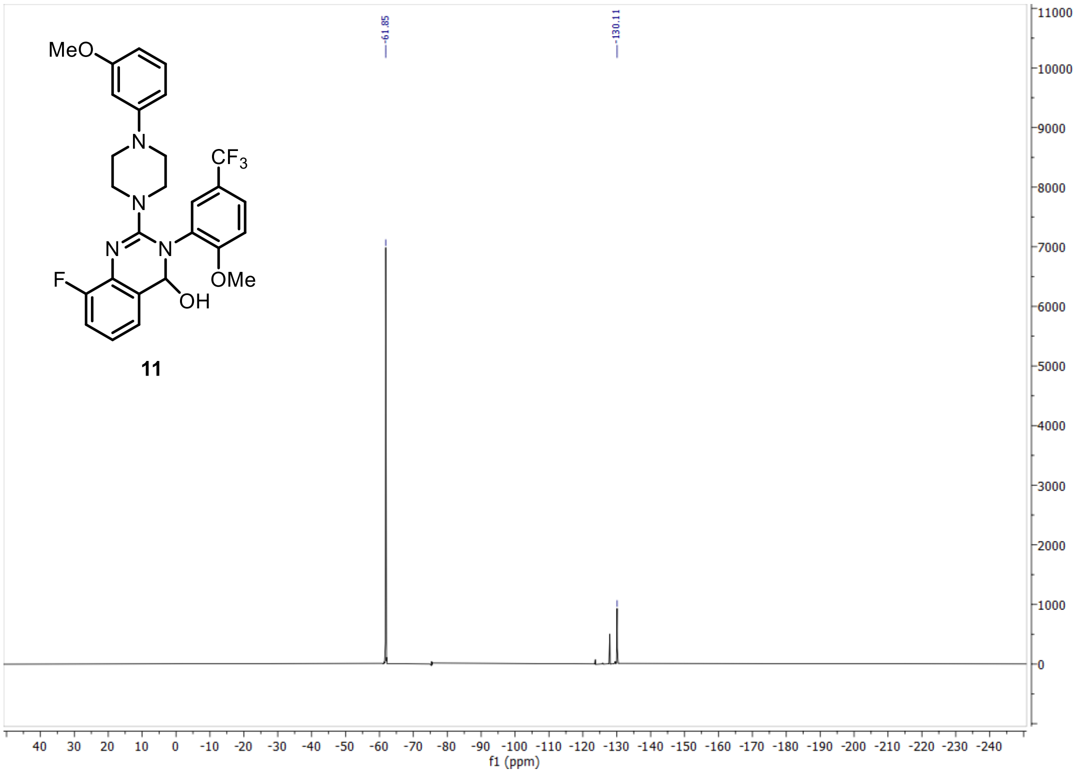

^{1}H NMR (400 MHz)

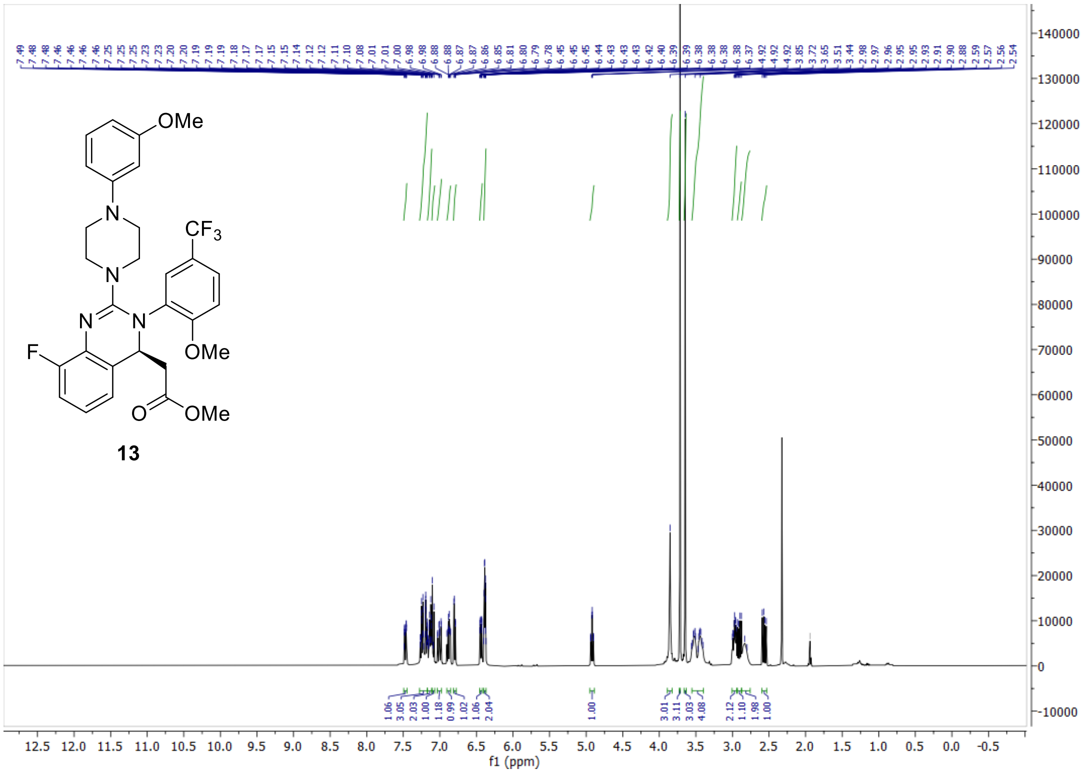

^{13}C NMR (101 MHz)

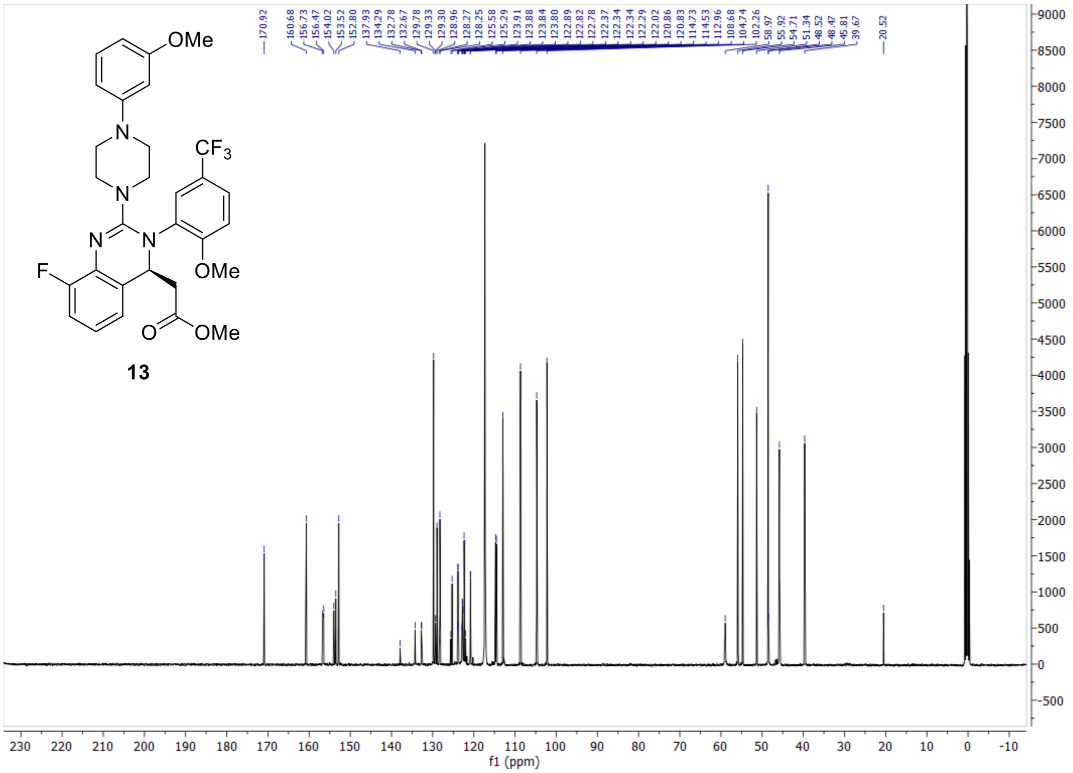

^{19}F NMR (377 MHz)

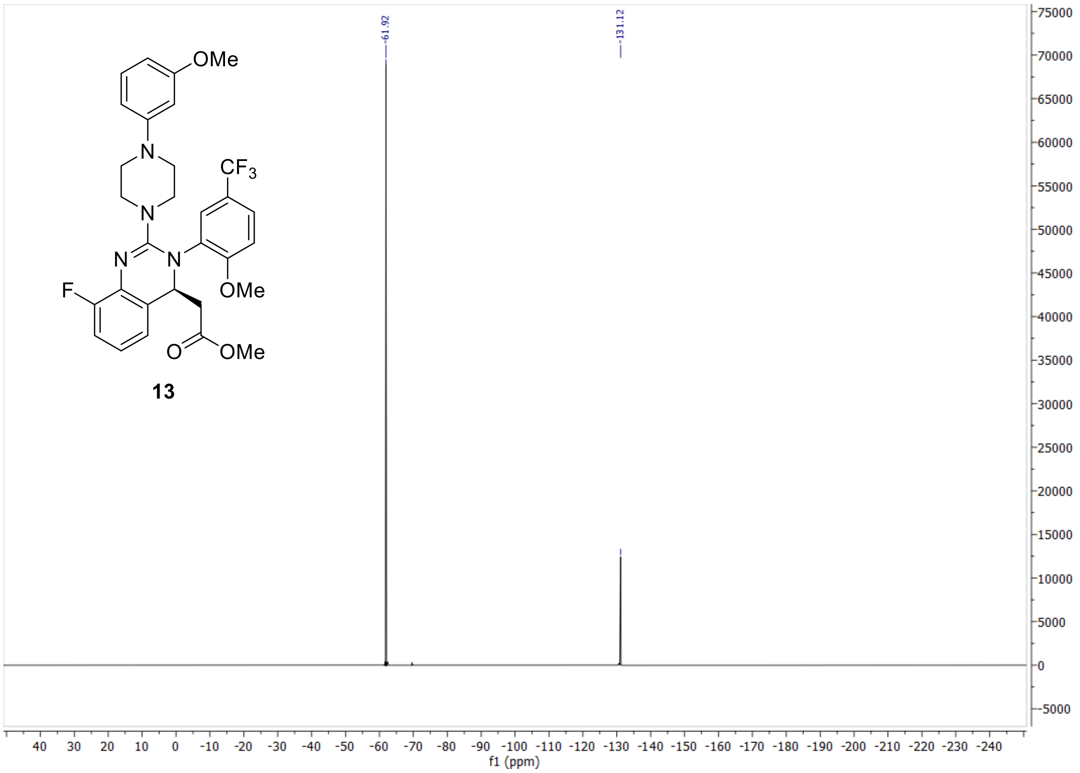

^{1}H NMR (400 MHz)

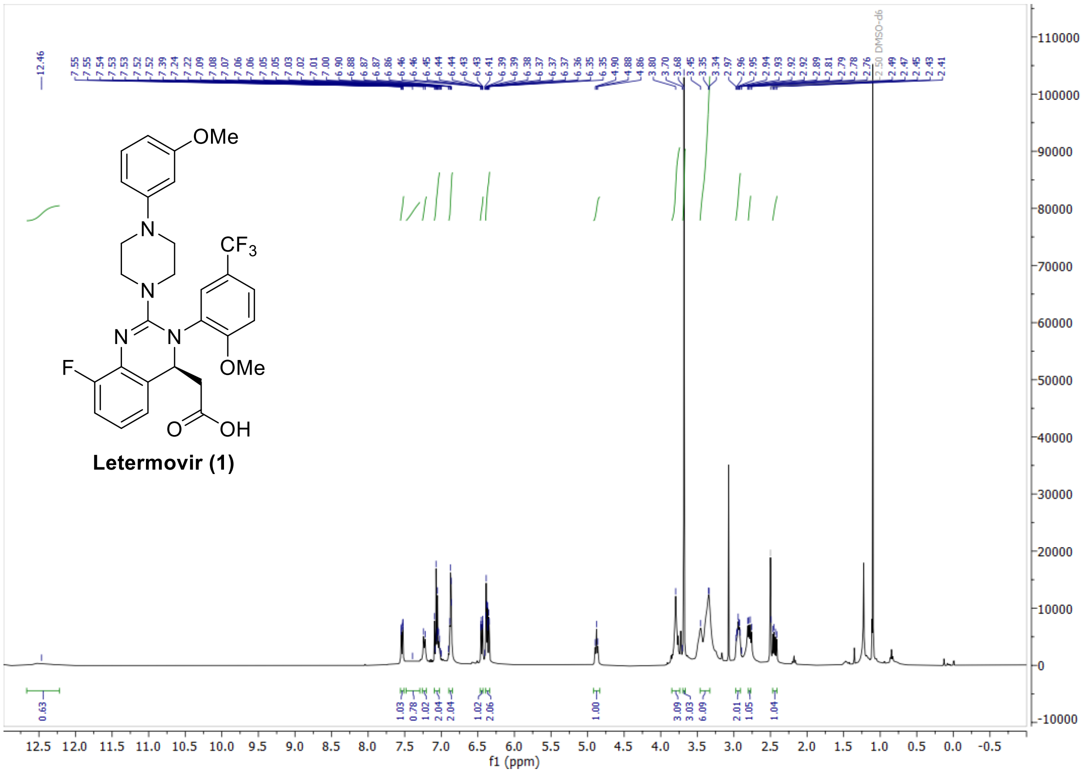

^{13}C NMR (101 MHz)

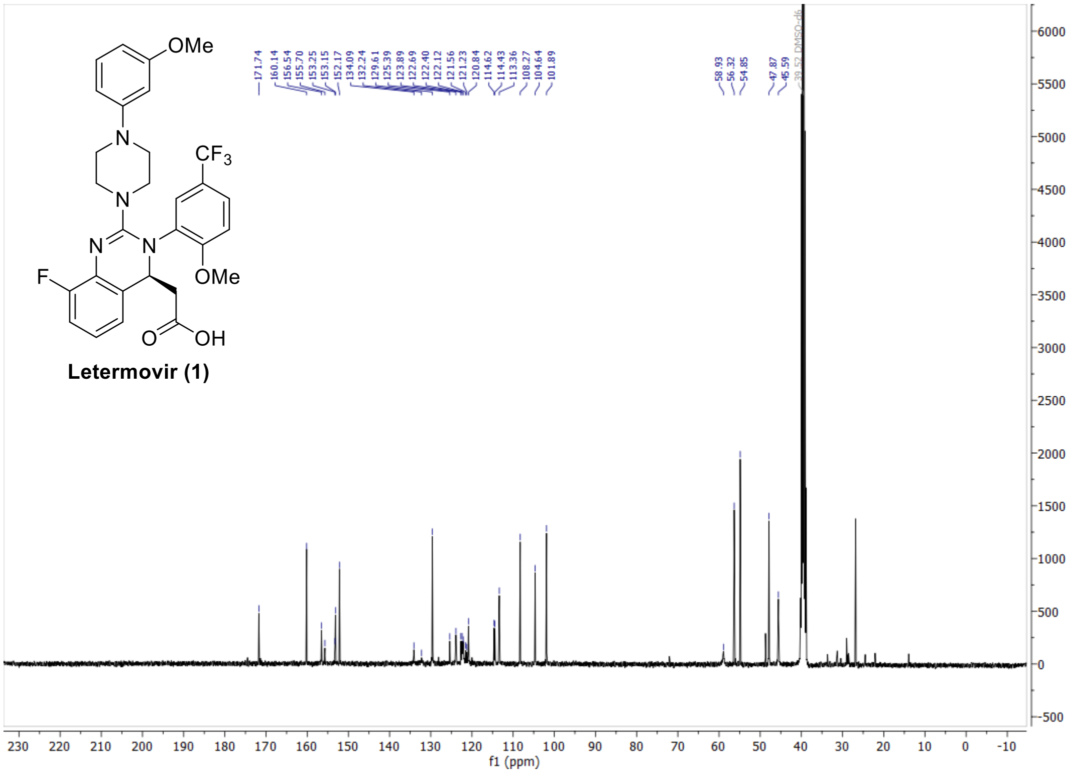

^{19}F NMR (377 MHz)

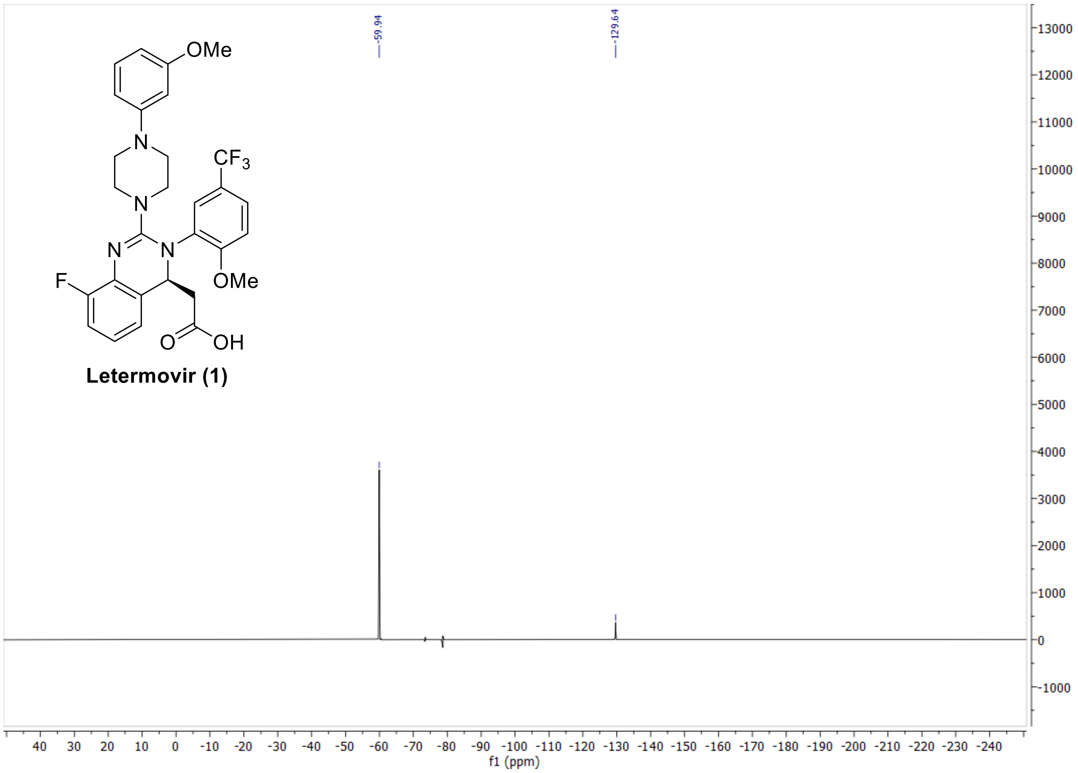

References:

1. Wasis, B.; Sandra, E., Ecological Study of Kina Tree (Cinchona spp.) and its Benefits in Overcoming the Spread of Malaria Disease. *Discussion material for Tropical Forest Nutrition Course* **2020**.
2. Levenspiel, O., *Chemical reaction engineering*. John wiley & sons: 1998.
3. Huijbregts, M. A. J.; Steinmann, Z. J. N.; Elshout, P. M. F.; Stam, G.; Verones, F.; Vieira, M.; Zijp, M.; Hollander, A.; van Zelm, R., ReCiPe2016: a harmonised life cycle impact assessment method at midpoint and endpoint level. *Int. J. Life Cycle Assess.* **2017,** *22* (2), 138-147.
4. Bergonzini, G.; Schindler, C. S.; Wallentin, C.-J.; Jacobsen, E. N.; Stephenson, C. R. J., Photoredox activation and anion binding catalysis in the dual catalytic enantioselective synthesis of β-amino esters. *Chemical Science* **2014,** *5* (1), 112-116.
5. Peng, B.; Ma, J.; Guo, J.; Gong, Y.; Wang, R.; Zhang, Y.; Zeng, J.; Chen, W.-W.; Ding, K.; Zhao, B., A Powerful Chiral Super Brønsted C–H Acid for Asymmetric Catalysis. *J. Am. Chem. Soc.* **2022,** *144* (7), 2853-2860.
6. Scharf, M. J.; List, B., A Catalytic Asymmetric Pictet–Spengler Platform as a Biomimetic Diversification Strategy toward Naturally Occurring Alkaloids. *J. Am. Chem. Soc.* **2022,** *144* (34), 15451-15456.
7. Dressler, F.; Öhler, V.; Topp, C.; Schreiner, P. R., Organocatalytic, Chemoselective, and Stereospecific House–Meinwald Rearrangement of Trisubstituted Epoxides. *Synlett* **2024,** *35* (09), 1052-1056.
8. Wenzel, A. G.; Jacobsen, E. N., Asymmetric Catalytic Mannich Reactions Catalyzed by Urea Derivatives: Enantioselective Synthesis of β-Aryl-β-Amino Acids. *J. Am. Chem. Soc.* **2002,** *124* (44), 12964-12965.
9. Londregan, A. T.; Burford, K.; Conn, E. L.; Hesp, K. D., Expedient Synthesis of α-(2-Azaheteroaryl) Acetates via the Addition of Silyl Ketene Acetals to Azine-N-oxides. *Org. Lett.* **2014,** *16* (12), 3336-3339.
10. Humphrey, G. R.; Dalby, S. M.; Andreani, T.; Xiang, B.; Luzung, M. R.; Song, Z. J.; Shevlin, M.; Christensen, M.; Belyk, K. M.; Tschaen, D. M., Asymmetric Synthesis of Letermovir Using a Novel Phase-Transfer-Catalyzed Aza-Michael Reaction. *Org. Process Res. Dev.* **2016,** *20* (6), 1097-1103.
11. Wang, P.-S.; Shen, M.-L.; Wang, T.-C.; Lin, H.-C.; Gong, L.-Z., Access to Chiral Hydropyrimidines through Palladium-Catalyzed Asymmetric Allylic C−H Amination. *Angew. Chem. Int. Ed.* **2017,** *56* (50), 16032-16036.
